# Supplementary material for: Computational models of episodic-like memory in food-caching birds
Source: Nat Commun. 2023 May 23;14:2979. doi: 10.1038/s41467-023-38570-x (PMC10205804; doi:10.1038/s41467-023-38570-x)
Supplement: Supplementary file 1 — Supplementary Information [file 41467_2023_38570_MOESM1_ESM.pdf]

Computational models of episodic-like memory in food-caching birds

Supplementary Information

Johanni Brea,[1,2*] Nicola S. Clayton,[3] Wulfram Gerstner[1.2]

[1]School of Computer and Communication Science,
École Polytechnique Fédérale de Lausanne, Lausanne, Switzerland
[2]School of Life Science,
École Polytechnique Fédérale de Lausanne, Lausanne, Switzerland
[3]Department of Psychology, University of Cambridge, Cambridge, UK

[*]To whom correspondence should be addressed; E-mail: johanni.brea@epfl.ch.

Contents

1 Supplementary Results **2**

1.1 Experimental Variability . . . 2
1.2 Fitting All Experiments Jointly . . . 2
1.3 Generalization . . . 3
1.4 Frequently Asked Questions . . . 3

2 Supplementary Figures **6**

2.1 Experiments . . . 11
2.1.1 Raby07 breakfastchoice . . . 11
2.1.2 Raby07 planningforbreakfast . . . 12
2.1.3 Cheke11 planning . . . 14
2.1.4 Correia07 exp2 . . . 16
2.1.5 deKort07 exp2 . . . 18
2.1.6 deKort07 exp3 . . . 20
2.1.7 deKort07 exp4 . . . 22
2.1.8 Clayton05 exp1 . . . 24
2.1.9 Clayton05 exp2 . . . 29
2.1.10 Clayton05 exp3 . . . 34

2.1.11 Clayton05 exp4 37
2.1.12 Clayton99A exp1 39
2.1.13 Clayton99A exp2 42
2.1.14 Clayton99B exp1 44
2.1.15 Clayton99B exp2 55
2.1.16 Clayton01 exp1 65
2.1.17 Clayton01 exp2 76
2.1.18 Clayton01 exp3 81
2.1.19 Clayton01 exp4 86
2.1.20 Clayton03 exp1 89
2.1.21 Clayton03 exp2 92
2.1.22 deKort05 102
2.1.23 Cheke11 specsat 107
2.1.24 Correia07 exp1 109
2.1.25 Clayton99C exp1 111
2.1.26 Clayton99C exp2 113
2.1.27 Clayton99C exp3 116
2.1.28 deKort07 exp1 119

1 Supplementary Results

1.1 Experimental Variability

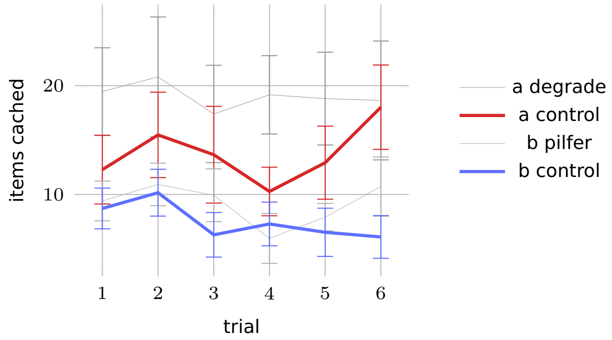

Figure S1: **Experimental Variability.** Evidence of variability across sessions and trials. In the experiment deKort07 exp1, the number of caches were counted in two sessions (a and b) of six trials under three conditions (degrade, pilfer, control; the control group is called "replenish group" in the original publication.). Under the same condition (control: red and blue) the counts in sessions a and b differ a lot. Even within the same session the counts can differ a lot between different trials (e.g. red curve trial 4 versus trial 6).

The birds' exact behaviour as observed in the experiments may depend not only on the experimental protocol but also on other factors such as the subset of individual birds used for an experiment, the species (western scrub jays were used for all experiments but the Cheke11 and Amodio21 experiments that used Eurasian jays), the season and the weather, social interactions of the tested birds with each other or the experimenter. The strong dependence on other factors is evident for example in the experiment deKort07 exp1 (Figure S1), where under the same conditions very different numbers of items were cached in different trials and different sessions.

A similar variability has been observed across two experiments in Clayton01 and Clayton99B. In both experiments, birds in the control group ("replenish") cached and inspected their caches under similar conditions, except that the inspection duration in Clayton01 exp1 was only 5 minutes compared to 15 minutes in Clayton99B exp1 and the spatial distance between the caches of different types of food was smaller in Clayton01 exp1 than in Clayton99B exp1. Despite a three-times shorter inspection period and on average less items cached, the birds in Clayton01 exp1 made almost twice as many inspections than the birds in Clayton99B exp1.

Our models capture some of the variability seen in the data with birds-specific parameters sampled from the fitted distributions (Figure 1B and Methods) and with stochastic action selection and random delays after each action (Methods). While more sophisticated noise models may capture even more variability we think that they are unjustified for the available data. Once future experiments provide more detailed data, modeling will be facilitated.

1.2 Fitting All Experiments Jointly

Our models can be fitted to all 28 experiments jointly, such that we have one set of hyper-parameters for all experiments. The main conclusions remain unchanged: first, the Plastic Caching Model is not worse than the Planning-By-Replay Model and, second, the lesioned models have lower reproducibility (Figure S2A) and likelihood (Figure S2B). The joint fits do not reproduce all experiments as well as the individual fits (Figure S2C).

Because of the observed variability in the experiments across trials, sessions and potentially seasons and subgroups of birds (cf. Experimental Variability) a joint fit of all experiments forces the model to increase the intrinsic noise (i.e., the variance of the distribution of parameters from which individual birds are chosen from or the variance of the action selection distributions of individual birds) which in turn lowers the overall average performance. In other words, some of the exact experimental results seem to depend on factors not included in our models. Therefore, the performance in a joint fit is expected to be lower compared to individual fits. The individual fits performed in the main text explicitly acknowledge that data is not expected to be consistent

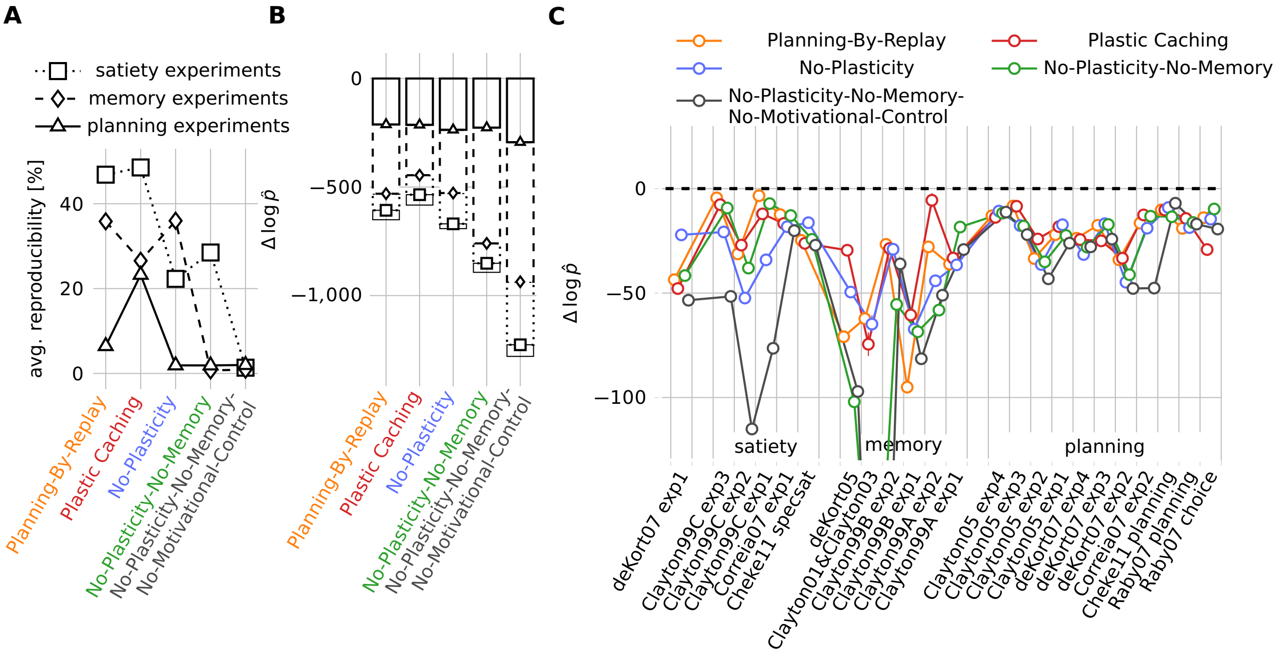

Figure S2: **Fitting all experiments jointly**. **A** Similarly to the results of the individual fits (Figure 2C, main text) the average reproducibility of planning, memory and satiety experiments drops almost to zero for lesioned models without plasticity, memory and motivational control, respectively. **B** The cumulative log-likelihood $\Delta \log \hat{p}$ relative to the same baseline as in Figure 2 of the main text (i.e. individual fits with the Planning-By-Replay Model) shows the same pattern with a drop of performance for the lesioned models. **C** The approximate log-likelihood $\Delta \log \hat{p}$ for each individual experiment reveals that some experiments are fitted almost as well as with individual fits (Figure 2E) whereas others are matched far worse. Which experiments are matched well depends on the random seed of optimization: because the optimization problem is non-convex, two different values for the fitted hyper-parameters may lead to the same cumulative performance but very different performances for the individual experiments.

across all 28 experiments.

1.3 Generalization

Fitting the models on a subset of experiments (training set) and evaluating them on test experiments leads to results consistent with previous findings: the Plastic Caching Model and the Planning-By-Replay Model generalize best and performance degrades for the models with lesions (Figure S3). Given the findings in Experimental Variability we expect that the Plastic Caching Model and the Planning-By-Replay Model capture the trend of the data but fail to match the exact numbers. This is confirmed by a higher average reproducibility (see Methods) for these models than for the lesioned models (Figure S3A1,A2 & B1, B2). Since the Plastic Caching Model and the Planning-By-Replay Model reproduce only the trend, but not absolute numbers, of the data, the values for $\Delta \log \hat{p}$ are low, despite the fact that qualitatively the main trends are captured (Figure S3A3 & B3).

1.4 Frequently Asked Questions

Are the differences between the models significant?

We do not quantify or claim significance for the difference between the Plastic Caching Model and the Planning-By-Replay Model. However, models with lesions fail clearly and qualitatively on some of the experiments (see Figure 2C-E in the Main Text and Experiments).

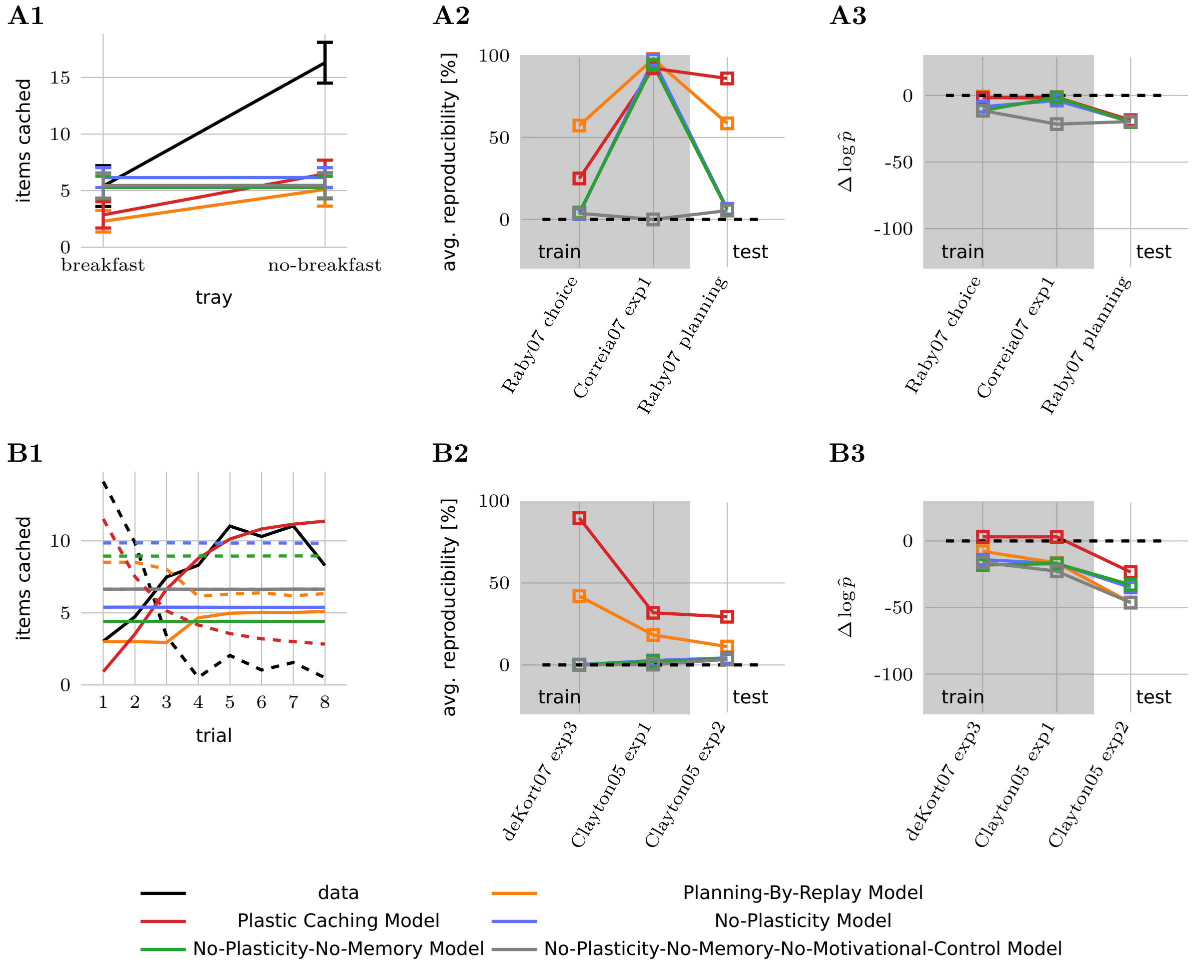

Figure S3: **Generalization.** The models were fitted on two training experiments jointly and evaluated on one test experiment. **A1, B1** The main trend in the data of the test experiment (black solid line: nuts cached and black dashed line: worms cached by birds that experienced degraded worms at recovery) is on average better captured by the full models (colored lines = simulation results averaged over 10^5 simulations, error bars = average SEM). **A2, B2** This is reflected by the average reproducibility of the most relevant statistical tests, which is higher for the Plastic Caching Model and the Planning-By-Replay Model than for the simpler models. **A3, B3** The performance $\Delta \log \hat{p}$ (measured relative to the same baseline as in Figure 2 of the main text, i.e. individual fits with the Planning-By-Replay Model) depends not only on the trends, but also on the absolute numbers; it is therefore lower than in the individual fits.

Do the models overfit the data?

Yes, our models potentially overfit some aspects (see subsection 1.2 and subsection 1.3). Some unmeasured, relevant factors of variability are not included in our models (as explained above) and it is unlikely that the amount of available data assures that these factors average out.

However, our main conclusions do not depend on whether the models overfit the data or not: the Plastic Caching Model is not worse than the Planning-By-Replay Model on any experiment, thus explicit planning does not seem to be necessary to match the experimental results, and each model with lesions fails at reproducing on average some of the experiments (see Figure 2 in the Main Text and Supplementary Text).

What is the number of parameters?

Note that the numbers of free parameters are only upper bounds to the effective numbers of free parameters. For example, parameters that influence caching and inspection behaviour have no influence on the results

model	parameters per food type	other parameters	1 food type	2 food types
No-Plasticity-No-Memory-No-Motivational-Control Model	0	16	16	16
No-Plasticity-No-Memory Model	6	20	26	32
No-Plasticity Model	6	24	30	36
Plastic Caching Model	6	34	40	46
Planning-By-Replay Model	6	34	40	46

Table S1: Number of parameters for each model. The last two columns show the total number of parameters for an experiment with one or two food types, respectively. The numbers of parameters are multiples of two, because we parameterize each beta distribution with two parameters.

of experiments where the birds were not allowed to cache any food. Therefore, increasing the number of free parameters does not necessarily imply a higher number of effective free parameters and a higher degree of overfitting. We see clear evidence that the number of free parameters is less crucial than the available components of the model: all models with lesions make similar predictions for the deKort07 and Raby07 experiments, despite large differences in the number of free parameters, but none of these models captures on average the trends seen in the data (see subsection 2.1). In general, measuring the effective number of free parameters is not trivial.

What is the AIC/BIC value of the models?

The Akaike Information Criterion (AIC) and the Bayesian Information Criterion (BIC) depend on the number of free parameters of a model. Because of the above mentioned difficulties in counting the effective number of free parameters we did not compute AIC or BIC values.

2 Supplementary Figures

	**full observability**<br>The observed state contains all relevant information for decision making; the history of observations does not contain more information.	**partial observability**<br>the observed state contains only partial information for decision making; the history of observations contains more information.
**model-free reinforcement learning**<br>The agent adapts directly the parameters of the decision making policy, without inferring the transition structure ‘observation & action → new observation’, known as the model of the environment.	• Models of Operant Conditioning<br>• Q-Learning, SARSA<br>• Policy Gradient	• Memory-augmented versions of models of Operant Conditioning, Q-Learning, SARSA and Policy Gradient, e.g. Policy Gradient with Recurrent Neural Networks<br>• **Our Plastic Caching Model**
**model-based reinforcement learning**<br>The agent infers the transition structure ‘observation & action → new observation’ of the environment and uses this model to update the policy in a planning step.	• Models of Goal-Directed Behaviour<br>• DYNA Architecture<br>• MDP Solvers	• Memory-augmented versions of models of Goal-Directed Behaviour and the DYNA Architecture, POMDP Solvers<br>• Models of mental time-travel and creative recombination of past experiences.<br>• **Our Planning-By-Replay Model**

Figure S4: **Categorization of Reinforcement Learning Models.** In reinforcement learning we can classify methods based on their ability to learn a model of the environment (model-free versus model-based) and their ability to deal with partial observability (full observability versus partial observability). A model of the caching behavior of corvids needs to address the partial observability of the cache contents; it is thus in the right column. Our Plastic Caching Model is a model-free reinforcement learning model. Our Planning-By-Replay Model is a very simple model-based reinforcement learning model in that the experienced transitions are stored in memory and used at decision time to replay sequences that appeared in a similar context as the current one.

Procedure for the 'planning for breakfast' experiment. Each bird received six training trials followed by one test trial. From 09:00–17:00 each day the birds maintenance diet was freely available. At 17:00 the test subject was separated from its companion into compartments A, B and C. Maintenance diet was removed for 90 min at 17:00 and caching trays were put in compartments A and C. At 18:30 a bowl containing 10 g of powdered pine nuts, which could be eaten but not cached, was put in the centre of compartment B for 30 min. At 19:00 the bowl of pine nuts and the caching trays were removed. The birds were deprived of food overnight so that they were mildly hungry in the morning. Each morning at 07:00 the experimental bird was confined in either compartment A or C for two hours. The caching trays were returned to both compartments A and C for this period. In one compartment the bird was given no food ('no-breakfast' compartment) and in the other the bird was given powdered pine nuts ('break- fast' compartment). Each bird experienced the two compartments on alternate days, with the compartment that was the 'breakfast' compartment (A or C) counterbalanced across birds. The order in which each bird experienced the two compartments was also counterbalanced. The test day followed exactly the same evening routine as training days with the exception that 30 (cacheable) whole pine nuts were given at 18:30 instead of powdered food. The jays did not get the opportunity to recover their caches on the morning after the test trial.

```
function run_experiment(::Experiment{:Raby07_planningforbreakfast},
                        models; N = 8) # 8 birds in total
  results = DataFrame(tray = [], id = [], items = [])
  for id in 1:N
    m = models[id]
    trays = [Tray(i) for i in 1:2] # caching trays in compartments A and C
    for day in 1:7
      add!(m, MaintenanceDiet) # start at 9am on first day
      wait!(m, 8u"hr")
      remove!(m, MaintenanceDiet)
      add!(m, trays)
      wait!(m, 1.5u"hr")
      if day == 7
        add!(m, Pinenut, 30)
      else
        add!(m, PowderedPinenut, 30)
      end
      wait!(m, 30u"minute")
      if day == 7
        push!(results, ["breakfast", id, countcached(trays[1])])
        push!(results, ["no-breakfast", id, countcached(trays[2])])
        break # end of experiment
      end
      remove!(m, Any)
      wait!(m, 12u"hr")
      if (id + day) % 2 == 0 # counterbalancing
        add!(m, PowderedPinenut, 30)
        add!(m, trays[1])
      else
        add!(m, trays[2])
      end
      wait!(m, 2u"hr")
      remove!(m, Any)
    end
  end
  results
end
```

Figure S5: **Example of a formalized protocol.** The protocol as reported by Raby et al. [10] (reprinted on the left) is transformed into a function (on the right) that takes N models of a bird and its cage as input and returns a result in the form of a table (DataFrame). Highlighted in dark blue are the terms of the domain specific language and in light blue the control commands of the programming language Julia. For example the command add!(m, PowderedPinenut, 30) tells that an object of type Food with properties 'freshness = 1', 'eatable = true', 'cacheable = false' (because it is powdered), 'amount = 30' and 'type = pinenut' should be added to the simulated cage m (see also Methods).

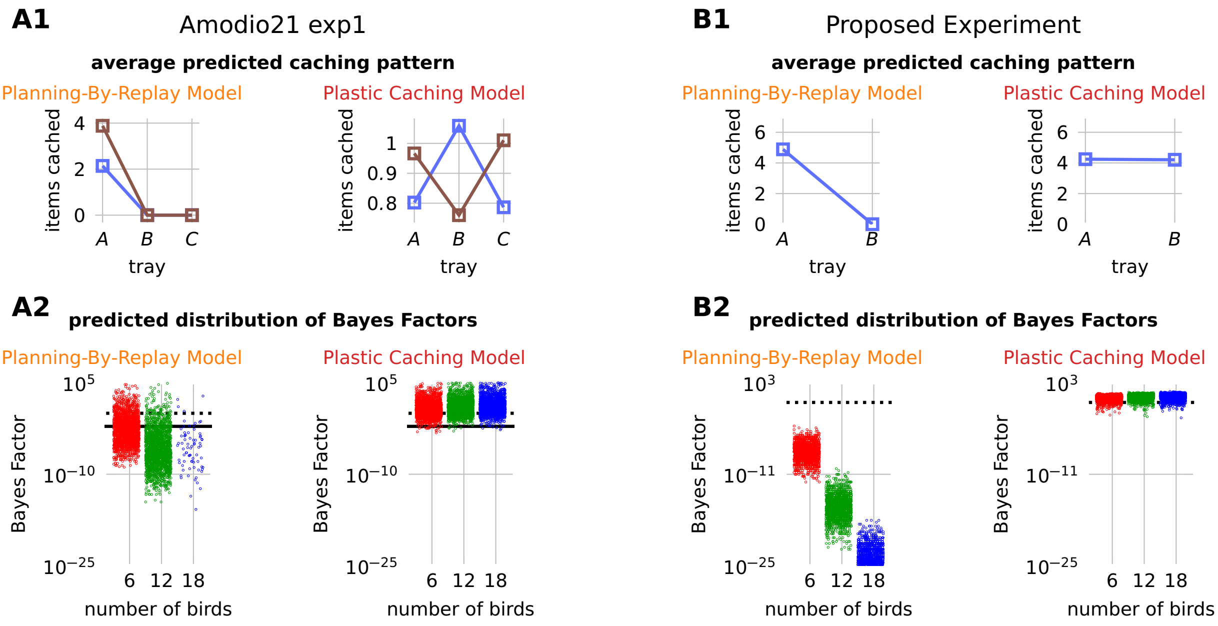

Figure S6: **Using the model and the domain-specific language to design new experiments.** Although the Plastic Caching Model matches some experimental results quantitatively better than the Planning-By-Replay Model, both models make qualitatively the same predictions on the 11 planning experiments so that a clear distinction between the models is not yet possible. To design more stringent tests, with experiments where the two models make qualitatively different predictions, we can leverage the domain specific language of experimental protocols and simulate expected outcomes before running the actual experiments. **A1** The Planning-By-Replay Model and the Plastic Caching Model make qualitatively different predictions on the caching pattern of two groups of birds (blue and brown) in the experimental protocol of Amodio et al. 2021 [44]. **A2** However, the effect is not strong as shown by the distribution of Bayes factors [44] after 2000 simulations of the experiment with 6 birds (one red dot per simulation) that overlap for both models with the experimental data (thick black line). Running the same experiment with 12 or 18 birds only slightly improves the situation. The Bayes Factor for 18 birds with the Planning-By-Replay Model is below 10^{-25} for most simulations, but there is a tail (blue dots) overlapping with the experimental data. **B1** Predicted average caching pattern of a proposed experiment (see supplementary Figure S4). **B2** The distributions predicted by the two models have little overlap, suggesting that it should be possible to discriminate the two models even in an experiment with only 6 birds.

```
function run_experiment(::Experiment{:Hypothetical}, models)
    results = DataFrame(id = Int[], action = String[], A = Int[], B = Int[])
    for id in eachindex(models)
        m = models[id]
        trays = [Tray(i) for i in 1:4] # C, D, A, B
        for day in 1:9
            cid = floor(Int, ((day-1)) % 2 + 1)
            add!(m, trays[cid]) # compartment to spend the day from 10am onwards
            add!(m, MaintenanceDiet)
            wait!(m, 6.75u"hr")
            remove!(m, Any)
            if day == 9
                add!(m, trays[3:4])
                add!(m, Kibble, 10) # test
                wait!(m, 15u"minute")
                remove!(m, Any)
                push!(results, [id; "cache"; countcache.(trays[3:4])])
                break # end of experiment
            else
                add!(m, trays[3:4]) # access to caching trays in A and B
                add!(m, PowderedKibble, 10) # without option of caching
                wait!(m, 15u"minute")
                remove!(m, Any)
                add!(m, MaintenanceDiet)
                wait!(m, 3u"hr")
            end
            remove!(m, Any)
            wait!(m, 12u"hr") # morning
            add!(m, trays[cid == 1 ? 3 : 4]) # add (empty) caching trays to the compartment where the birds wakes up hungry
            wait!(m, 1u"hr")
            remove!(m, Any)
            wait!(m, 1u"hr")
            add!(m, MaintenanceDiet)
        end
    end
    results
end
```

Figure S7: **The proposed experiment in Fig S3B**. It is based on a modification of the experimental protocol of [10] (c.f. Figure S5). Birds spend their day after 10am in compartments C or D. If they spend their days in C they are transferred to A in the evening and stay there until 10am of the next morning; if they spend their day in D they will sleep and wake up in B. Neither in A nor in B they receive any food, such that both compartments are equally associated with being hungry. After sufficiently many repetitions of C → A and D → B, the birds are given the chance to cache food in A or B, in the evening, after having spent their day in C or D. The Plastic Caching Model distributes caches equally between A and B, because both compartments are equally associated with being hungry and the model does not learn the transition structure of the world (Fig. S1). In contrast, the Planning-By-Replay Model takes into account the learned model of the world (associations C → A and D → B) and caches more food in A after a day in C and more in B after a day in D. Hence this new protocol should enable falsification of one of the two models.

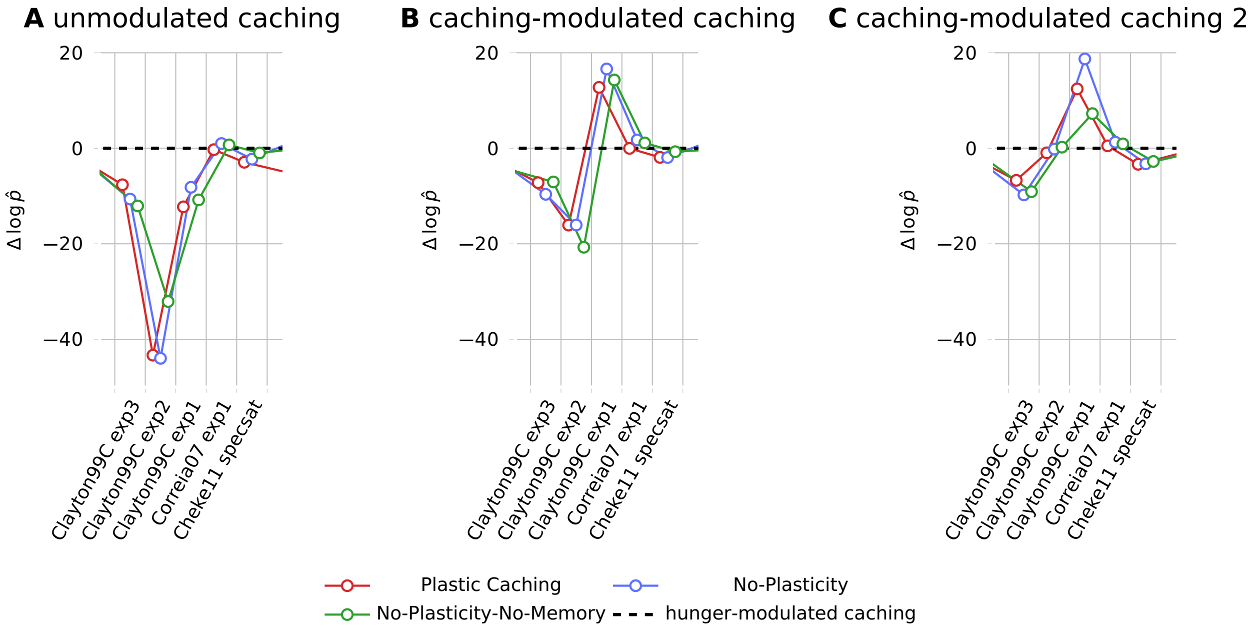

Figure S8: **Alternative models of motivational control of caching**. **A** The experiments in Clayton et al. (1999) [24] cannot be reproduced if caching does not depend on any motivational state and thus is independent of the recent eating and caching history. For each model the estimated log-likelihood is shown relative to a version of the same kind of model with hunger-modulated caching. **B** If caching depends on a low-pass filter of the recent caching history, the first experiment in Clayton et al. (1999) [24] is reproduced better than with hunger-modulated caching, but the other experiments are reproduced worse. In experiments Clayton99C exp3 and Cheke11 specsat, birds that ate to satiety on uncacheable, powdered food, cached subsequently fewer cacheable items of the same kind of food than birds under control conditions. This behavior is not reproducible with caching-modulated caching. **C** If caching-modulated caching is modelled with filters of the same kind as the hunger variables (instead of a simple low-pass filter), the results of Clayton99C exp2 can also be reproduced, but the experiments Clayton99C exp3 and Cheke11 specsat remain unreproducible.

2.1 Experiments

In the following we present brief summaries and result figures for all considered experiments. The figures in the center with title "Data" contain the measured values extracted from the published experiments. The figures on the right contain averaged simulated results, obtained by running the same simulated experiment 10^5 times and averaging the results. The error bars indicate average standard errors of the mean. The figures on the left contain the simulated result closest to the experimental result (closest out of 10^5 different simulated runs).

2.1.1 Raby07 breakfastchoice

Published in [10].

Major Finding

Birds cache more items of the food type that is different to the one they experienced to have available in the morning in a given compartment.

Most Important Tests

More different food cache (foodtype): $F(1,8) = 2.29, p = 0.17$

More different food cache (tray): $F(1,8) = 5.48, p = 0.047$

Total Number of Statistics

2 p-values, 8 means or sems.

Figures

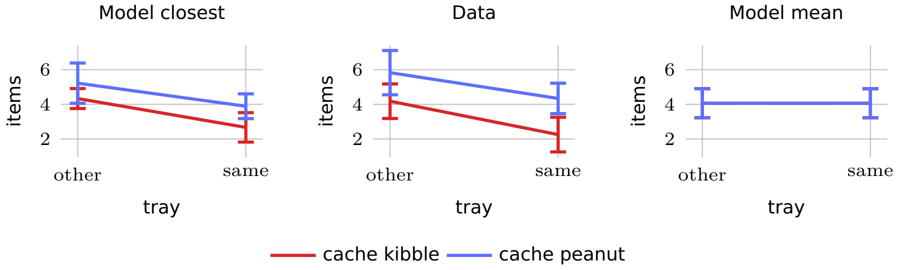

Figure S9: **Raby07 breakfastchoice: No-Plasticity-No-Memory-No-Motivational-Control Model**

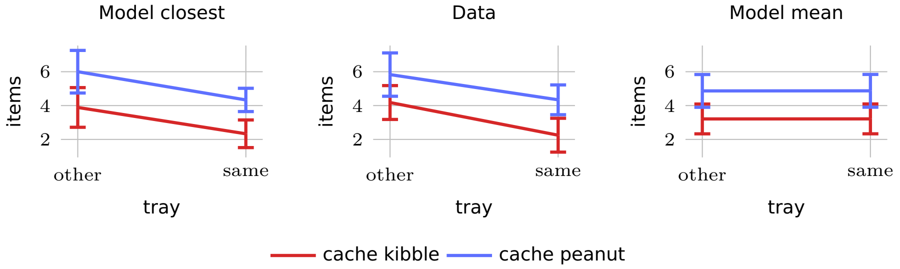

Figure S10: **Raby07 breakfastchoice: No-Plasticity-No-Memory Model**

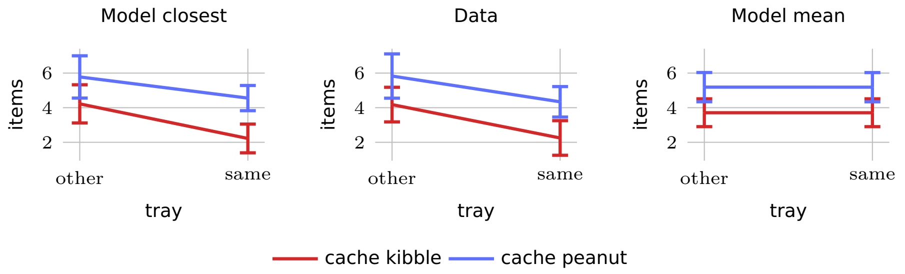

Figure S11: **Raby07 breakfastchoice: No-Plasticity Model**

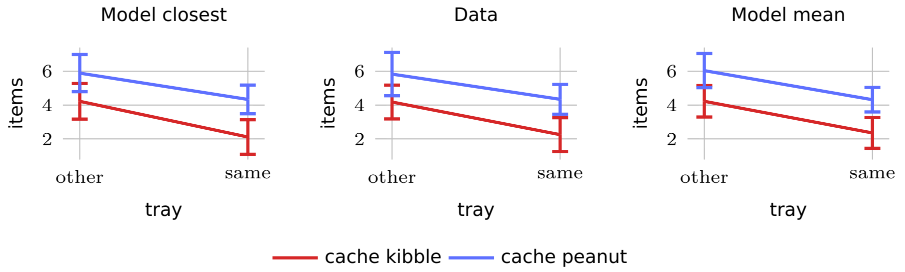

Figure S12: **Raby07 breakfastchoice: Plastic Caching Model**

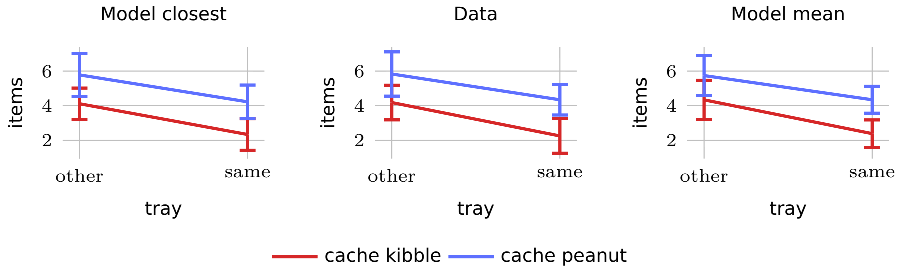

Figure S13: **Raby07 breakfastchoice: Planning-By-Replay Model**

2.1.2 Raby07 planningforbreakfast

Published in [10].

Major Finding

Birds cache more at sites where they experienced to be hungry in the morning.

Most Important Tests

More pine nuts in no-breakfast compartment (tray): $F(1,7) = 9.06, p = 0.02$

Total Number of Statistics

1 p-values, 4 means or sems.

Figures

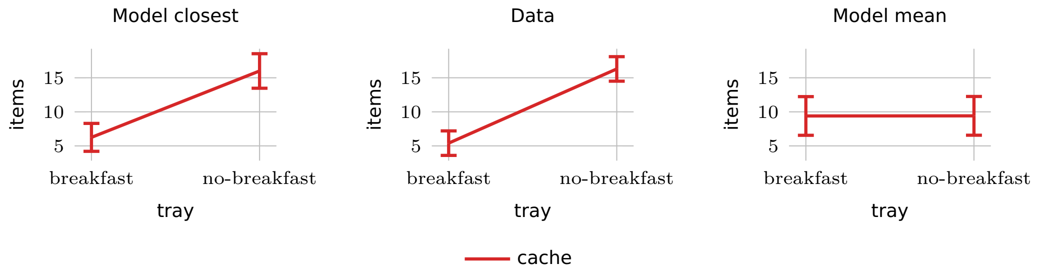

Figure S14: **Raby07 planningforbreakfast: No-Plasticity-No-Memory-No-Motivational-Control Model**

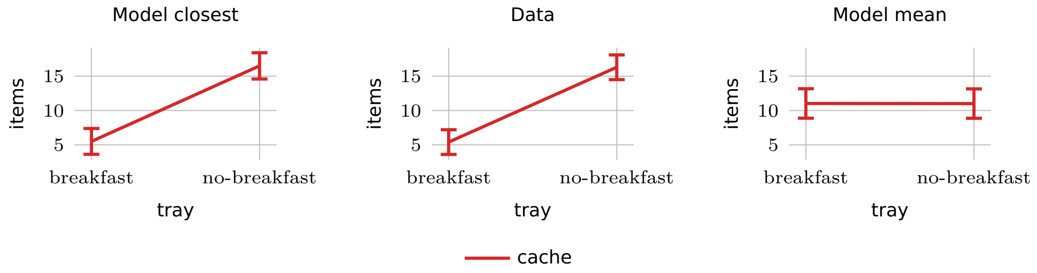

Figure S15: **Raby07 planningforbreakfast: No-Plasticity-No-Memory Model**

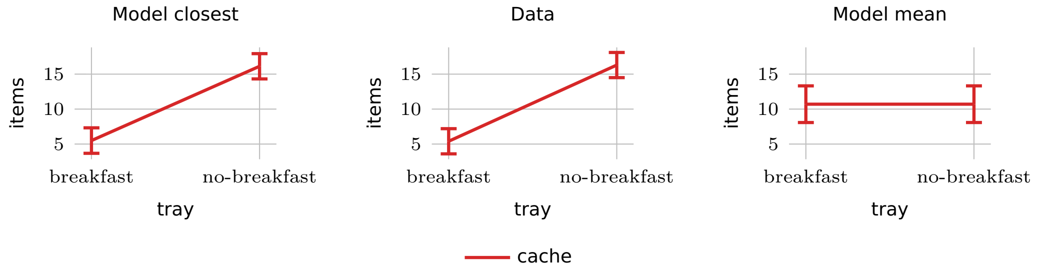

Figure S16: **Raby07 planningforbreakfast: No-Plasticity Model**

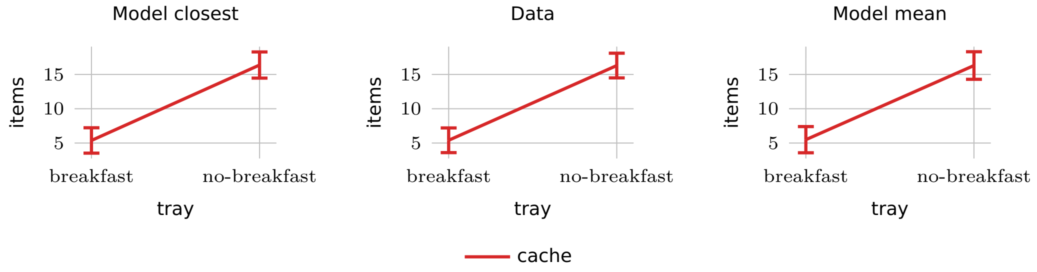

Figure S17: **Raby07 planningforbreakfast: Plastic Caching Model**

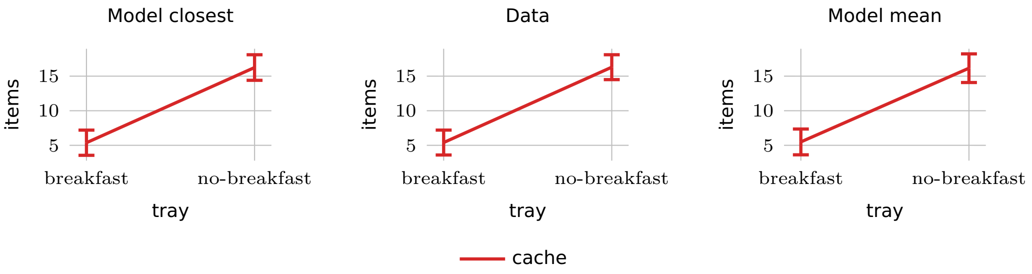

Figure S18: **Raby07 planningforbreakfast: Planning-By-Replay Model**

2.1.3 Cheke11 planning

Published in [12].

Major Finding

Birds cached both foods in equal amounts in both trays in trial 1 but then developed a differential preference between the trays, preferentially caching in each tray the type of food that they would desire when retrieving from it.

Comments

Since it is unclear how the tests for the overall reduction in caching and the trial × tray × foodtype were performed, we omitted these tests. This experiment used Eurasian jays.

Most Important Tests

Tray × foodtype interaction in trial 2 (tray & foodtype): $F(1, 3) = 14.24, p = 0.033$

Tray × foodtype interaction in trial 1 (tray & foodtype): $F(1, 3) = 3.0, p = 0.18$

Tray × foodtype interaction in trial 3 without hunter (tray & foodtype): $F(1, 2) = 15.429, p = 0.059$

Total Number of Statistics

6 p-values, 24 means or sems.

Figures

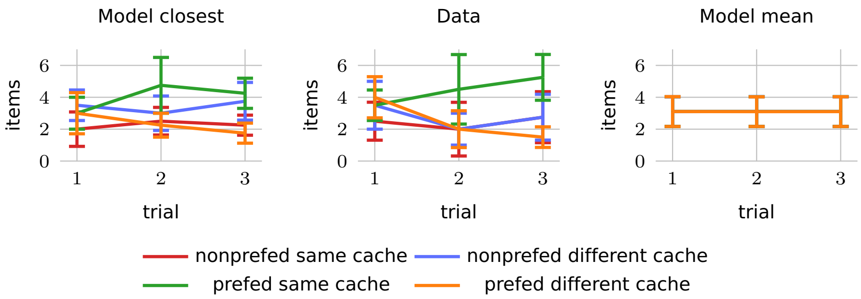

Figure S19: **Cheke11 planning: No-Plasticity-No-Memory-No-Motivational-Control Model**

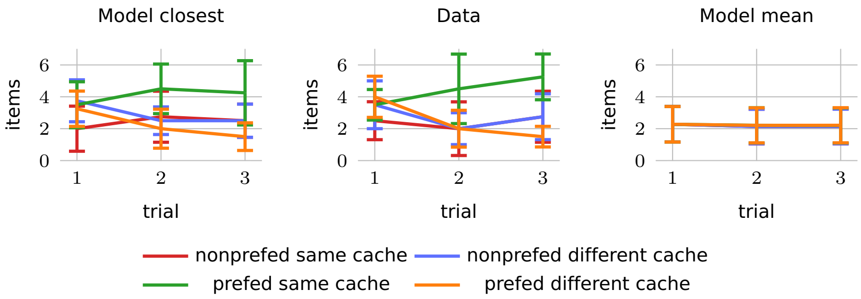

Figure S20: **Cheke11 planning: No-Plasticity-No-Memory Model**

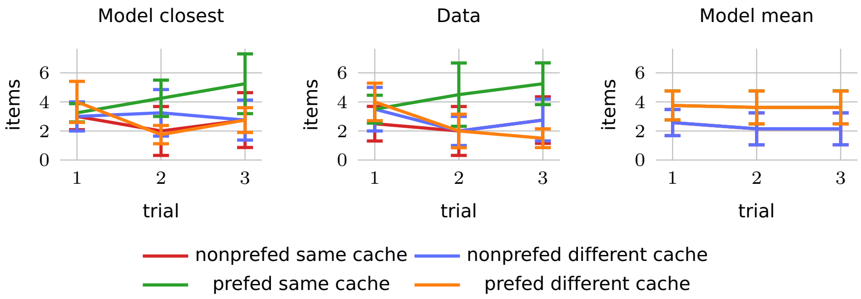

Figure S21: **Cheke11 planning: No-Plasticity Model**

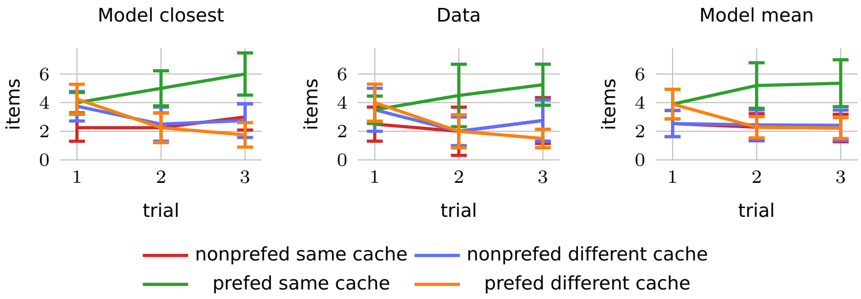

Figure S22: **Cheke11 planning: Plastic Caching Model**

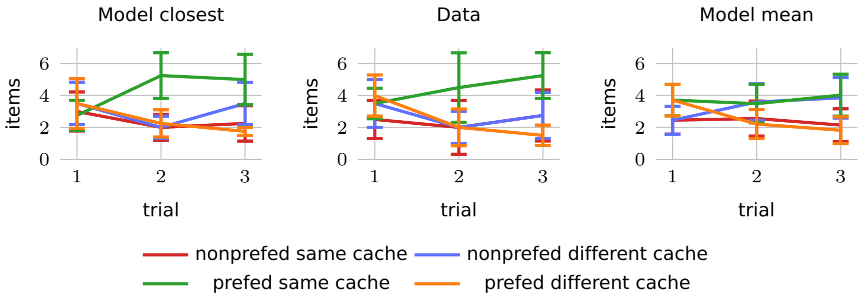

Figure S23: **Cheke11 planning: Planning-By-Replay Model**

2.1.4 Correia07 exp2

Published in [11].

Major Finding

The birds learn to cache more of the food type they will not be prefed on prior to recovery.

Most Important Tests

trial 1: proportion cache (group): $U(10) = .11, p = 0.11$

trial 3: proportion cache (group): $U(8) = 0., p = 0.0$

trial 2: proportion cache (group): $U(8) = .04, p = 0.04$

Total Number of Statistics

12 p-values, 48 means or sems.

Figures

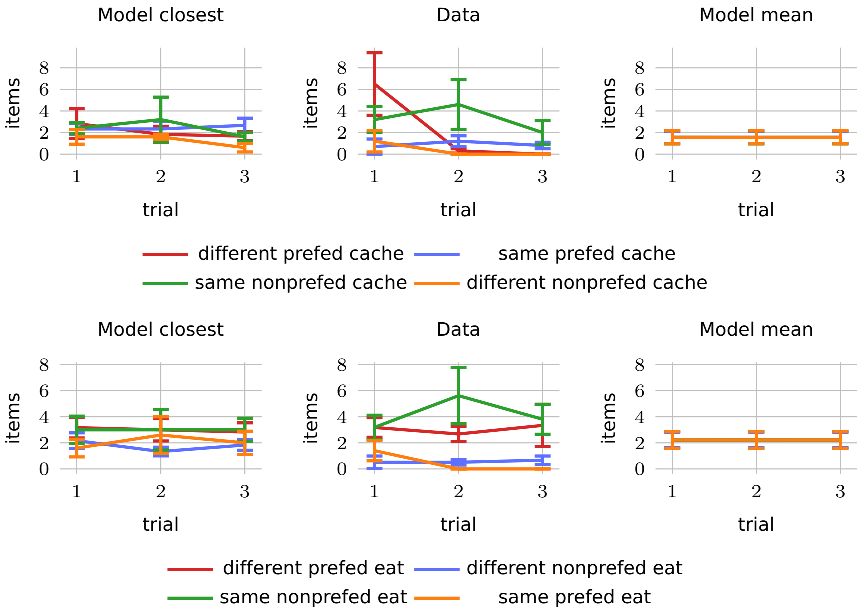

Figure S24: **Correia07 exp2: No-Plasticity-No-Memory-No-Motivational-Control Model**

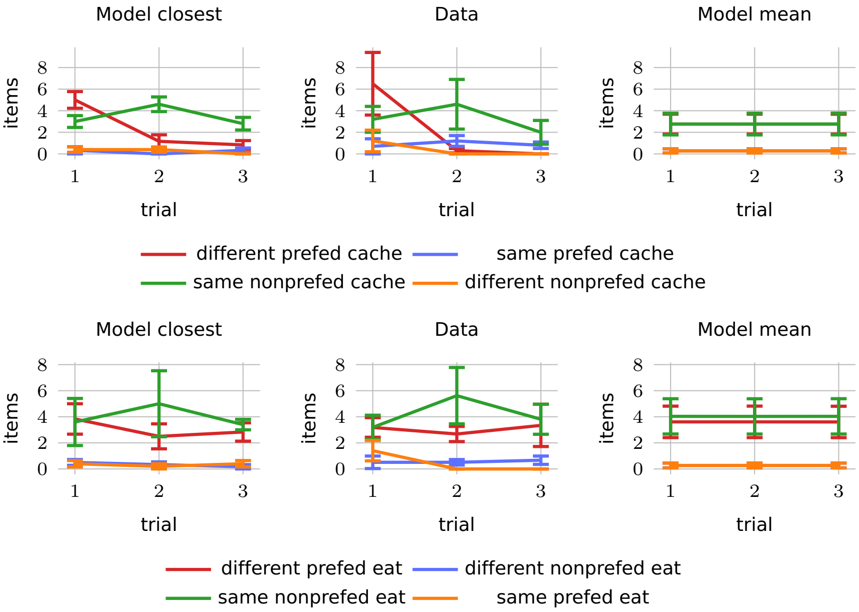

Figure S25: **Correia07 exp2: No-Plasticity-No-Memory Model**

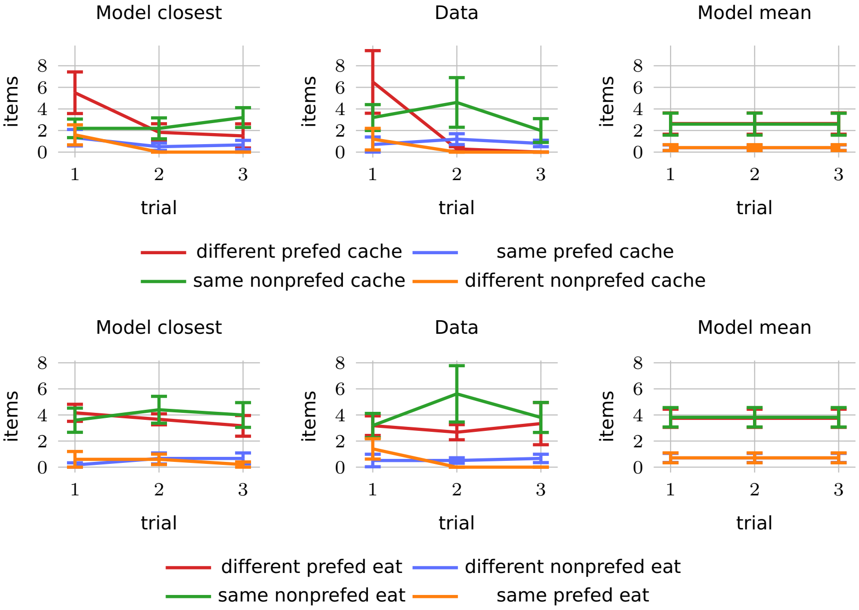

Figure S26: **Correia07 exp2: No-Plasticity Model**

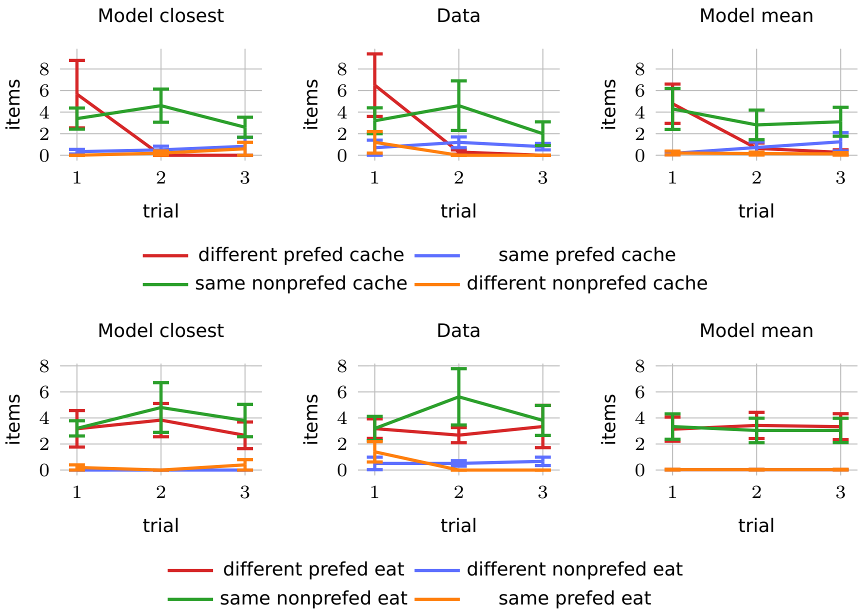

Figure S27: **Correia07 exp2: Plastic Caching Model**

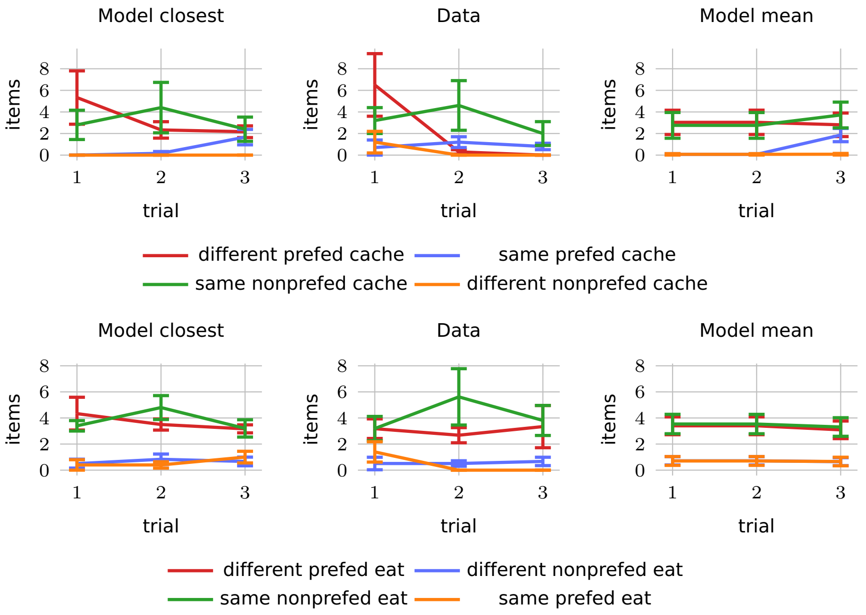

Figure S28: **Correia07 exp2: Planning-By-Replay Model**

2.1.5 deKort07 exp2

Published in [9].

Major Finding

Birds learn to reduce caching in sites where they always found their caches pilfered or the food items degraded, provided they had alternative caching locations.

Comments

The birds learned to reduce caching on one side of the cage despite trial-unique configuration of Lego Duplo blocks surrounding the ice cube tray, indicating that they generalized based on the side of the cage and not based on the local visual appearance of the caching site.

Most Important Tests

Experiment b tray × trial (tray & trial): $F(3, 42) = 4.39, p = 0.0089$

Experiment b, tray pilfer (trial): $F(3, 21) = 3.89, p = 0.023$

Experiment a, tray degrade (trial): $F(5, 45) = 2.46, p = 0.047$

Total Number of Statistics

10 p-values, 40 means or sems.

Figures

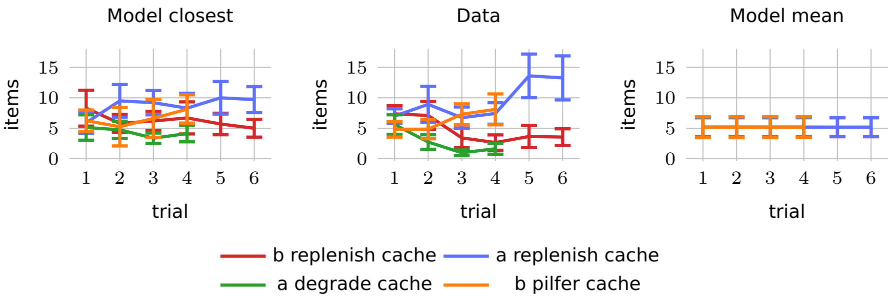

Figure S29: **deKort07 exp2: No-Plasticity-No-Memory-No-Motivational-Control Model**

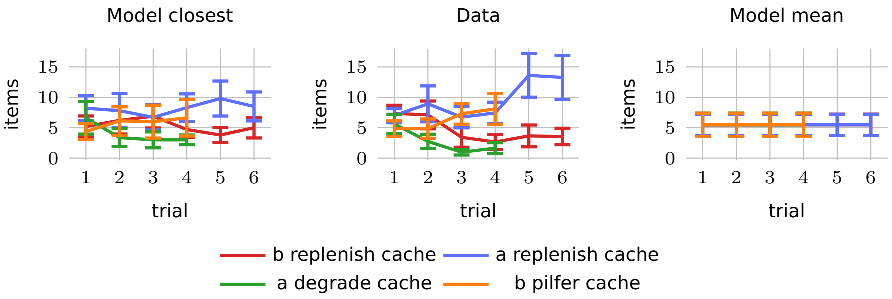

Figure S30: **deKort07 exp2: No-Plasticity-No-Memory Model**

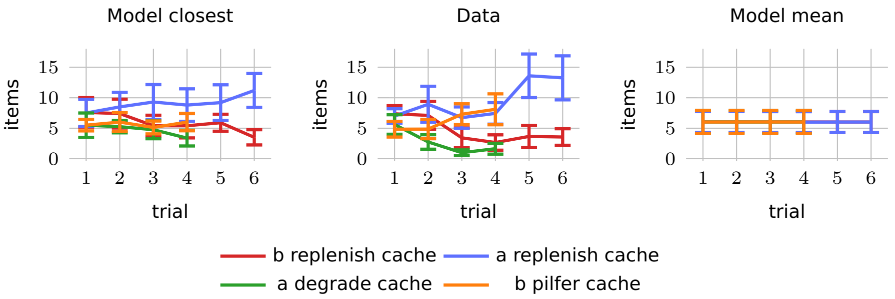

Figure S31: **deKort07 exp2: No-Plasticity Model**

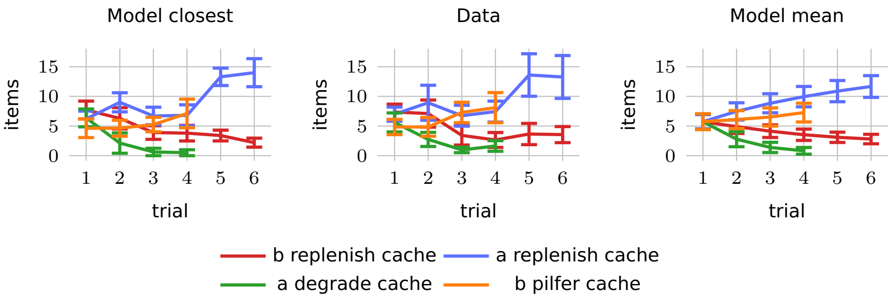

Figure S32: **deKort07 exp2: Plastic Caching Model**

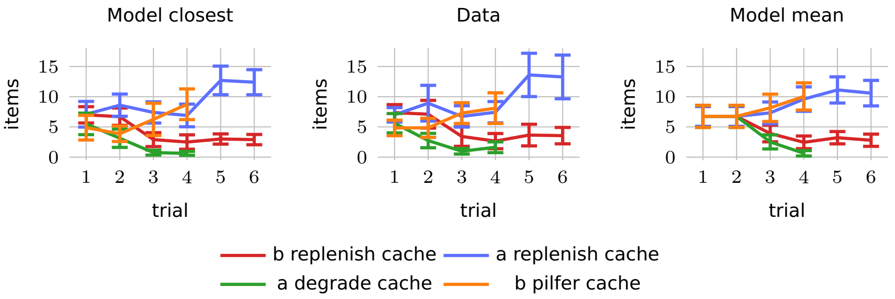

Figure S33: **deKort07 exp2: Planning-By-Replay Model**

2.1.6 deKort07 exp3

Published in [9].

Major Finding

Birds reduced caching in pilfered trays, even when the caching trays were available sequentially.

Most Important Tests

tray × trial (tray & trial): $F(9, 108) = 3.88, p = 0.00027$

Tray period2 (trial): $F(9, 54) = 3.18, p = 0.0037$

Tray period1 (trial): $F(9, 54) = 2.86, p = 0.0078$

Total Number of Statistics

3 p-values, 40 means or sems.

Figures

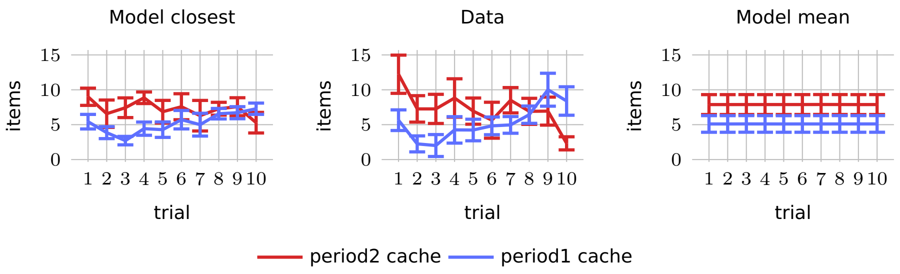

Figure S34: **deKort07 exp3: No-Plasticity-No-Memory-No-Motivational-Control Model**

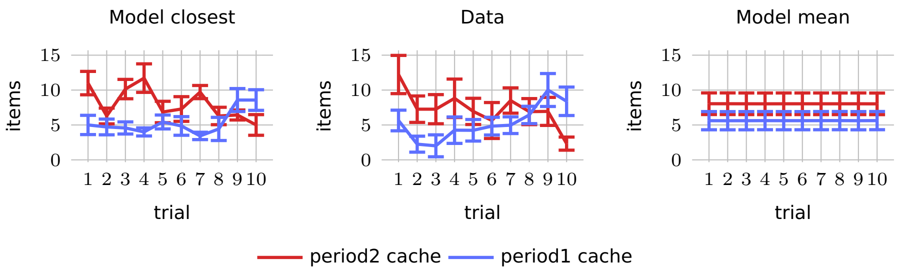

Figure S35: **deKort07 exp3: No-Plasticity-No-Memory Model**

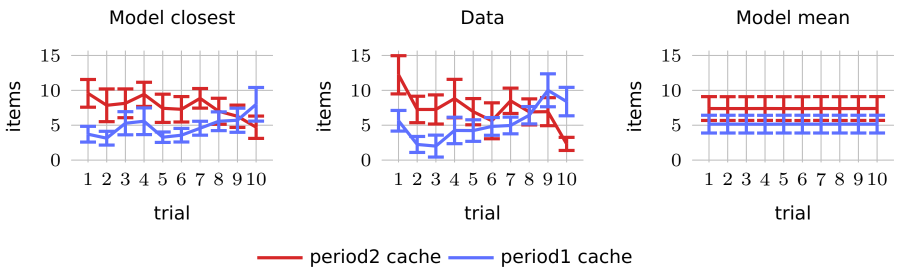

Figure S36: **deKort07 exp3: No-Plasticity Model**

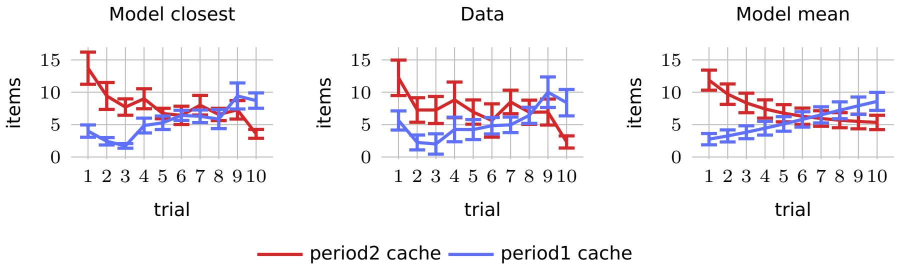

Figure S37: **deKort07 exp3: Plastic Caching Model**

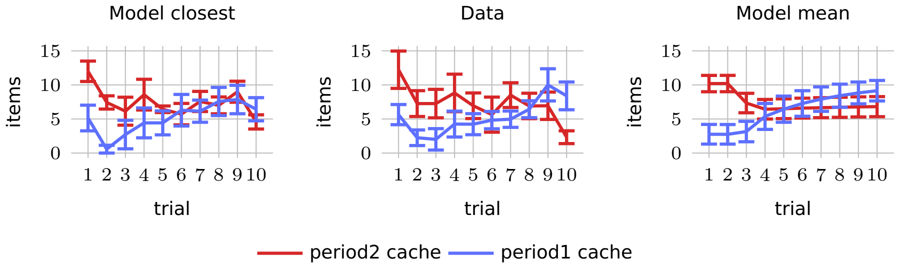

Figure S38: **deKort07 exp3: Planning-By-Replay Model**

2.1.7 deKort07 exp4

Published in [9].

Major Finding

Rather than caching in rewarded locations, the birds learned to avoid the unrewarded ones.

Most Important Tests

experimental group caches more in B than control (group): $F(1,6) = 9.26, p = 0.023$

Effect of tray on the worms cache (tray): $F(2,6) = 7.73, p = 0.022$

control group caches more in A than in B (tray): $F(1,6) = 8.03, p = 0.03$

Total Number of Statistics

11 p-values, 14 means or sems.

Figures

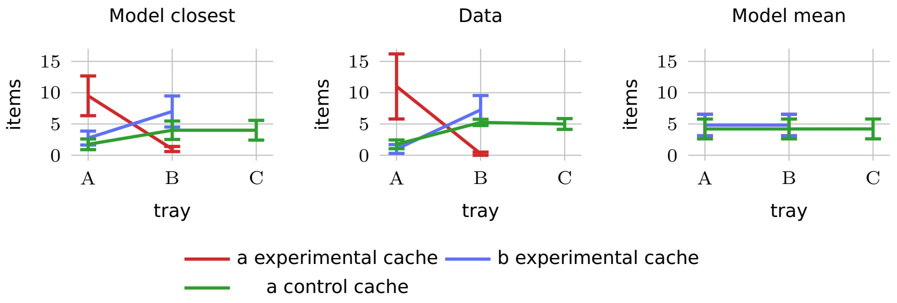

Figure S39: **deKort07 exp4: No-Plasticity-No-Memory-No-Motivational-Control Model**

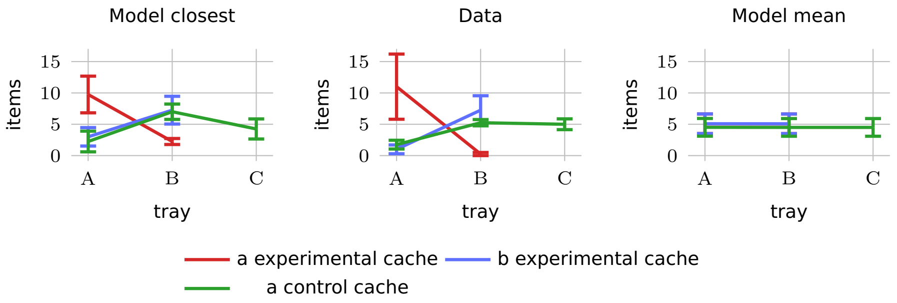

Figure S40: **deKort07 exp4: No-Plasticity-No-Memory Model**

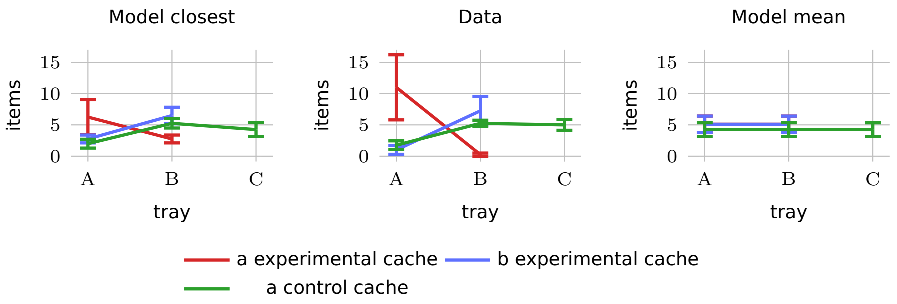

Figure S41: **deKort07 exp4: No-Plasticity Model**

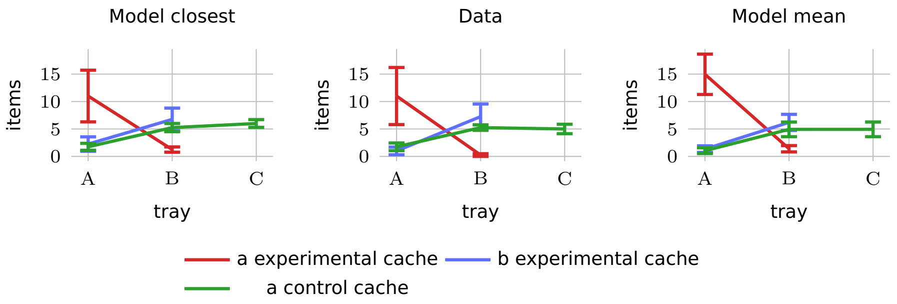

Figure S42: **deKort07 exp4: Plastic Caching Model**

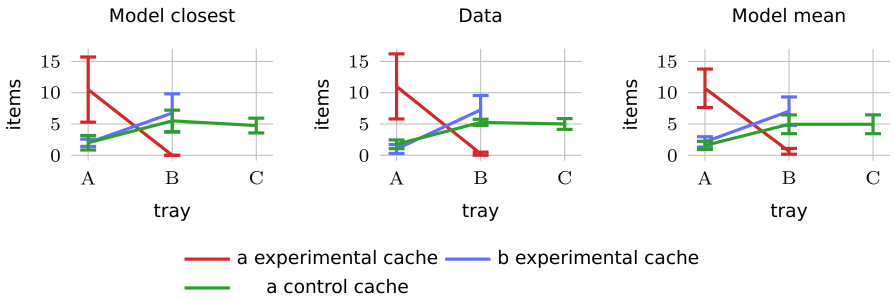

Figure S43: **deKort07 exp4: Planning-By-Replay Model**

2.1.8 Clayton05 exp1

Published in [8].

Major Finding

Birds reduce caching foodtypes that are always degraded at recovery, but keep caching when the foodtypes are fresh after some retention intervals.

Most Important Tests

Group partial degrade (foodtype): $F(1, 3) = 85.15, p = 0.0027$

Group consistent degrade (trial & foodtype): $F(5, 15) = 12.24, p = 7.4 \cdot 10^{-5}$

Group replenish (trial & foodtype): $F(5, 15) = 1.34, p = 0.3$

Total Number of Statistics

14 p-values, 36 means or sems.

Figures

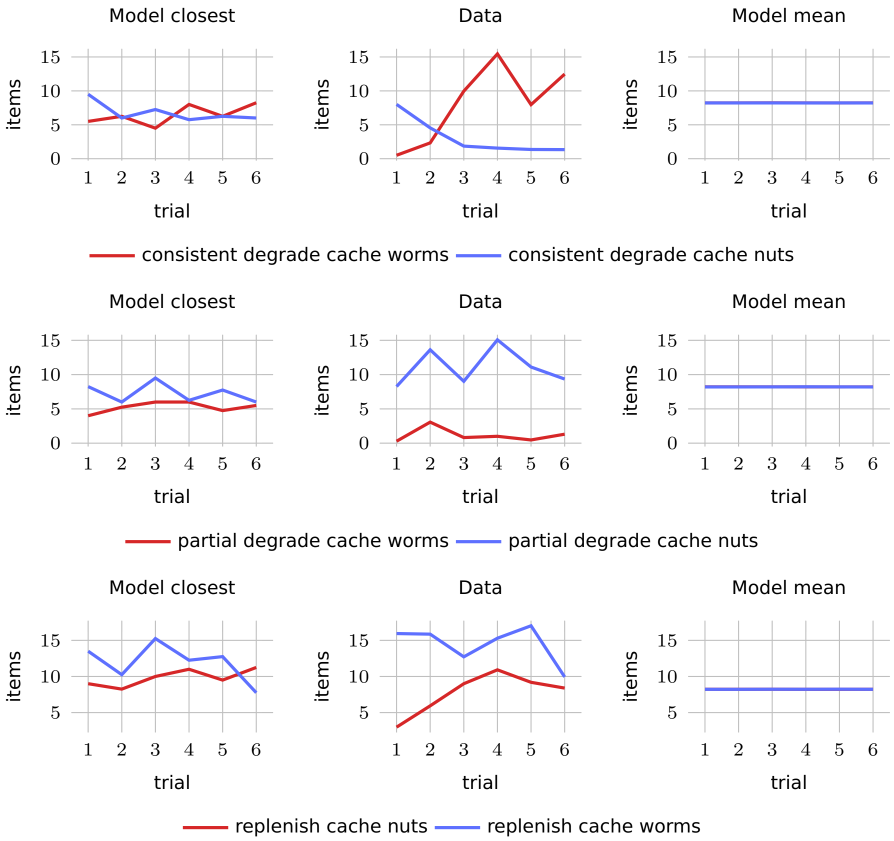

Figure S44: **Clayton05 exp1: No-Plasticity-No-Memory-No-Motivational-Control Model**

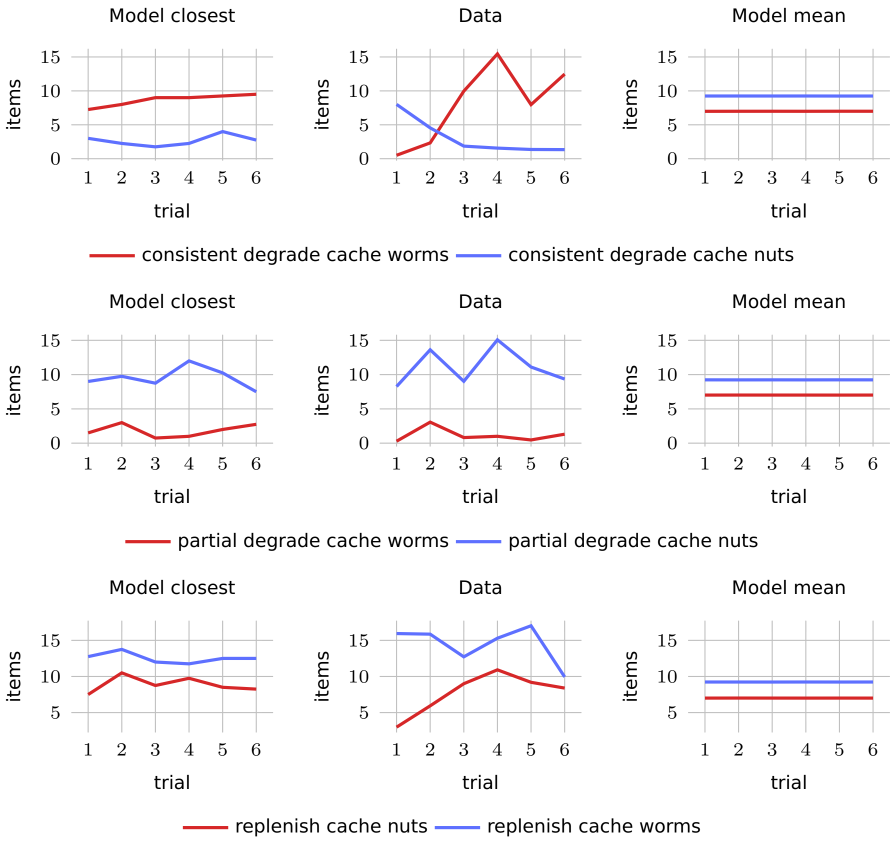

Figure S45: **Clayton05 exp1: No-Plasticity-No-Memory Model**

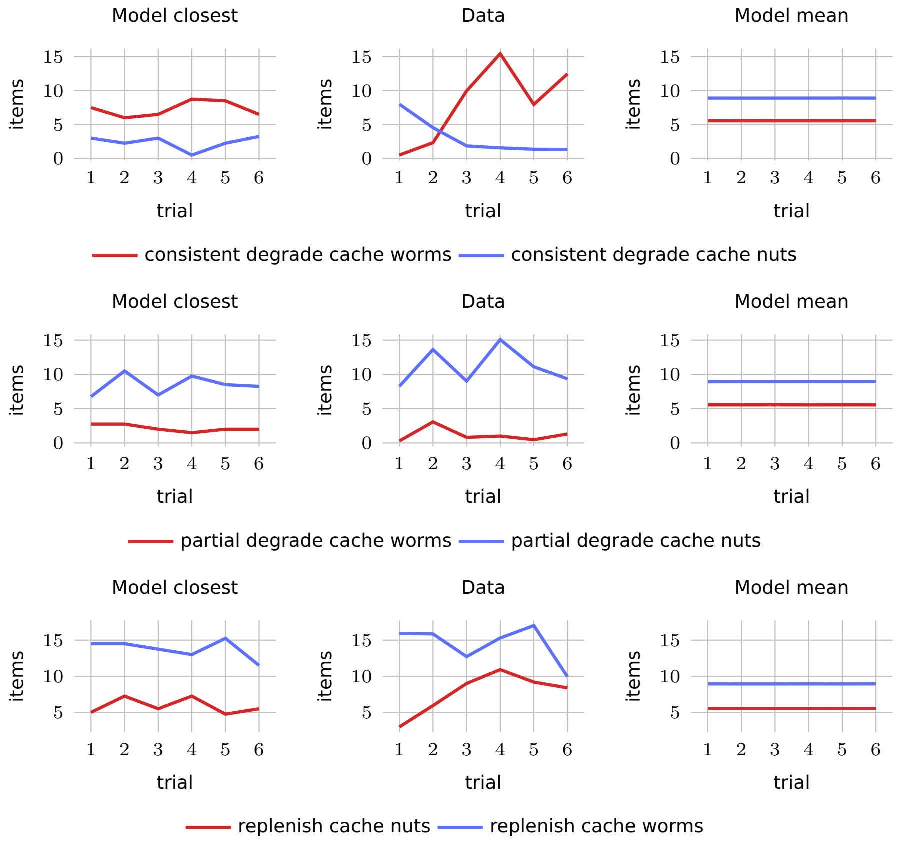

Figure S46: **Clayton05 exp1: No-Plasticity Model**

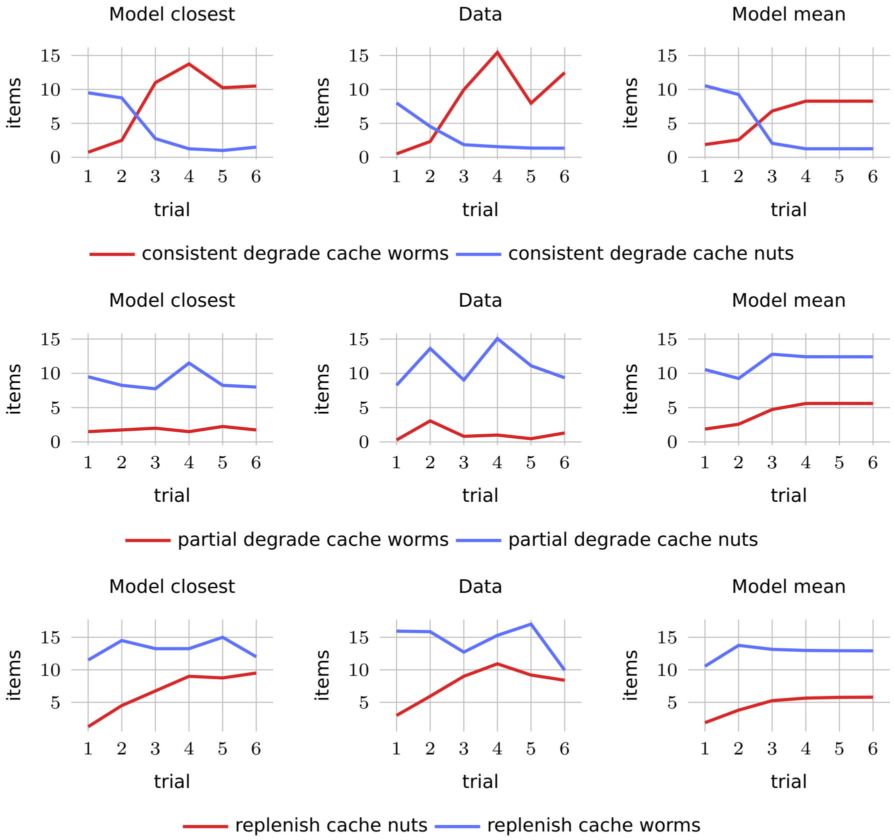

Figure S47: **Clayton05 exp1: Plastic Caching Model**

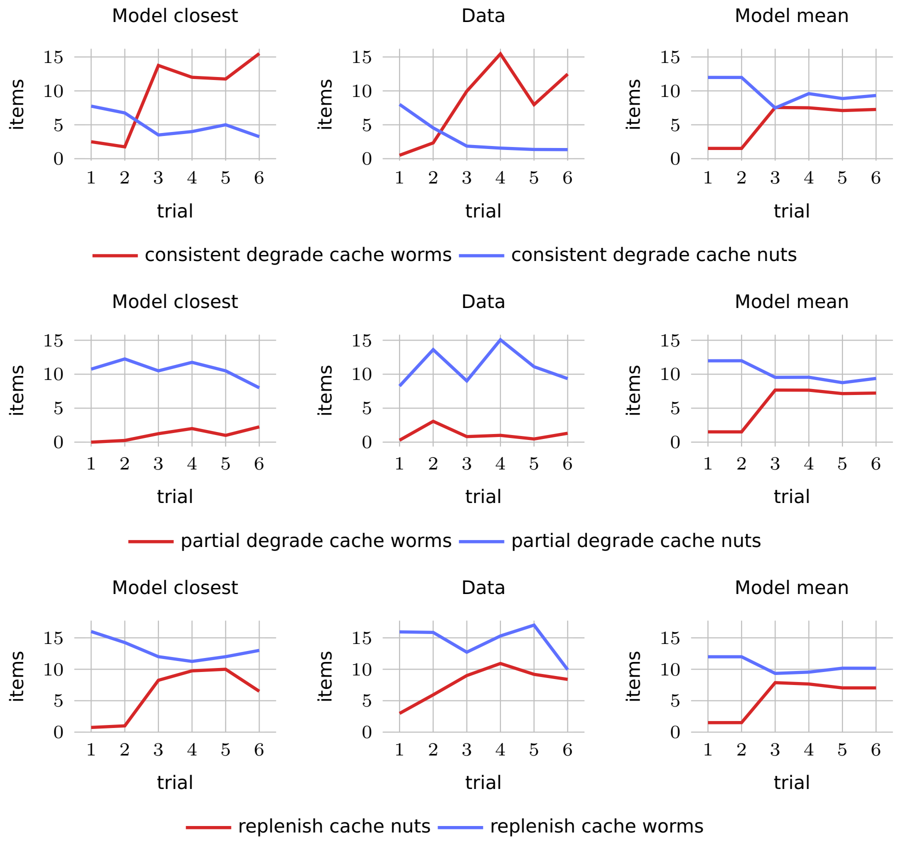

Figure S48: **Clayton05 exp1: Planning-By-Replay Model**

2.1.9 Clayton05 exp2

Published in [8].

Major Finding

Replication of experiment one showing additionally that consistent and partial pilfering has a similar effect on caching as degradation.

Comments

For quite a few statistical tests we were not able to reconstruct the reported degrees of freedom.

Most Important Tests

Change in caching worms replenish (trial): $F(7, 63) < 1.06, p = 0.41$

Change in caching worms consistent degrade (trial): $F(7, 63) = 4.39, p = 0.00051$

Change in caching worms partial degrade (trial): $F(7, 63) < 1.06, p = 0.41$

Total Number of Statistics

19 p-values, 80 means or sems.

Figures

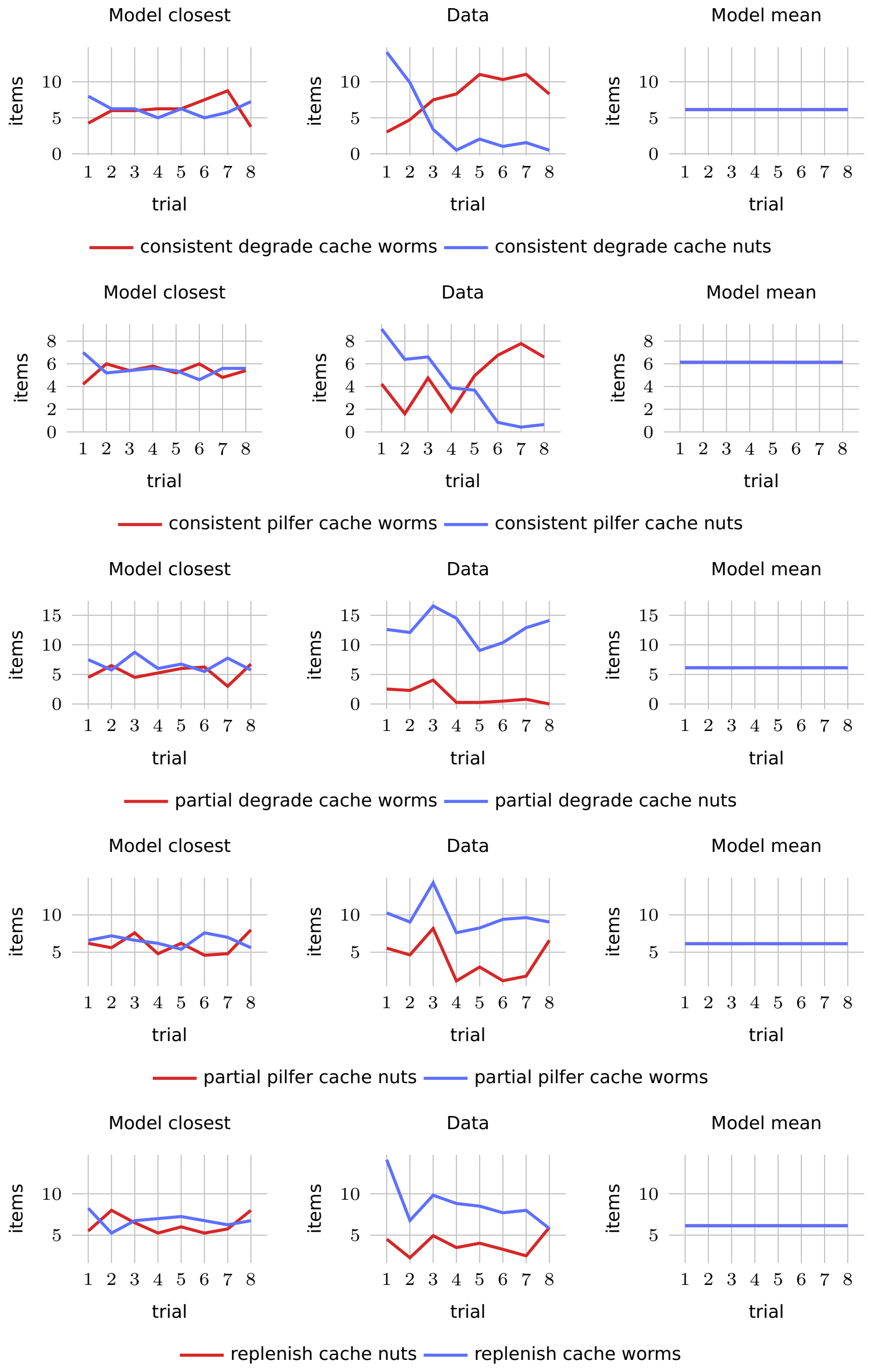

Figure S49: **Clayton05 exp2: No-Plasticity-No-Memory-No-Motivational-Control Model**

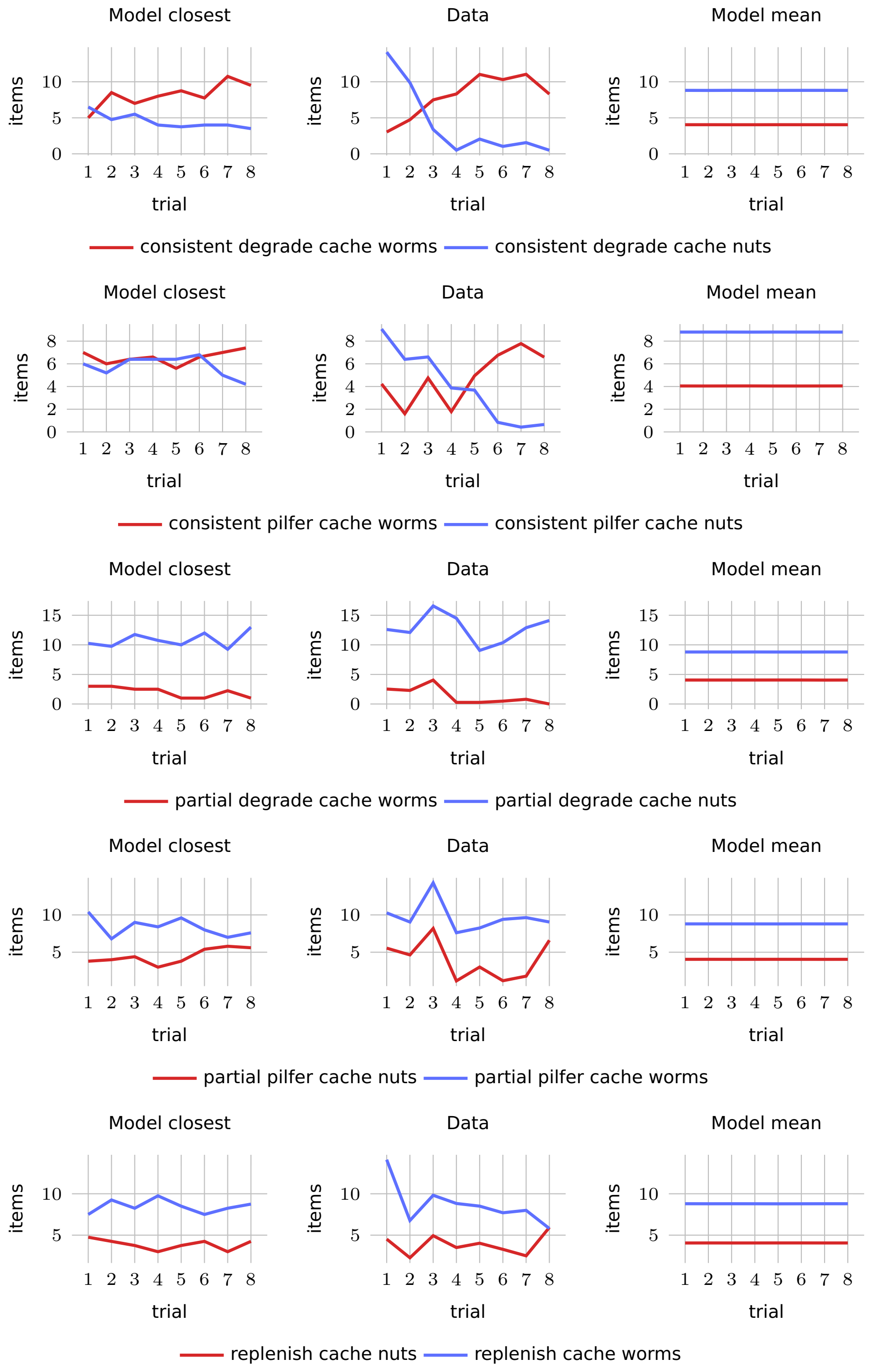

Figure S50: **Clayton05 exp2: No-Plasticity-No-Memory Model**

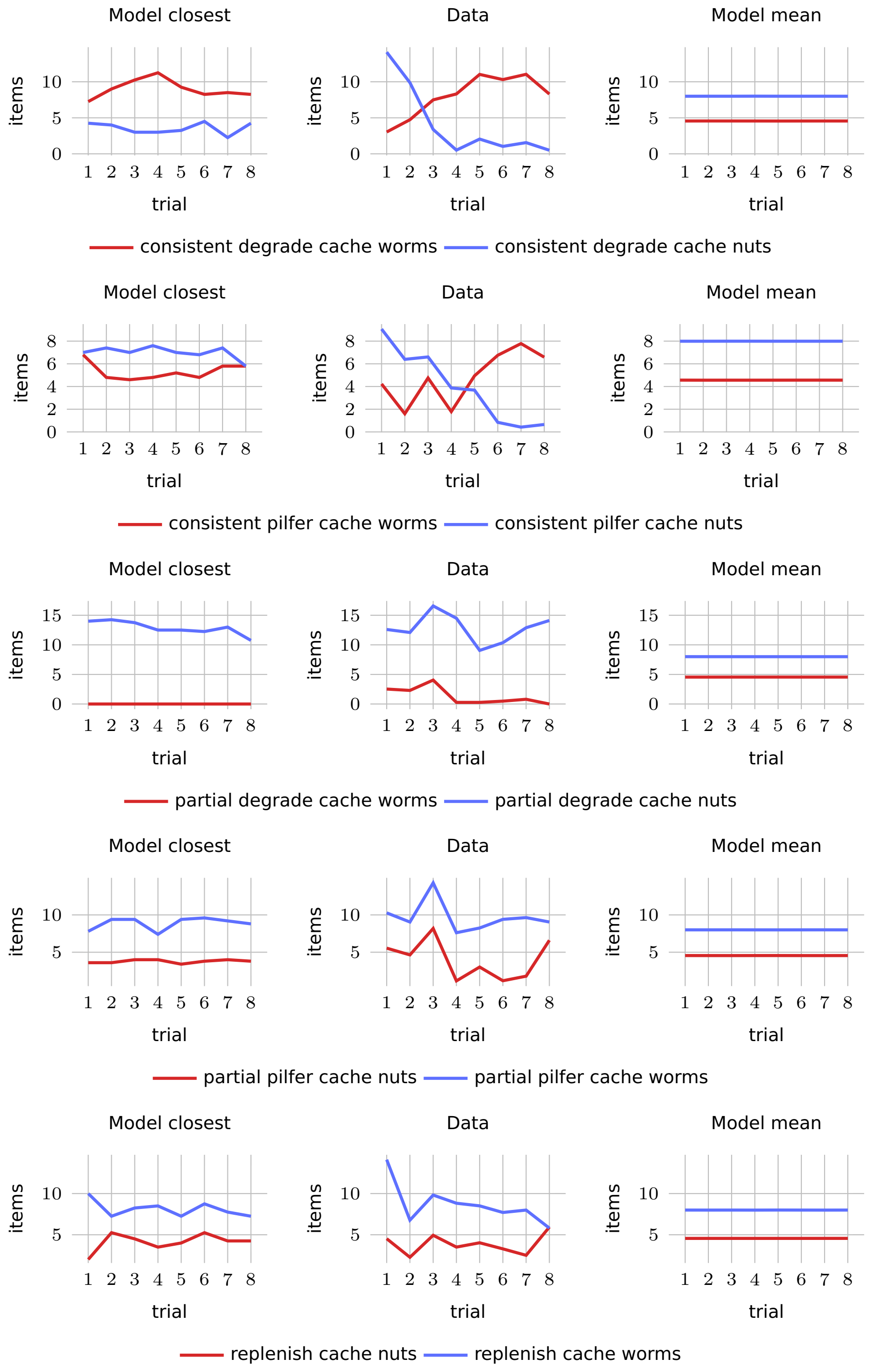

Figure S51: **Clayton05 exp2: No-Plasticity Model**

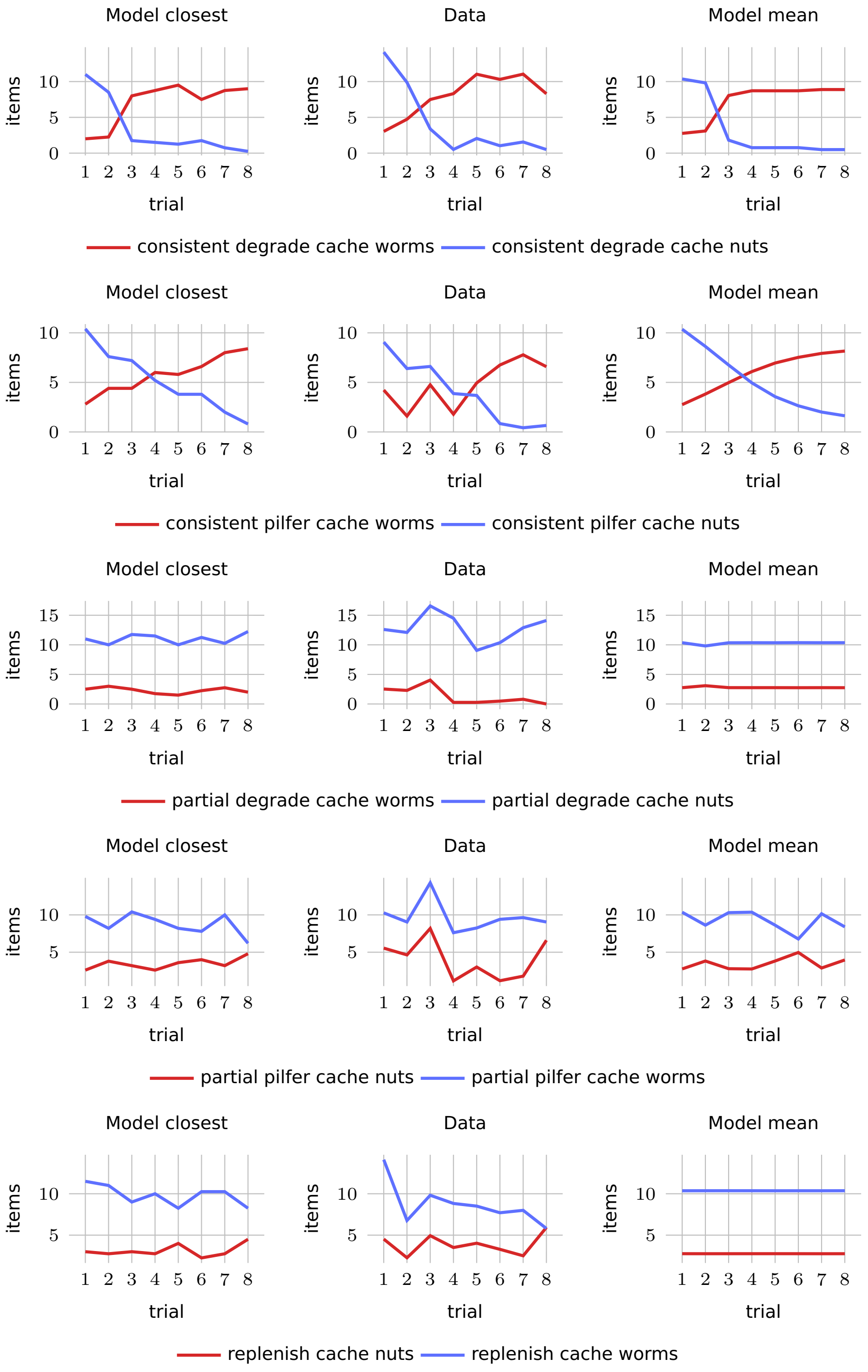

Figure S52: **Clayton05 exp2: Plastic Caching Model**

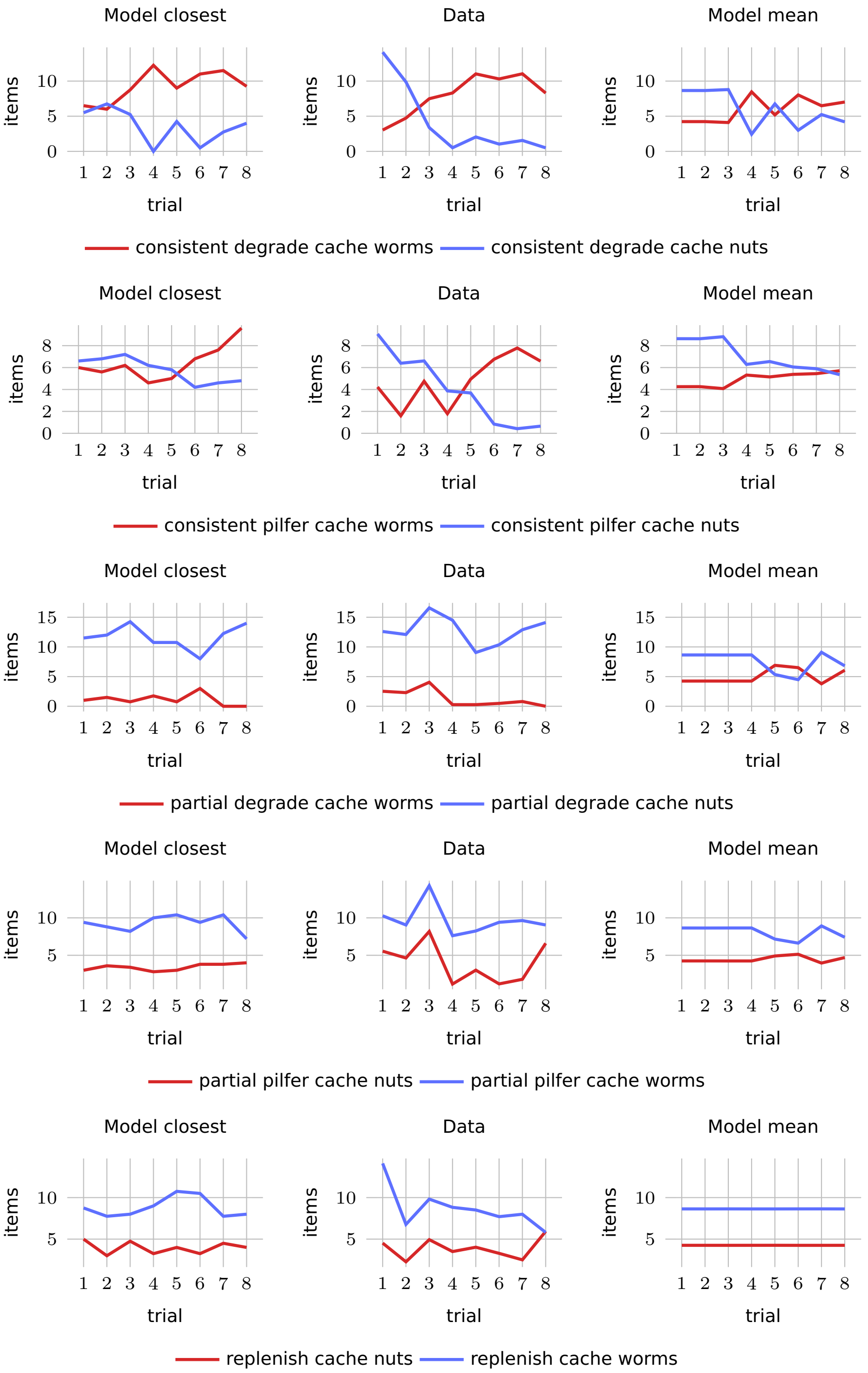

Figure S53: **Clayton05 exp2: Planning-By-Replay Model**

2.1.10 Clayton05 exp3

Published in [8].

Major Finding

Birds reduced caching of unfamiliar food types (Pineapple, Salami), if items of this type were consistently degraded at recovery.

Most Important Tests

partial degrade (foodtype & trial): $F(9, 54) < 1, p = 0.46$

overall (group & foodtype & trial): $F(9, 90) = 1.99, p = 0.049$

consistent degrade (foodtype & trial): $F(9, 36) = 2.75, p = 0.015$

Total Number of Statistics

20 p-values, 42 means or sems.

Figures

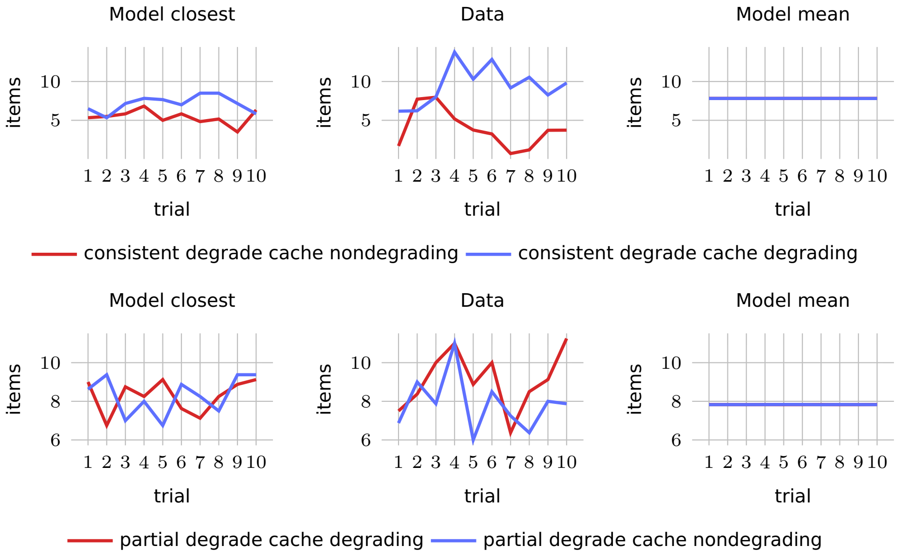

Figure S54: **Clayton05 exp3: No-Plasticity-No-Memory-No-Motivational-Control Model**

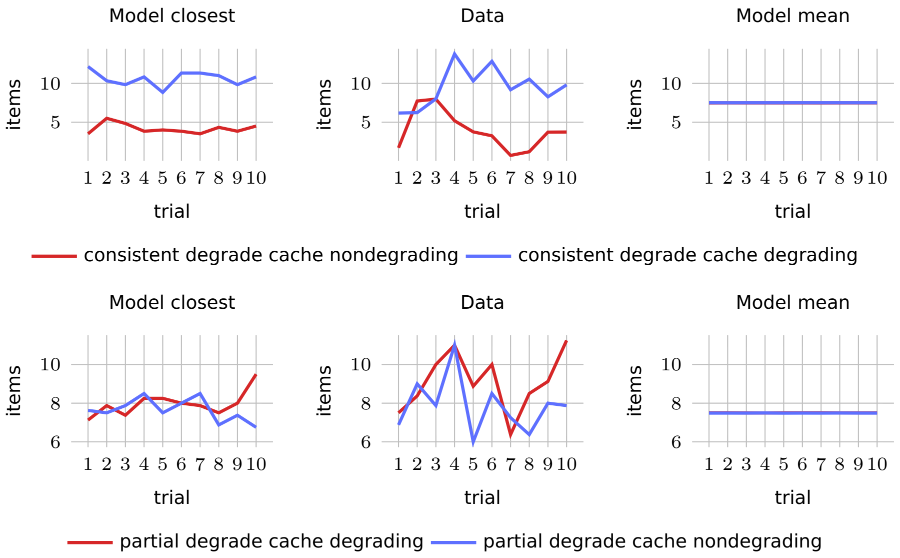

Figure S55: **Clayton05 exp3: No-Plasticity-No-Memory Model**

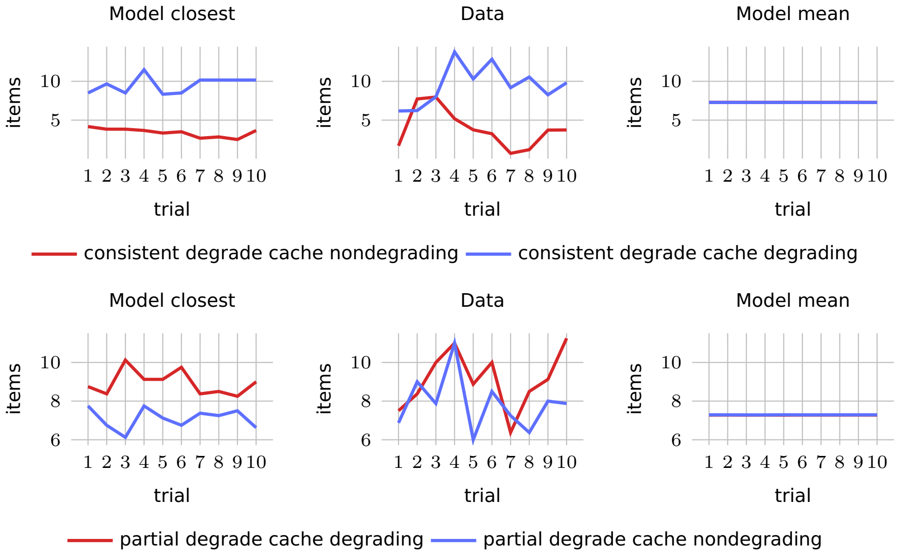

Figure S56: **Clayton05 exp3: No-Plasticity Model**

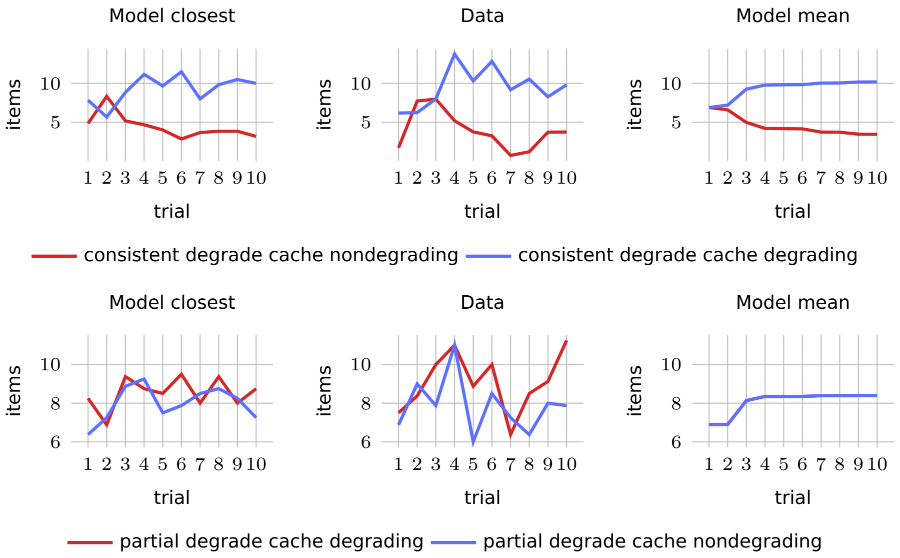

Figure S57: **Clayton05 exp3: Plastic Caching Model**

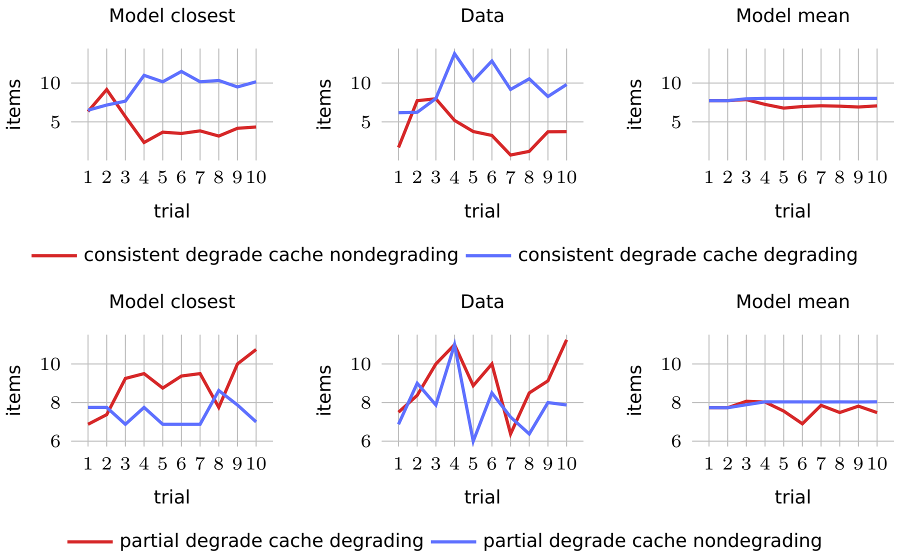

Figure S58: **Clayton05 exp3: Planning-By-Replay Model**

2.1.11 Clayton05 exp4

Published in [8].

Major Finding

The birds reduce caching items of the food type that was consistently degraded, even if the shortest retention interval was longer than two days.

Most Important Tests

overall (foodtype & trial): $F(3, 12) = 12.83, p = 0.00047$

trial 4 (foodtype): $F(1,4) = 18.42, p = 0.013$

worms (trial): $F(3,12) = 16.93, p = 0.00013$

Total Number of Statistics

5 p-values, 8 means or sems.

Figures

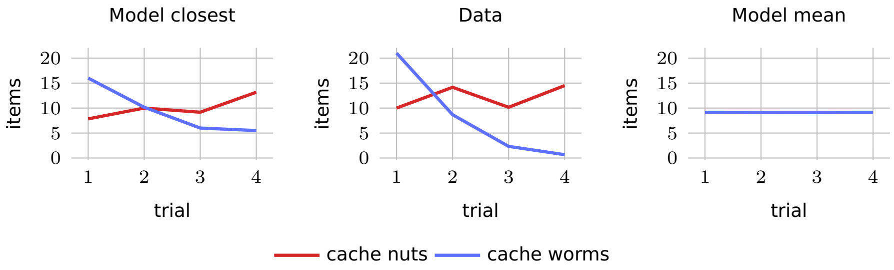

Figure S59: **Clayton05 exp4: No-Plasticity-No-Memory-No-Motivational-Control Model**

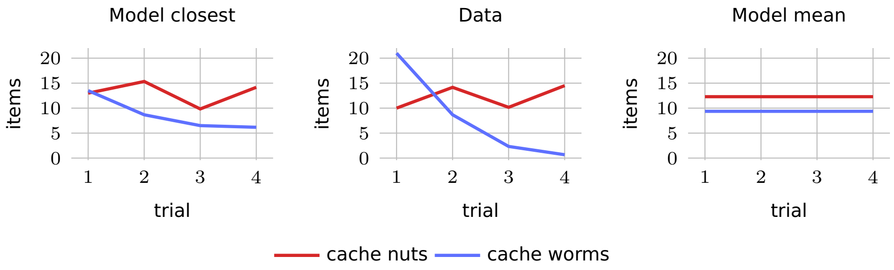

Figure S60: **Clayton05 exp4: No-Plasticity-No-Memory Model**

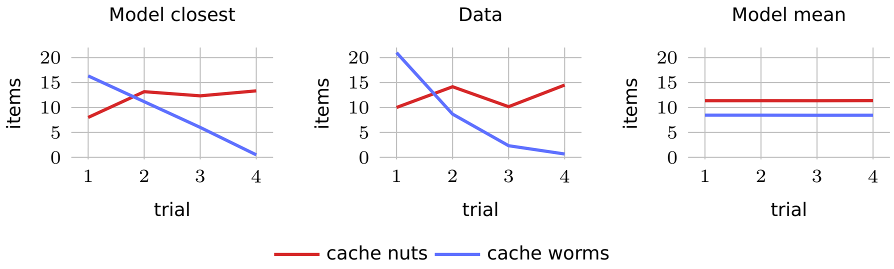

Figure S61: **Clayton05 exp4: No-Plasticity Model**

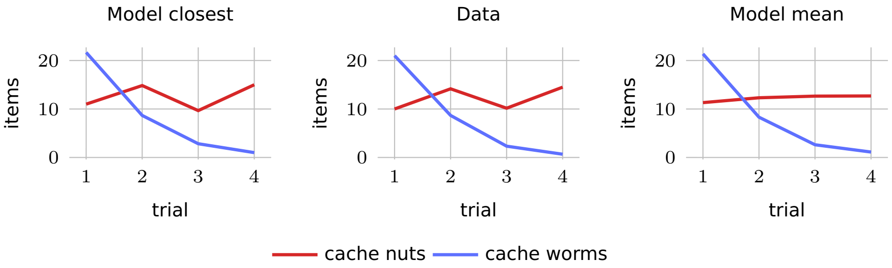

Figure S62: **Clayton05 exp4: Plastic Caching Model**

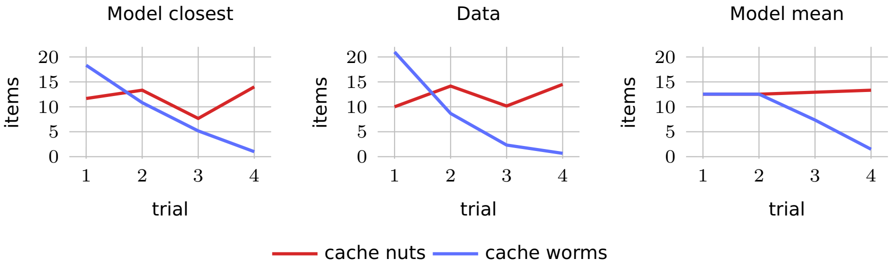

Figure S63: **Clayton05 exp4: Planning-By-Replay Model**

2.1.12 Clayton99A exp1

Published in [3].

Major Finding

Birds inspect preferentially caching sites where the previously cached food is of different type than the one they had been satied on prior to recovery.

Most Important Tests

172h overall inspect (tray): $F(1, 8) = 5.73, p = 0.044$

4h overall cache (tray): $F(1, 6) < 5.17, p = 0.064$

4h overall inspect (tray): $F(1, 6) = 11.32, p = 0.015$

Total Number of Statistics

44 p-values, 16 means or sems.

Figures

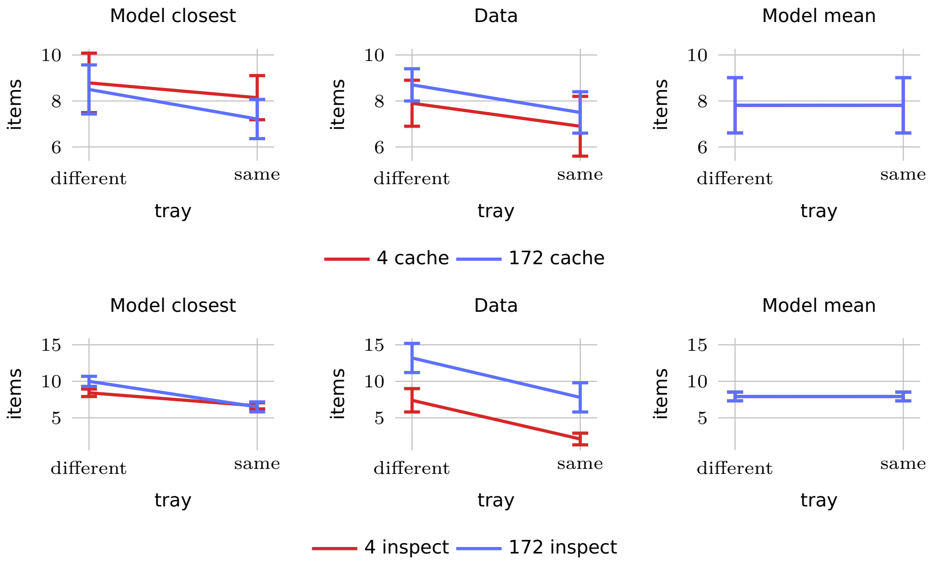

Figure S64: **Clayton99A exp1: No-Plasticity-No-Memory-No-Motivational-Control Model**

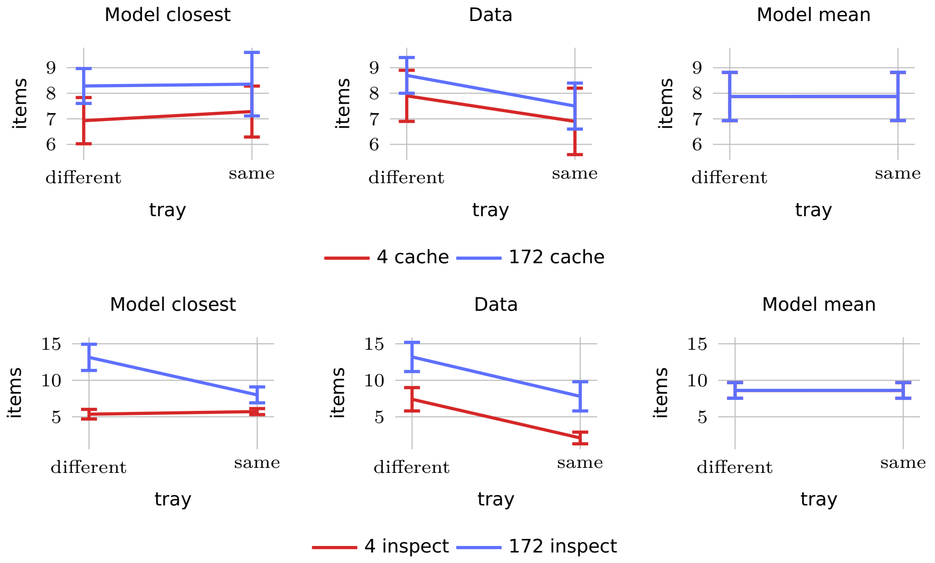

Figure S65: **Clayton99A exp1: No-Plasticity-No-Memory Model**

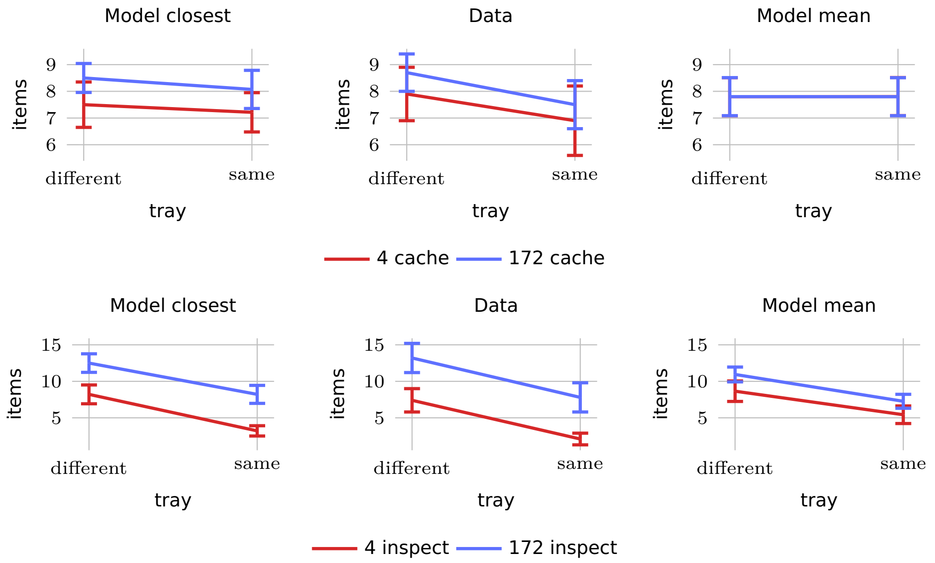

Figure S66: **Clayton99A exp1: No-Plasticity Model**

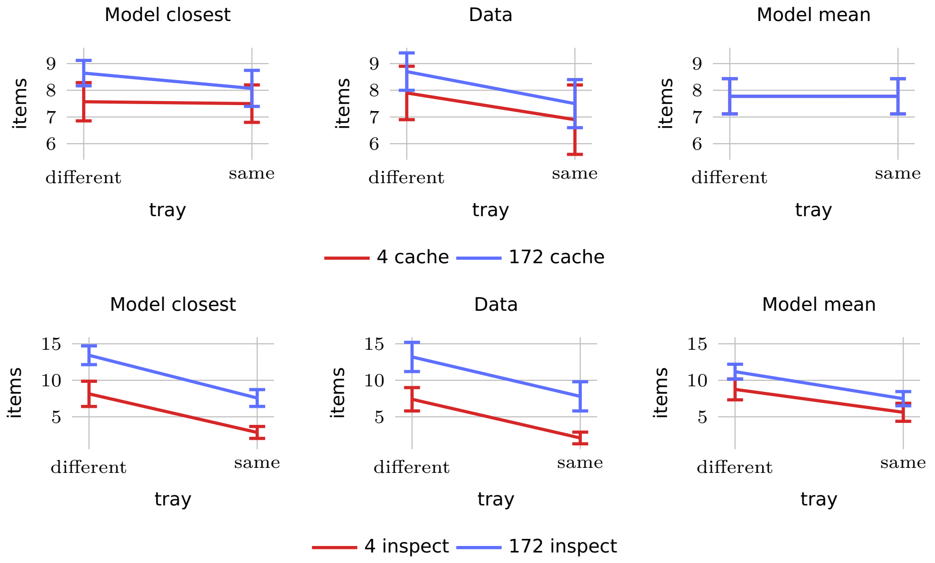

Figure S67: **Clayton99A exp1: Plastic Caching Model**

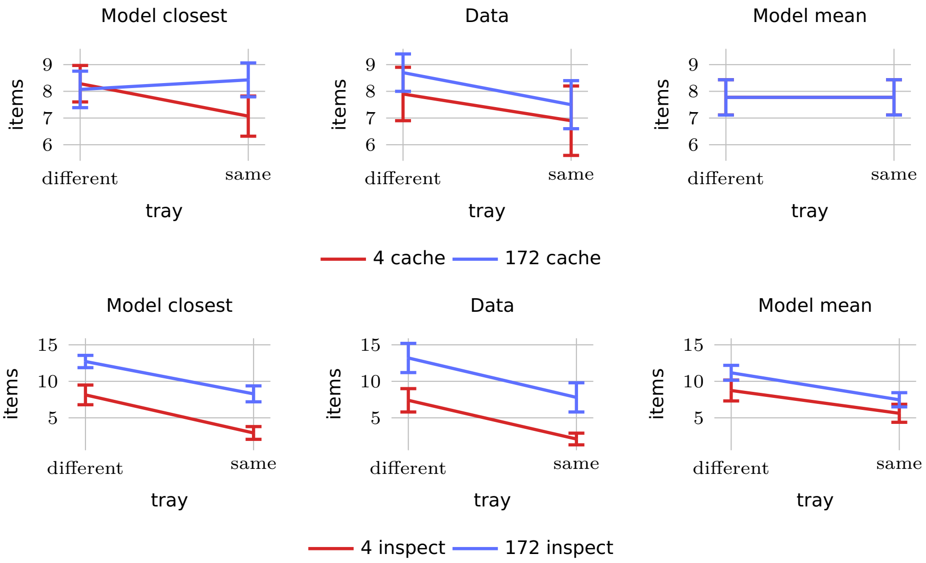

Figure S68: **Clayton99A exp1: Planning-By-Replay Model**

2.1.13 Clayton99A exp2

Published in [3].

Major Finding

Birds direct their searches to sites from which they did not yet recover the food items and contain food that is different from what they had just been prefed on.

Most Important Tests

inspect (tray): $F(1,10) = 21.59, p = 0.00091$

inspect (side): $F(1,10) = 74.39, p = 6.1 \cdot 10^{-6}$

inspect (tray & side): $F(1,10) = 20.27, p = 0.0011$

Total Number of Statistics

15 p-values, 8 means or sems.

Figures

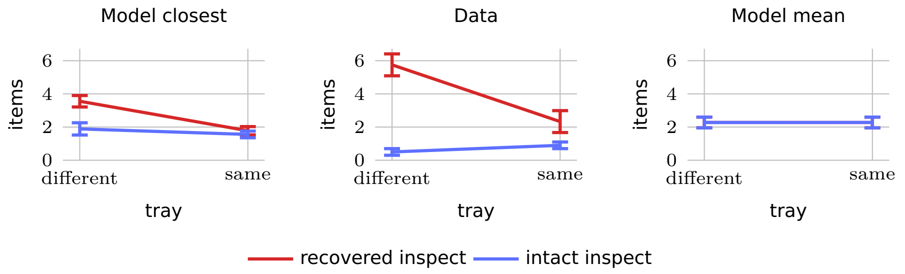

Figure S69: **Clayton99A exp2: No-Plasticity-No-Memory-No-Motivational-Control Model**

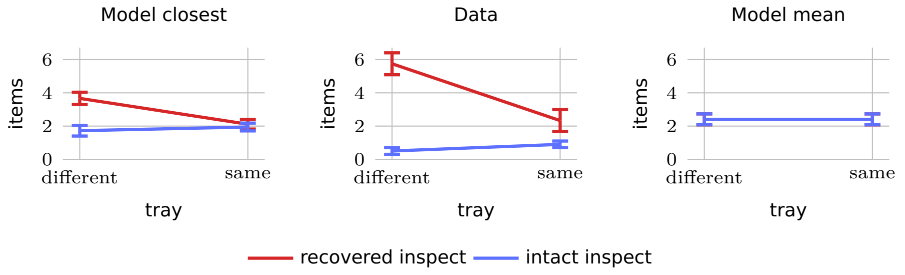

Figure S70: **Clayton99A exp2: No-Plasticity-No-Memory Model**

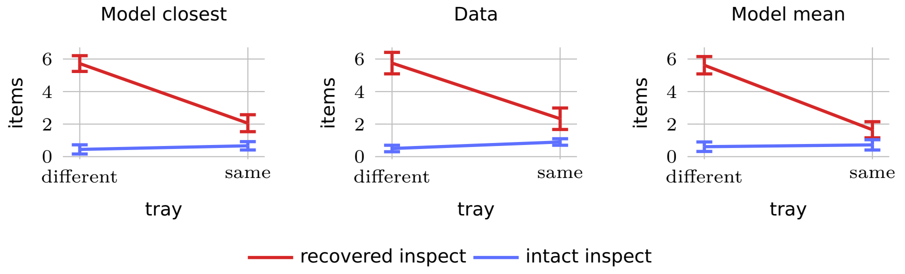

Figure S71: **Clayton99A exp2: No-Plasticity Model**

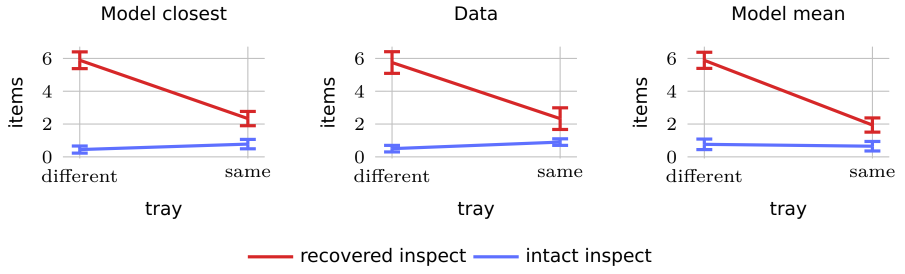

Figure S72: **Clayton99A exp2: Plastic Caching Model**

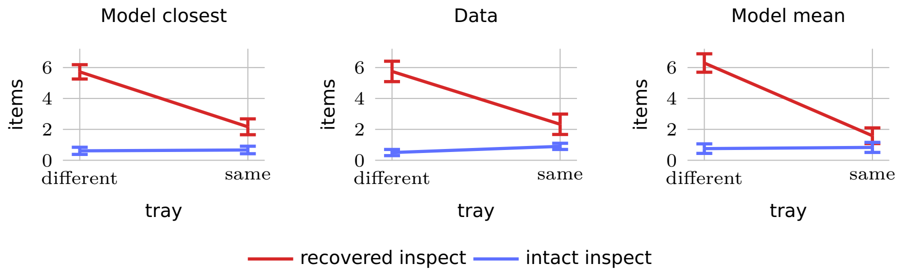

Figure S73: **Clayton99A exp2: Planning-By-Replay Model**

2.1.14 Clayton99B exp1

Published in [4].

Major Finding

The birds learned that their preferred food perished in the length of time since caching whereas their less preferred food did not: after some training trials, birds in the degrade group searched more at sites where they had previously cached their preferred food than at sites where they had previously cached their less preferred food after a retention interval of 4 hours and vice versa after a retention interval of 128 hours.

Comments

Birds in the degrade group cached progressively more wax worms than peanuts during training; a pattern not seen in Clayton05 experiment 1 where they continued to cache at roughly the same level.

Most Important Tests

test 4 (foodtype): $F(1, 17) = 18.21, p = 0.00052$

test 124 (group & foodtype): $F(2, 17) = 20.02, p = 3.4 \cdot 10^{-5}$

test (group & RI & foodtype): $F(2, 14) = 6.71, p = 0.009$

Total Number of Statistics

28 p-values, 184 means or sems.

Figures

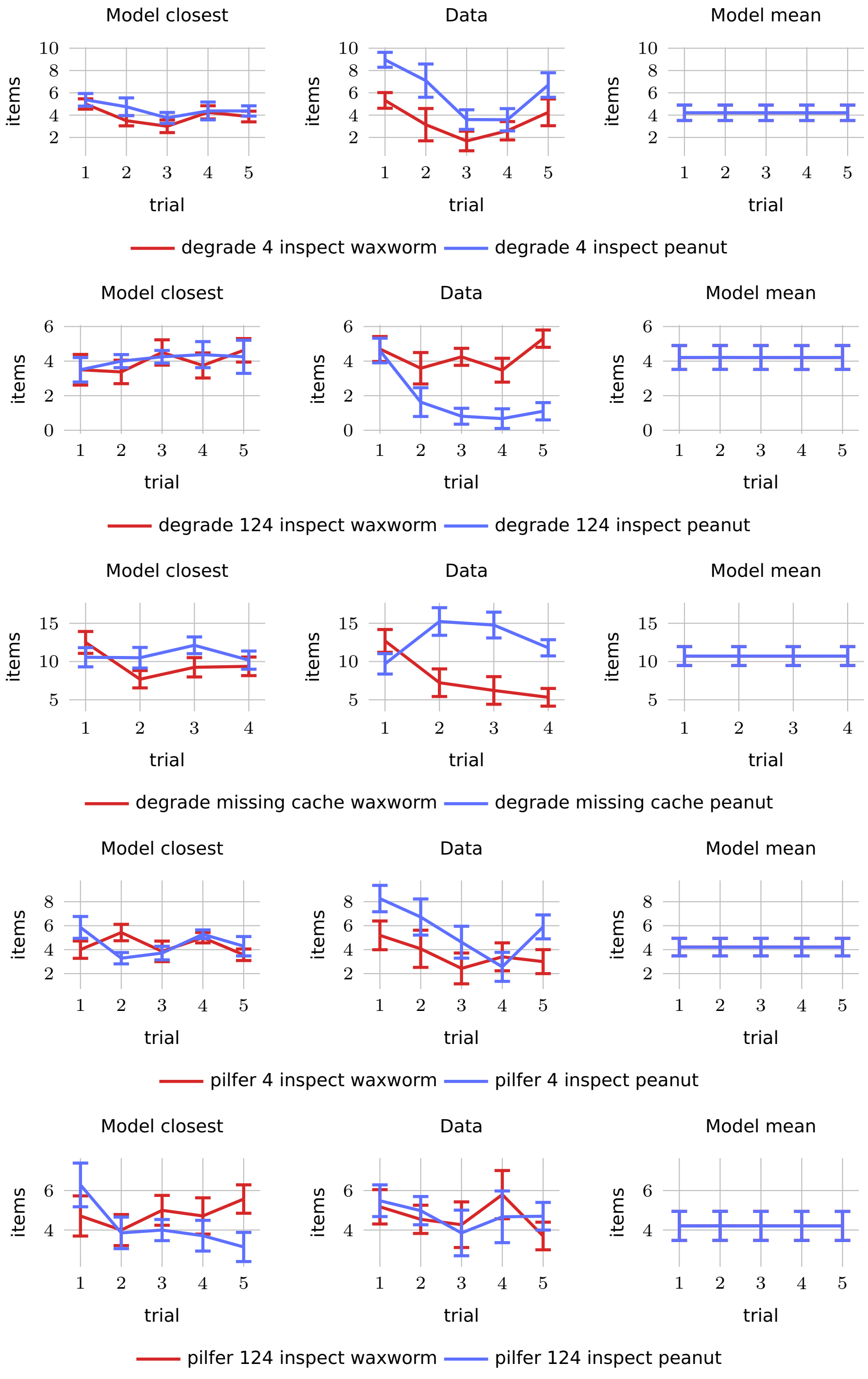

Figure S74: **Clayton99B exp1: No-Plasticity-No-Memory-No-Motivational-Control Model (part1)**

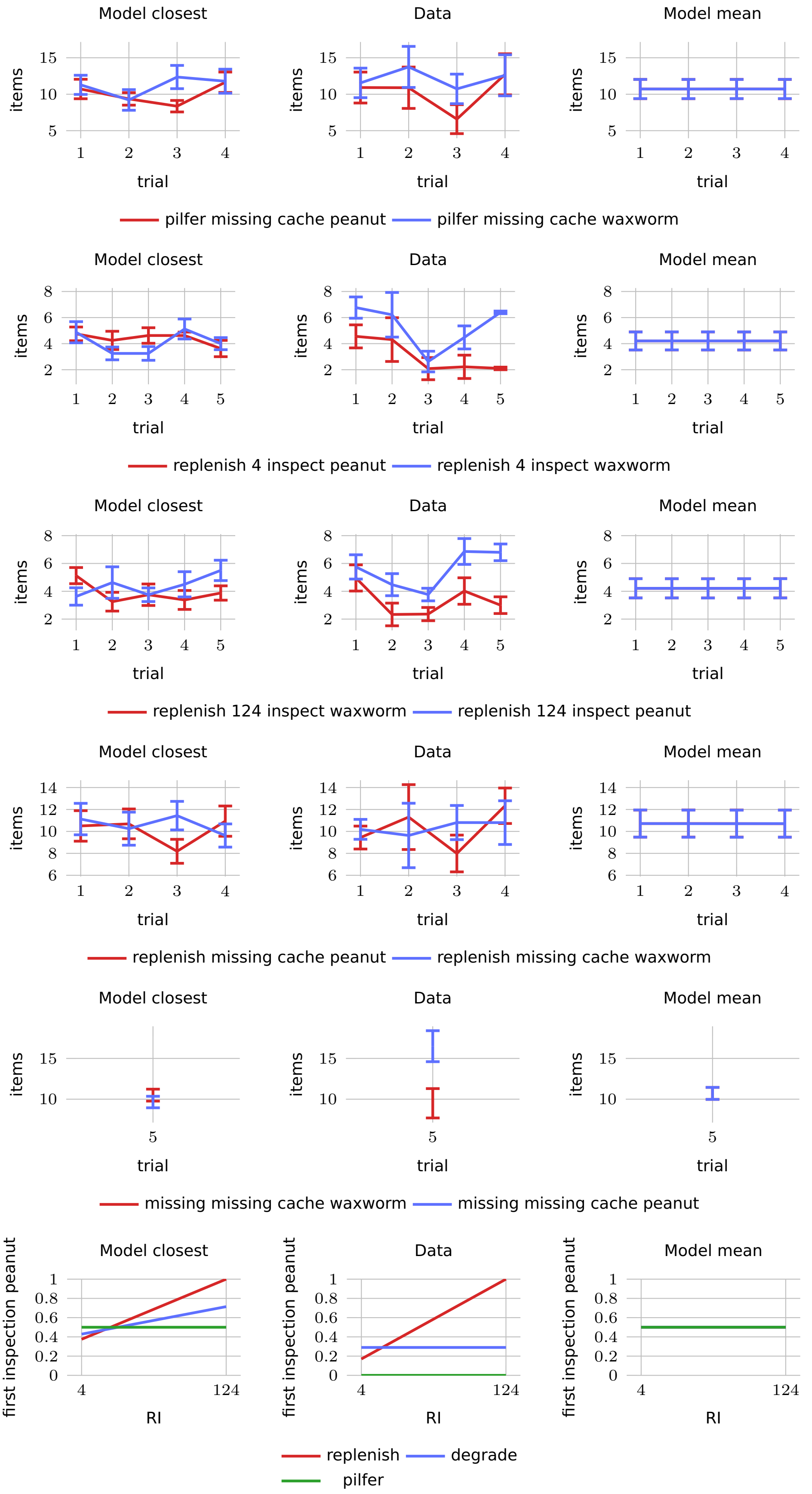

Figure S75: **Clayton99B exp1: No-Plasticity-No-Memory-No-Motivational-Control Model (part2)**

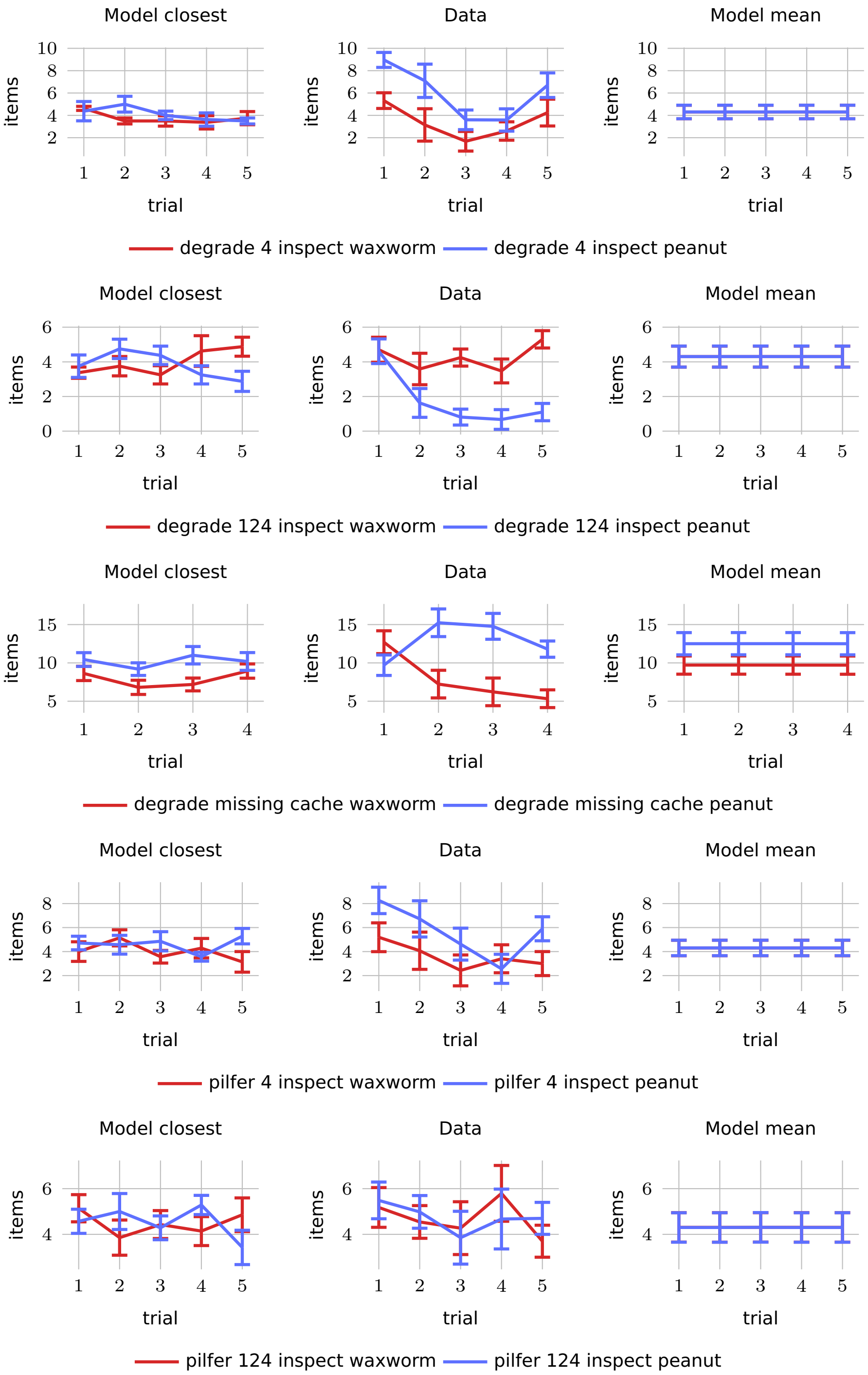

Figure S76: **Clayton99B exp1: No-Plasticity-No-Memory Model (part1)**

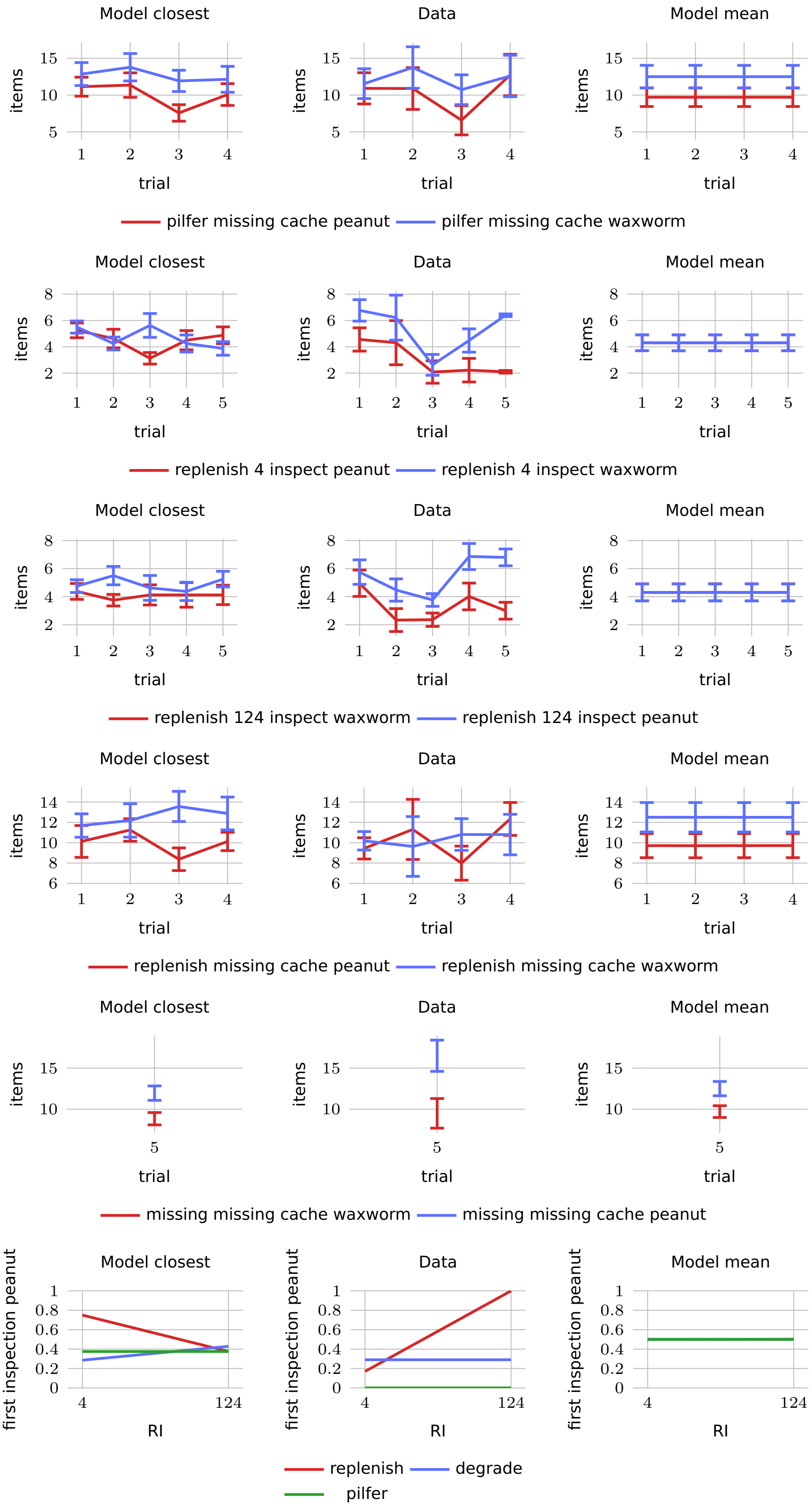

Figure S77: **Clayton99B exp1: No-Plasticity-No-Memory Model (part2)**

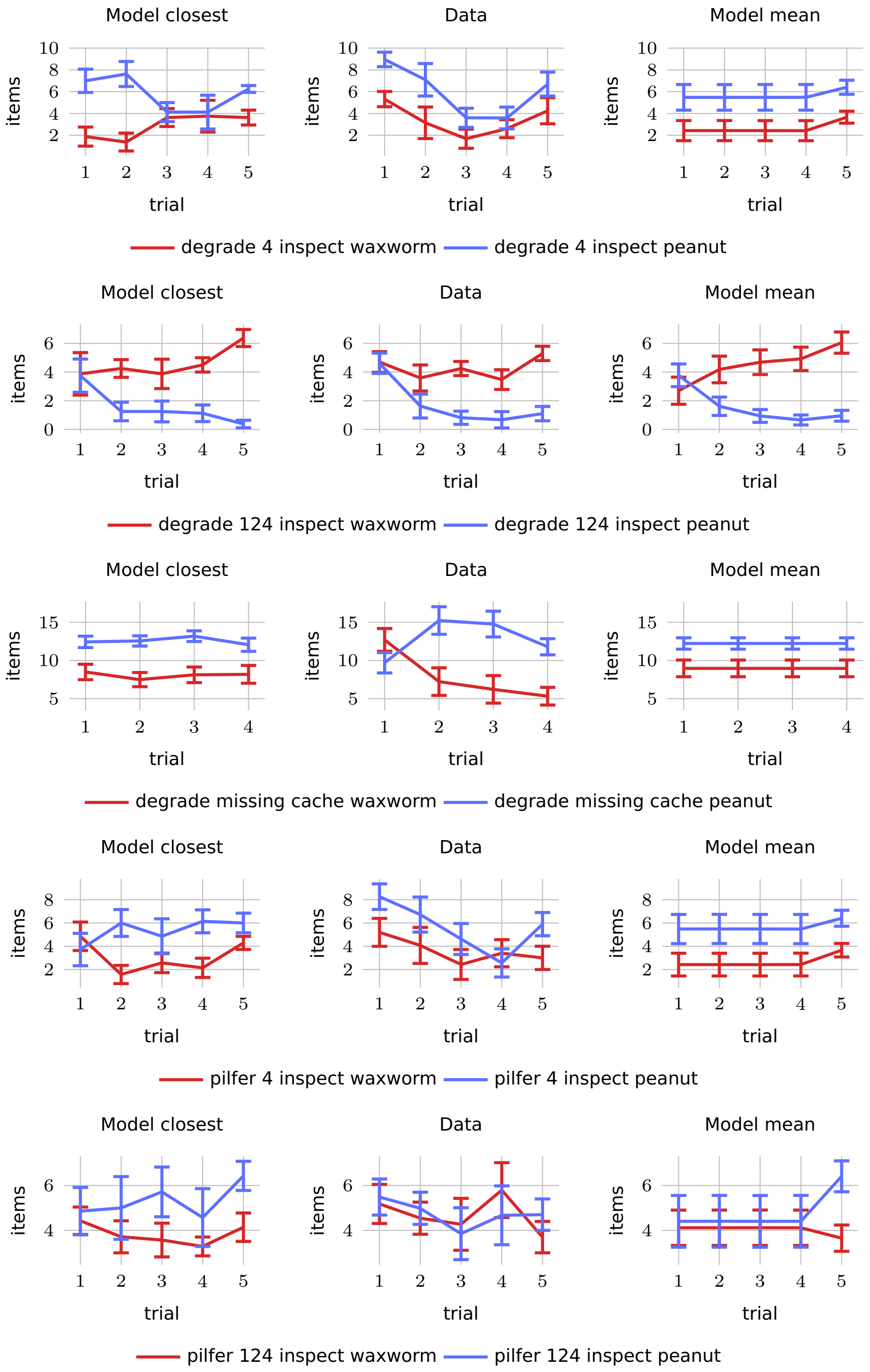

Figure S78: **Clayton99B exp1: No-Plasticity Model (part1)**

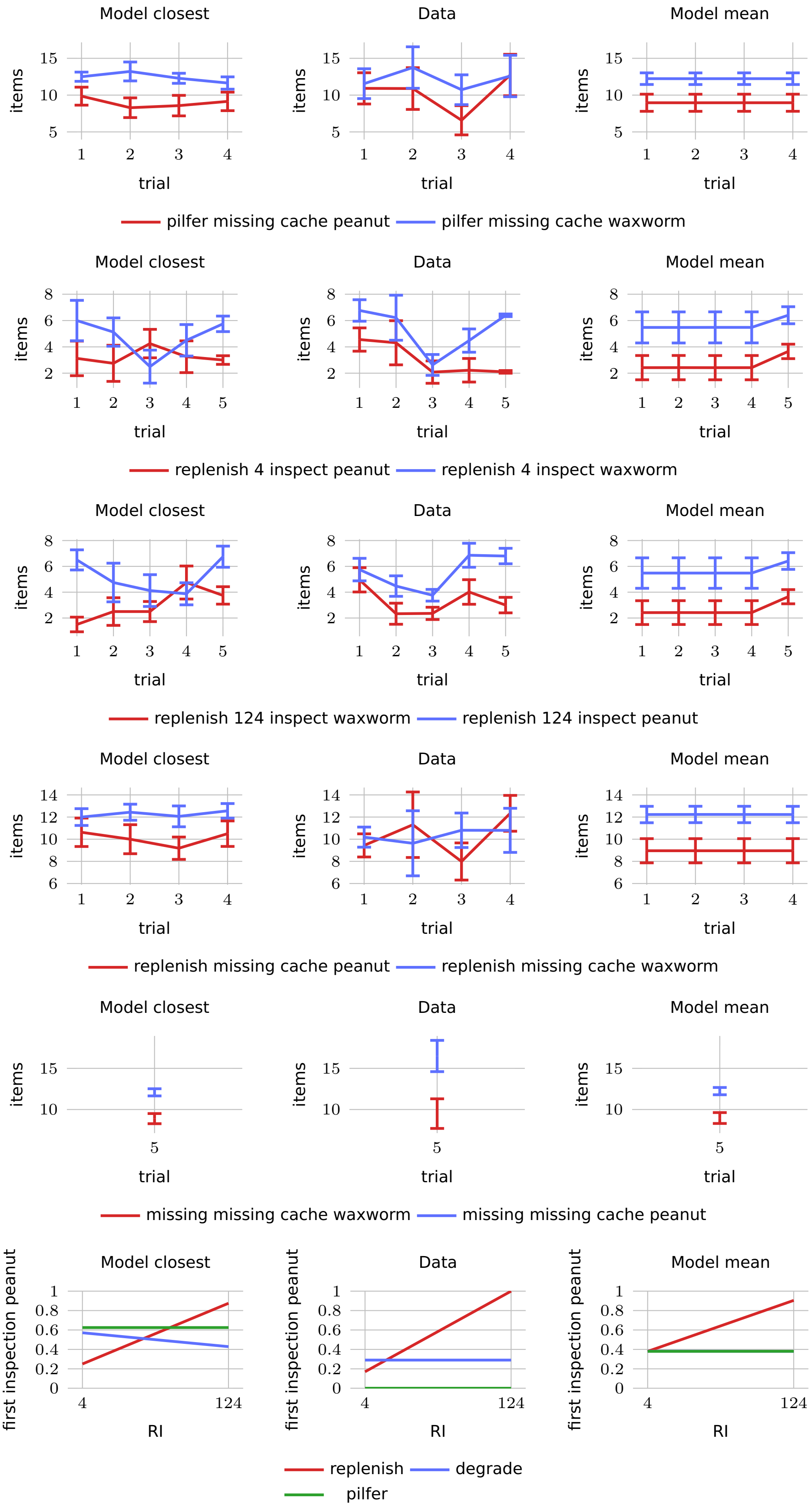

Figure S79: **Clayton99B exp1: No-Plasticity Model (part2)**

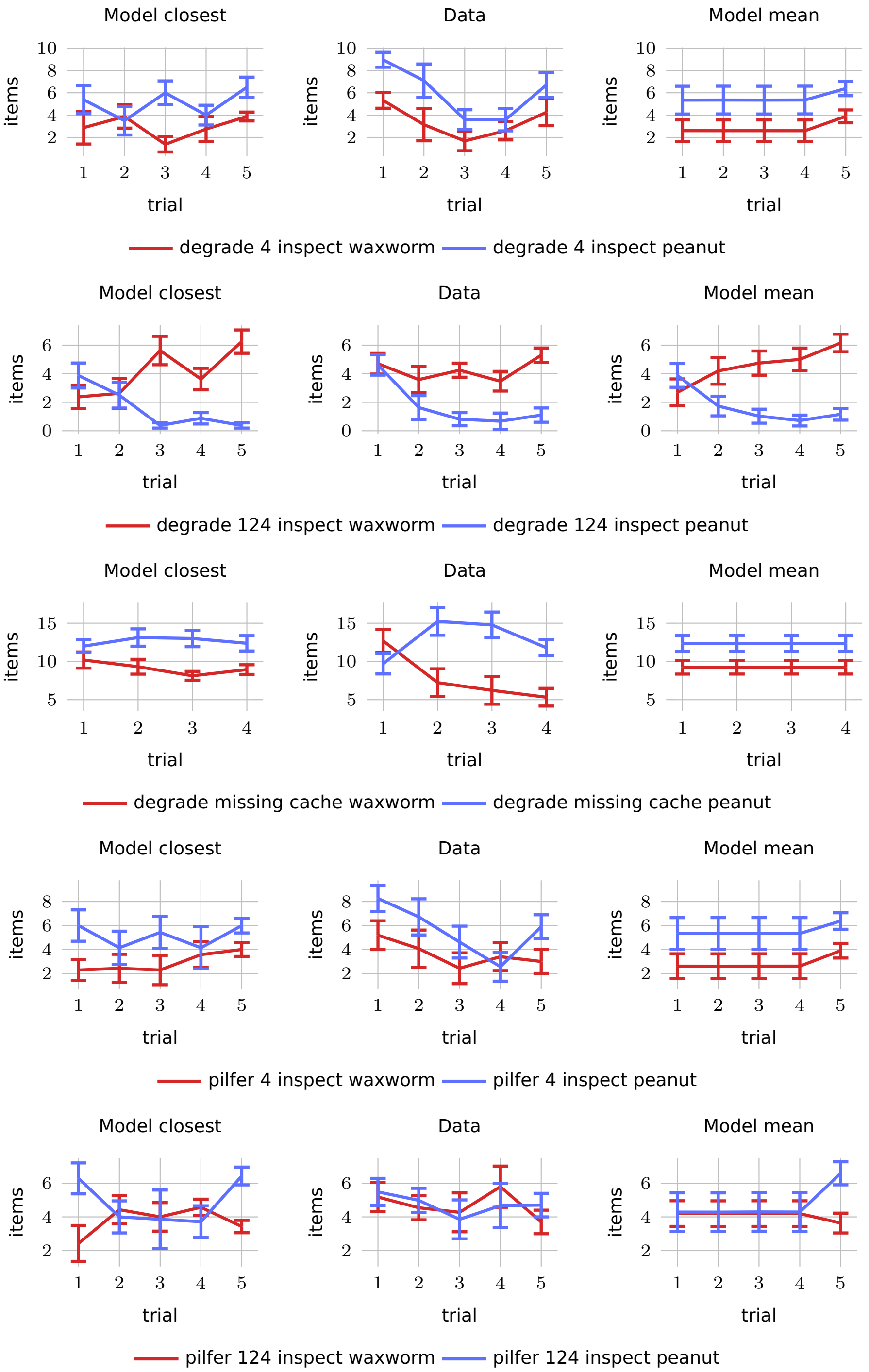

Figure S80: **Clayton99B exp1: Plastic Caching Model (part1)**

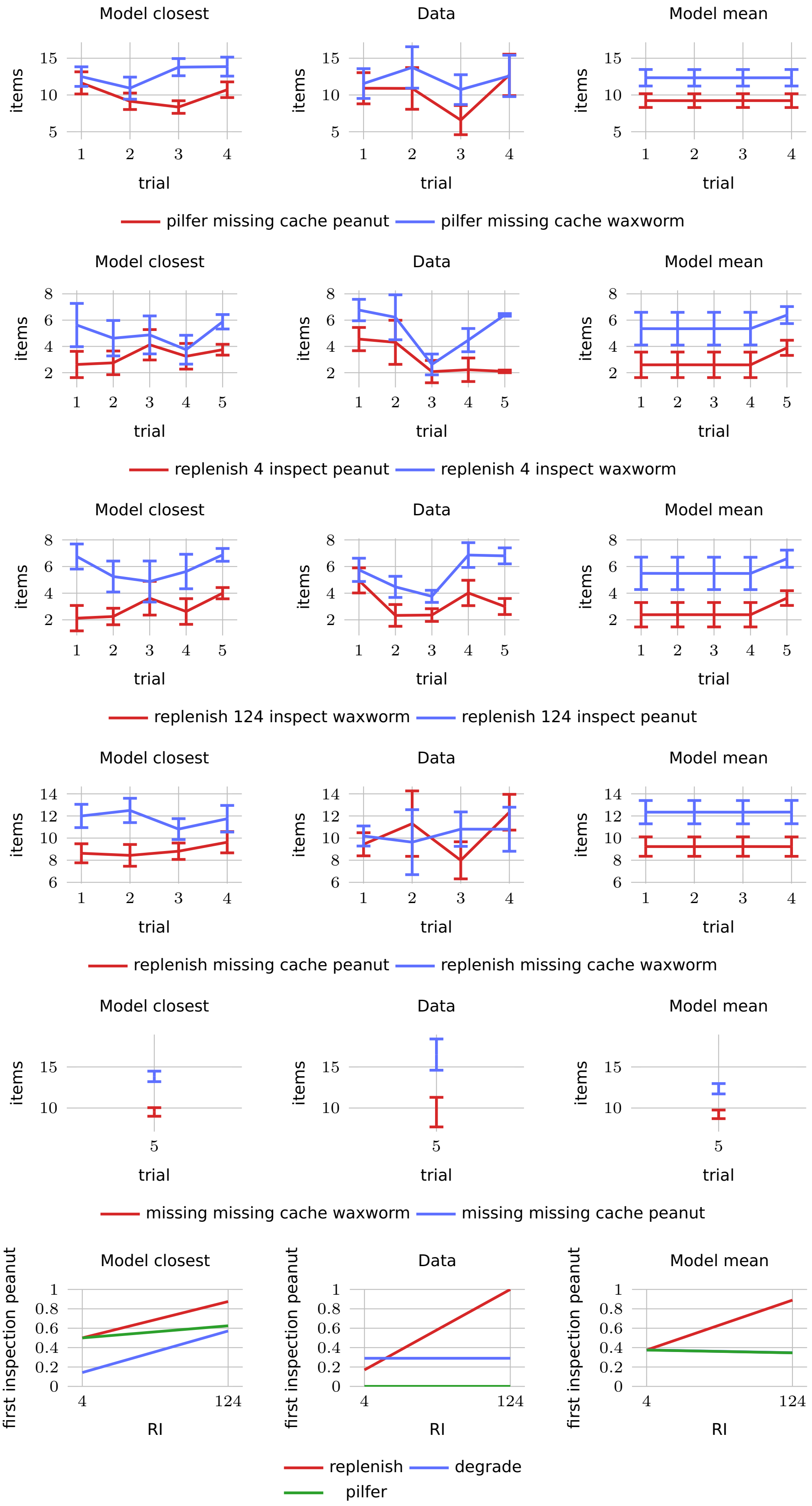

Figure S81: **Clayton99B exp1: Plastic Caching Model (part2)**

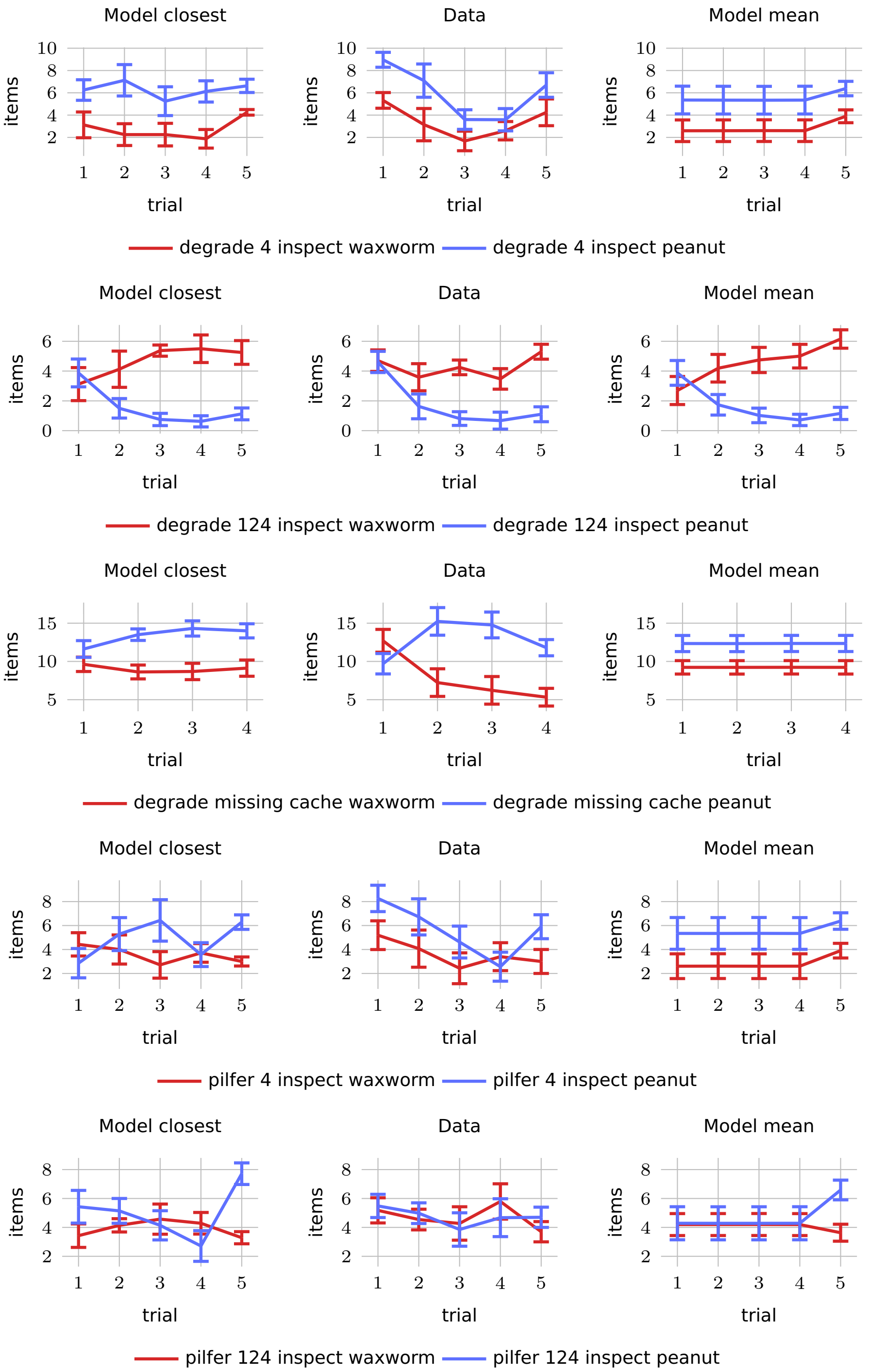

Figure S82: **Clayton99B exp1: Planning-By-Replay Model (part1)**

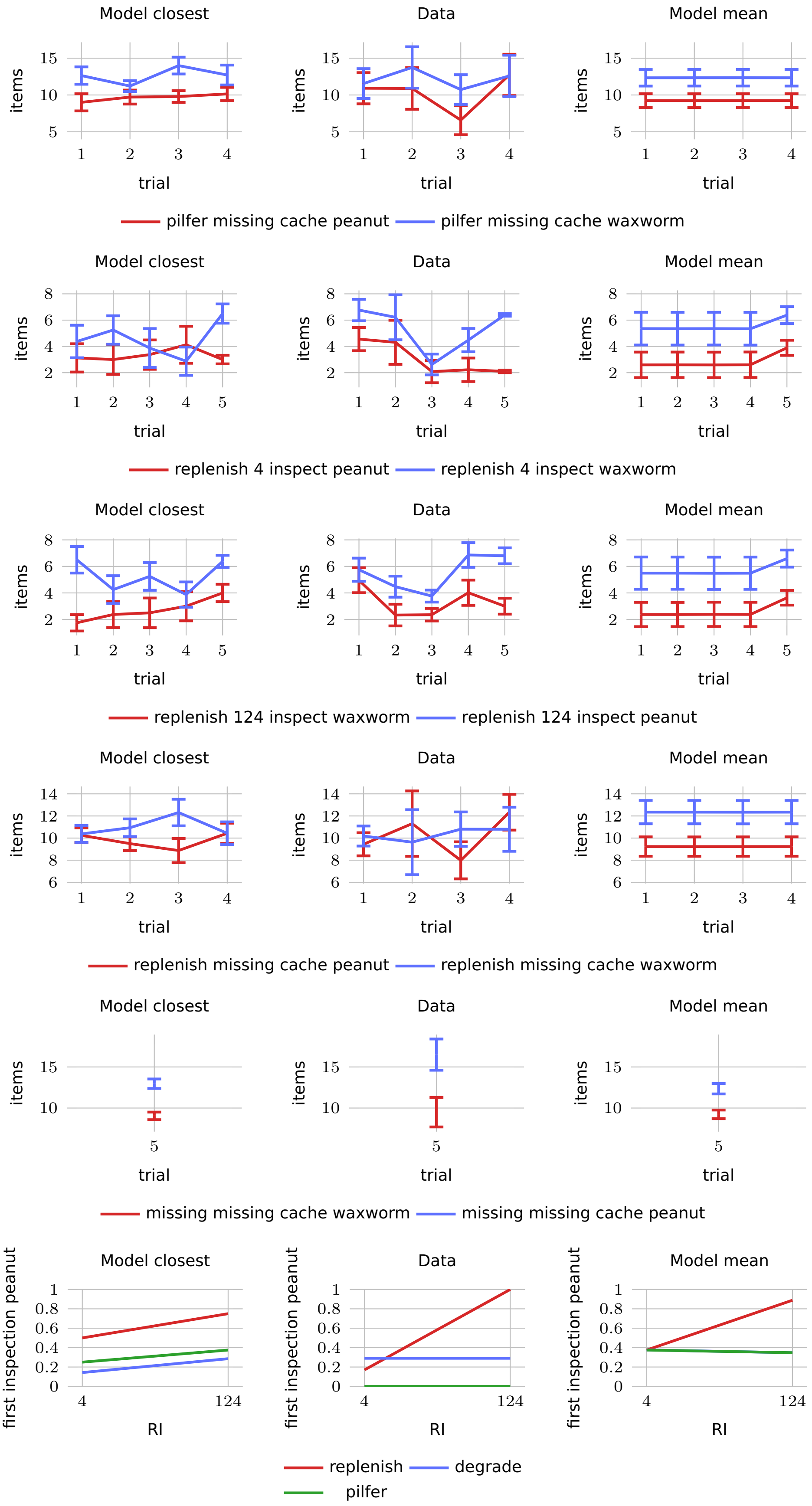

Figure S83: **Clayton99B exp1: Planning-By-Replay Model (part2)**

2.1.15 Clayton99B exp2

Published in [4].

Major Finding

Also when the birds cache in two sides of the same ice-cube tray they adapt their behaviour as in experiment 1.

Most Important Tests

test inspect (order): $F(1, 14) = 7.0, p = 0.019$

test inspect wp (group & foodtype): $F(2, 14) = 9.59, p = 0.0024$

test inspect pw (group & foodtype): $F(2, 14) < 1, p = 0.4$

Total Number of Statistics

31 p-values, 92 means or sems.

Figures

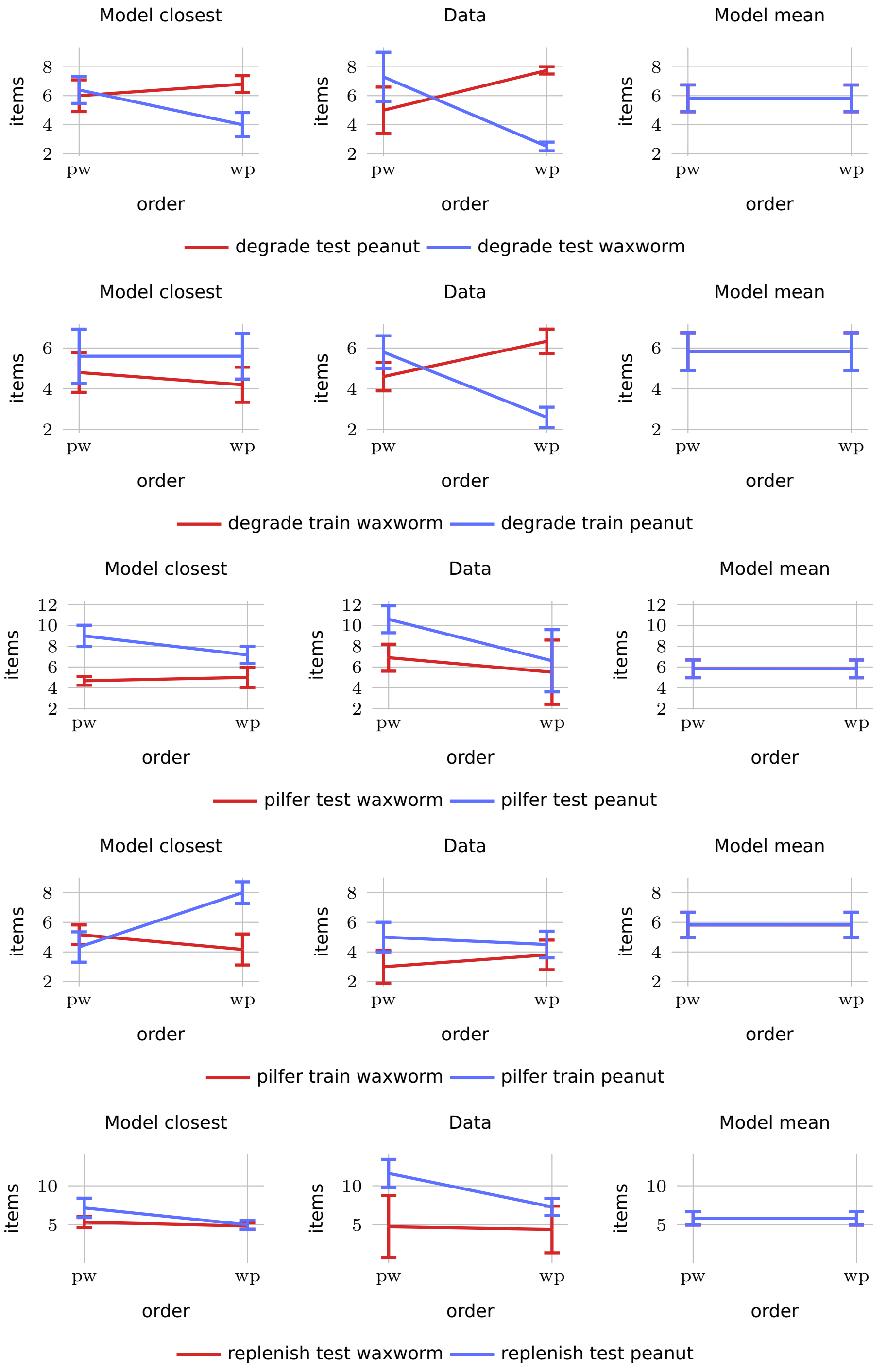

Figure S84: **Clayton99B exp2: No-Plasticity-No-Memory-No-Motivational-Control Model (part1)**

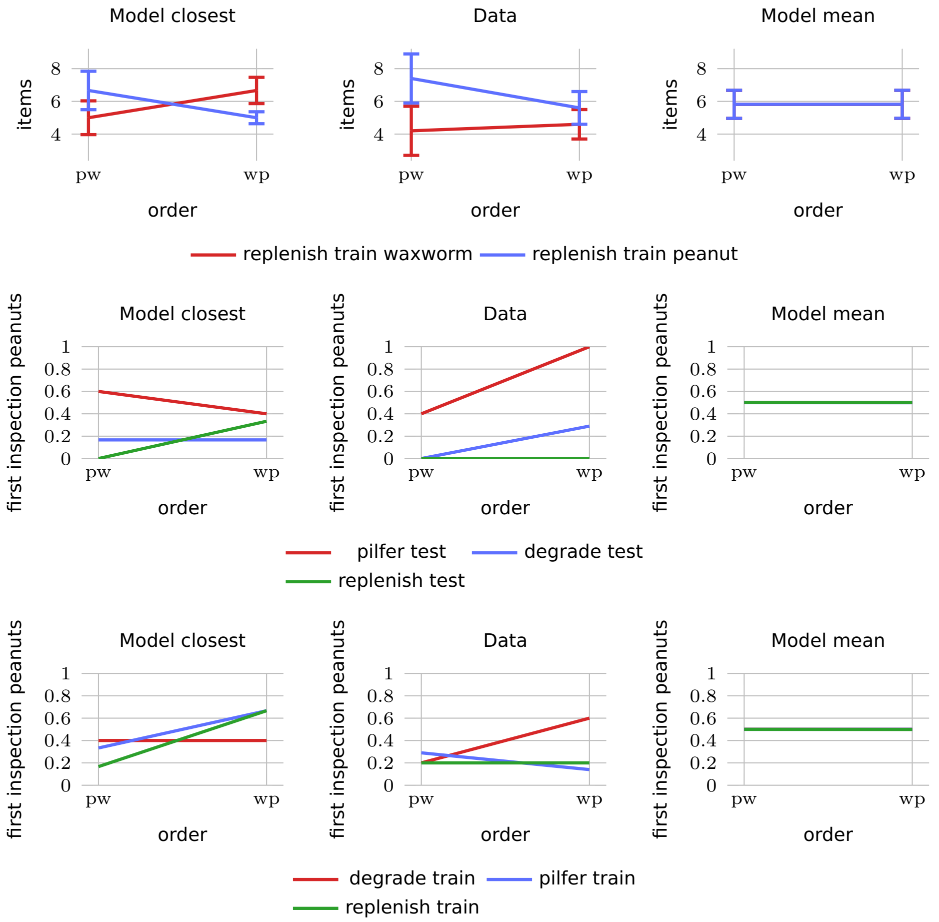

Figure S85: **Clayton99B exp2: No-Plasticity-No-Memory-No-Motivational-Control Model (part2)**

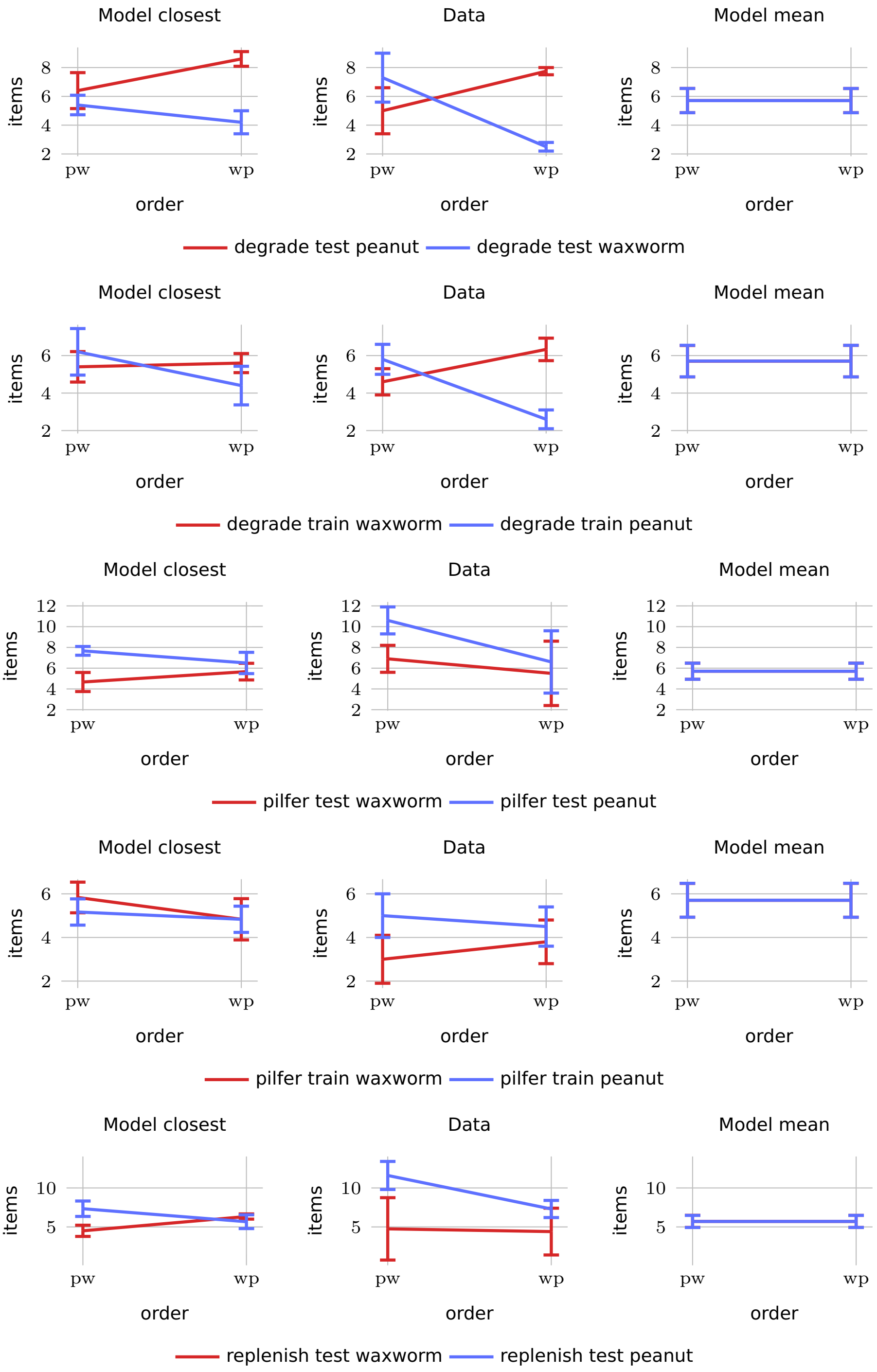

Figure S86: **Clayton99B exp2: No-Plasticity-No-Memory Model (part1)**

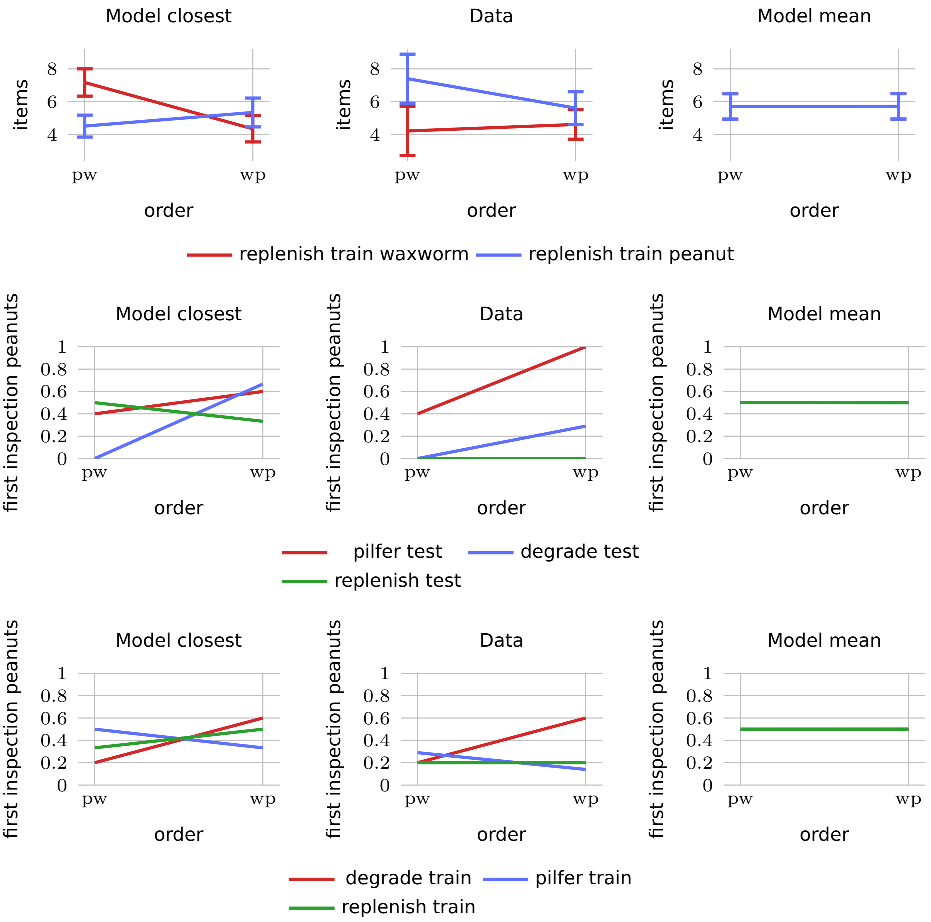

Figure S87: **Clayton99B exp2: No-Plasticity-No-Memory Model (part2)**

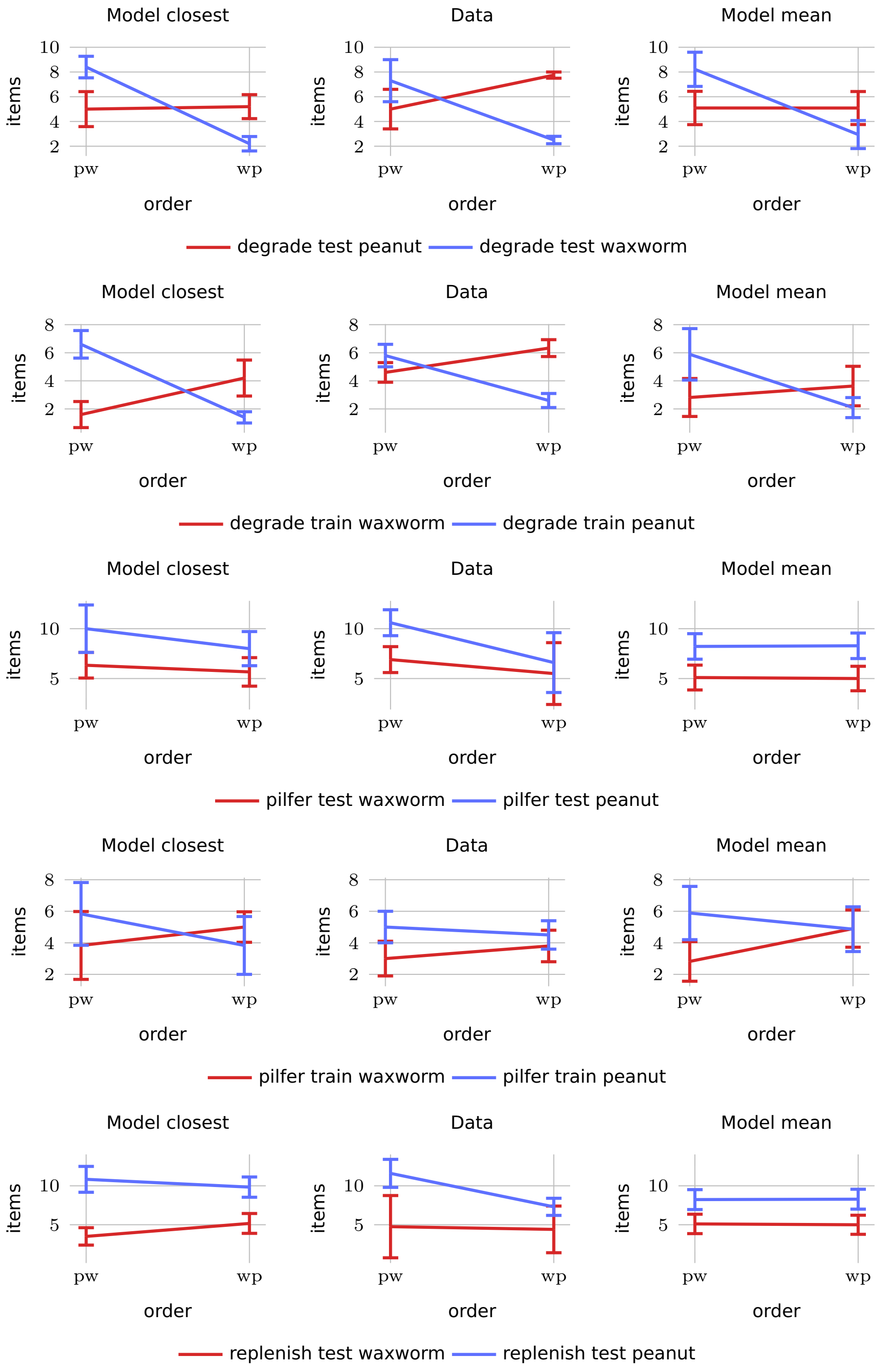

Figure S88: **Clayton99B exp2: No-Plasticity Model (part1)**

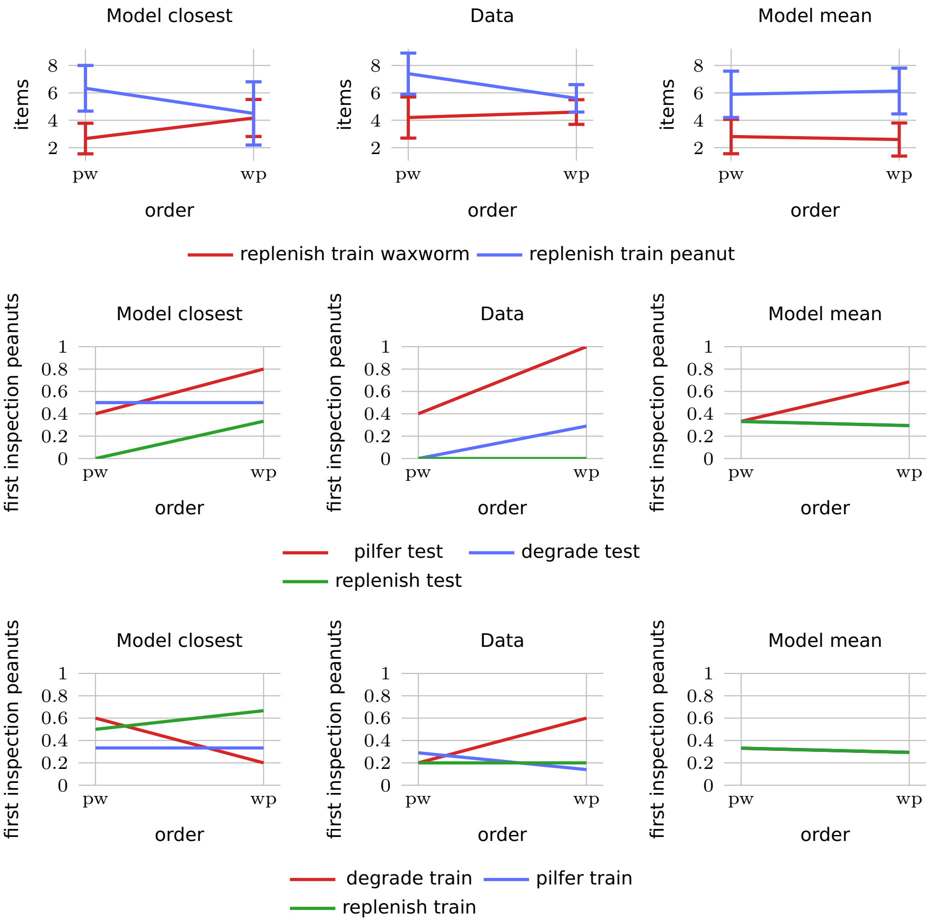

Figure S89: **Clayton99B exp2: No-Plasticity Model (part2)**

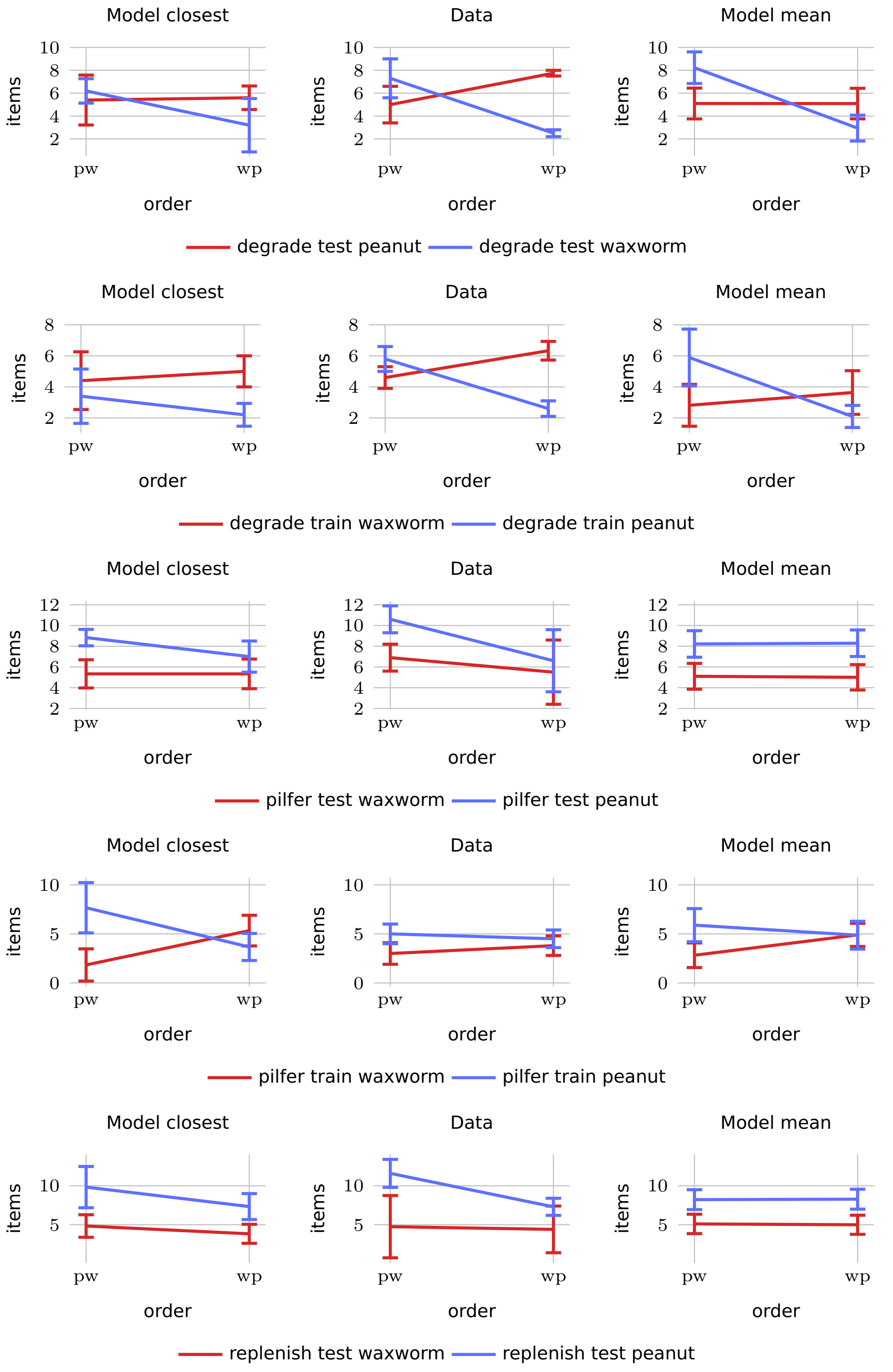

Figure S90: **Clayton99B exp2: Plastic Caching Model (part1)**

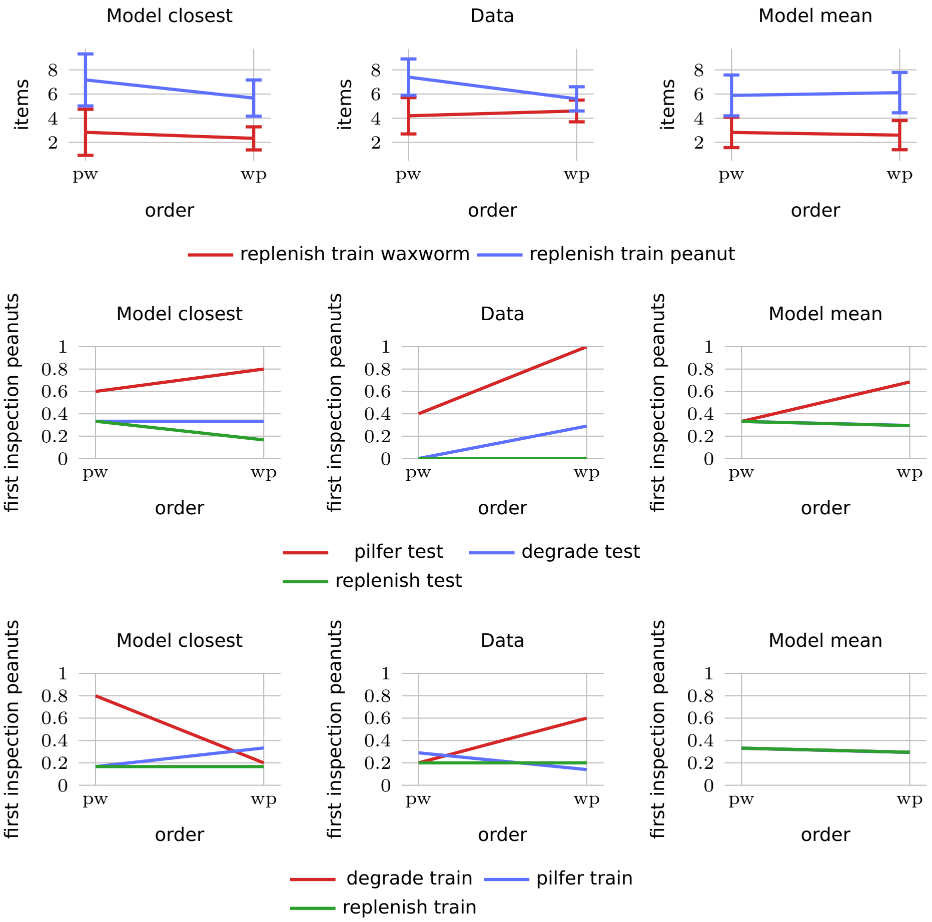

Figure S91: **Clayton99B exp2: Plastic Caching Model (part2)**

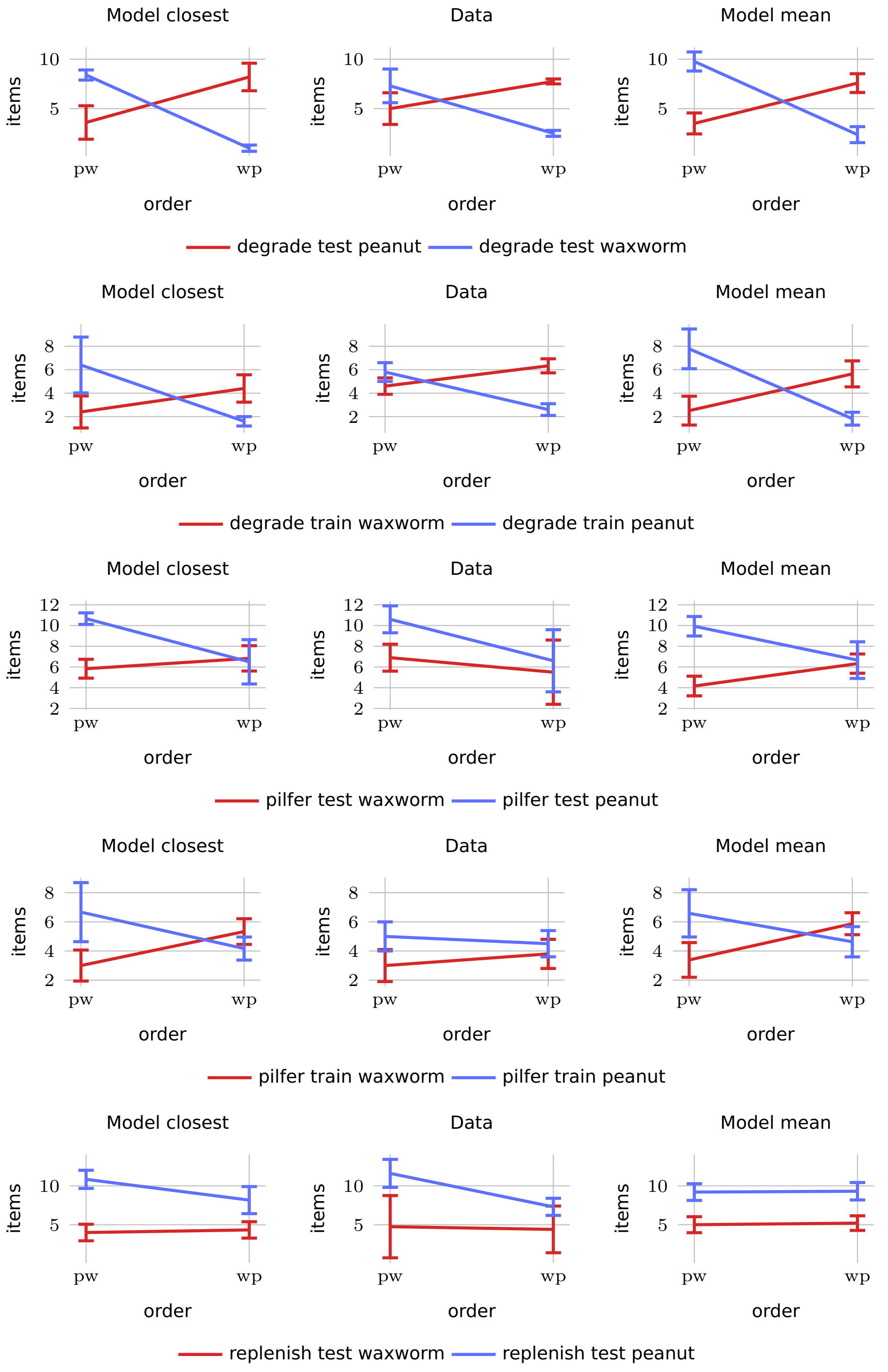

Figure S92: **Clayton99B exp2: Planning-By-Replay Model (part1)**

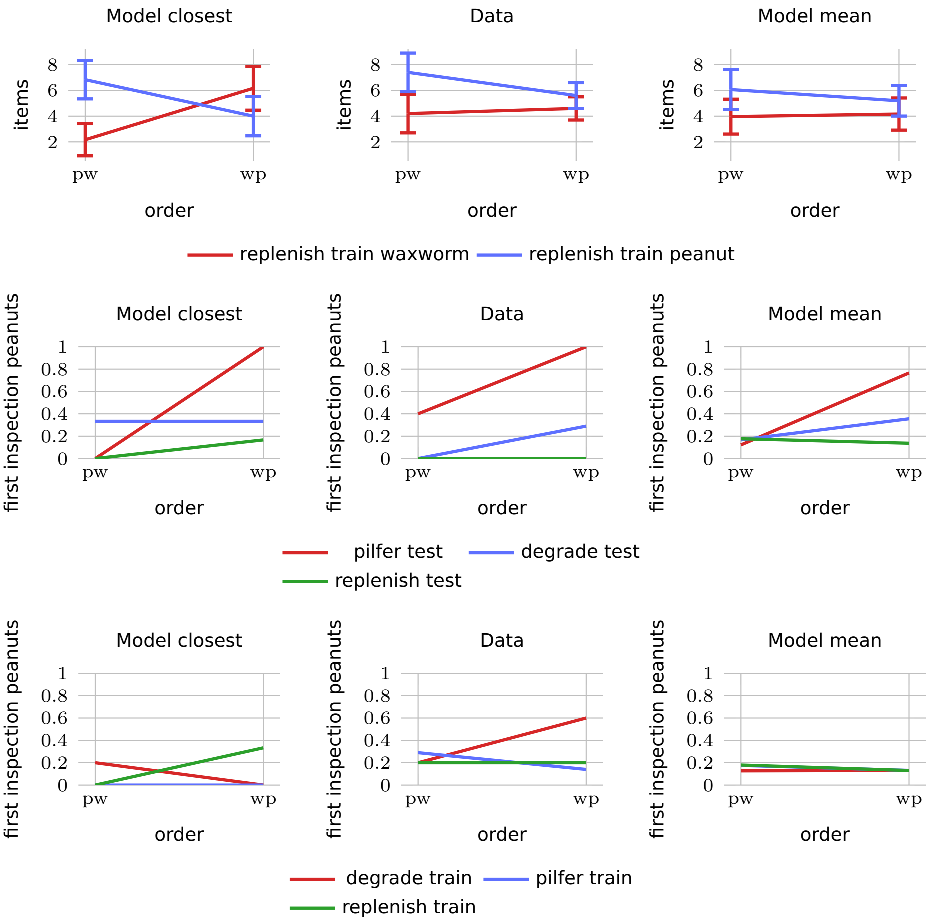

Figure S93: **Clayton99B exp2: Planning-By-Replay Model (part2)**

2.1.16 Clayton01 exp1

Published in [5].

Major Finding

The birds learn not to search for their preferred food after retention intervals where they experienced the preferred food to be degraded.

Comments

The choices between peanuts and waxworms or peanuts and crickets were investigated here. There was no significant effect in the analysis of the overall searches for the intermediate retention interval, but a significant difference in between the patterns of first searches shown by the two groups.

Most Important Tests

first inspect test RI 4 (group): $Chisq(2) = 0.27, p = 0.87$

first inspect test RI 100 (group): $Chisq(3) = 13.0, p = 0.0046$

first inspect test RI 28 (group): $Chisq(2) = 9.65, p = 0.008$

Total Number of Statistics

24 p-values, 122 means or sems.

Figures

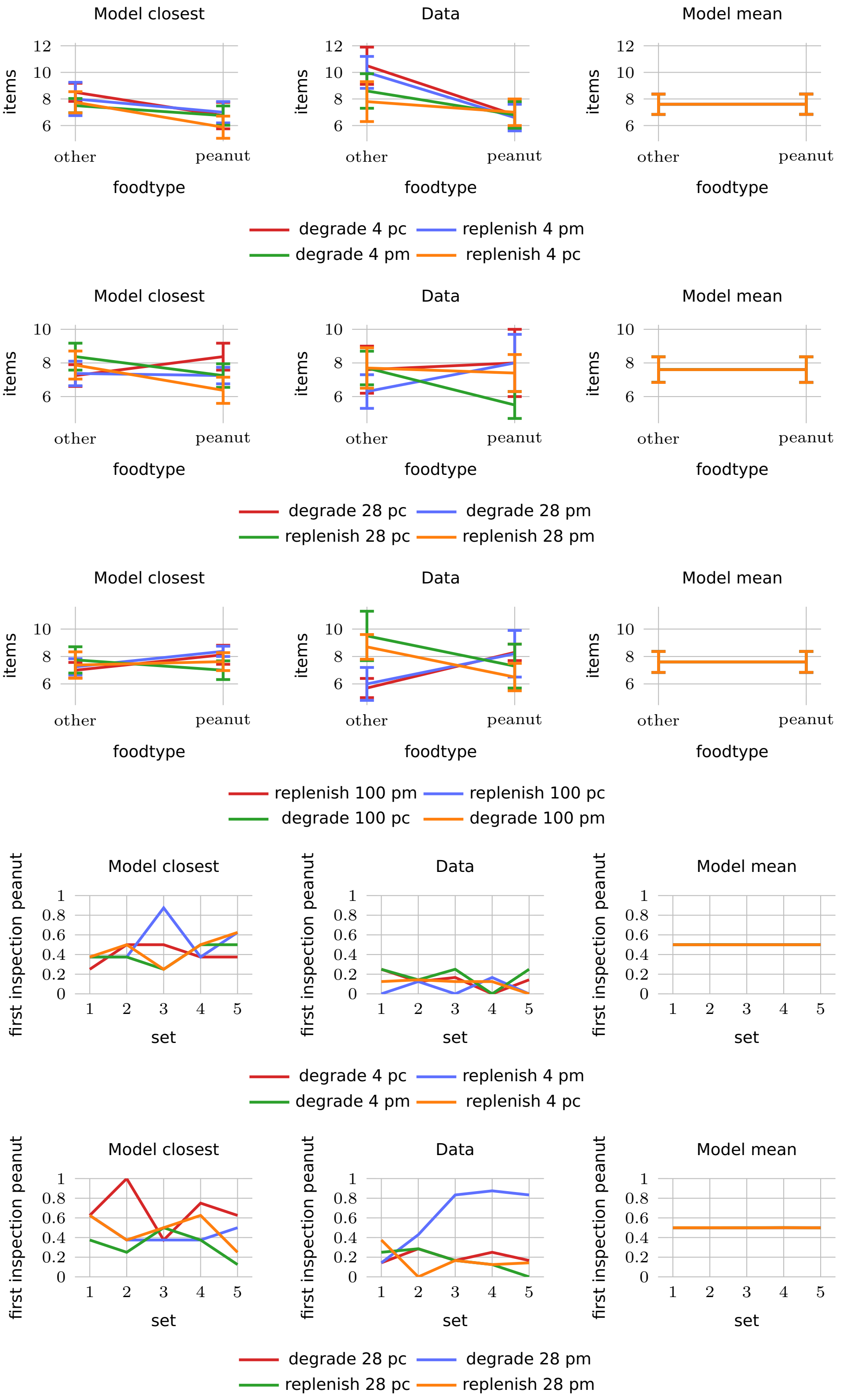

Figure S94: **Clayton01 exp1: No-Plasticity-No-Memory-No-Motivational-Control Model (part1)**

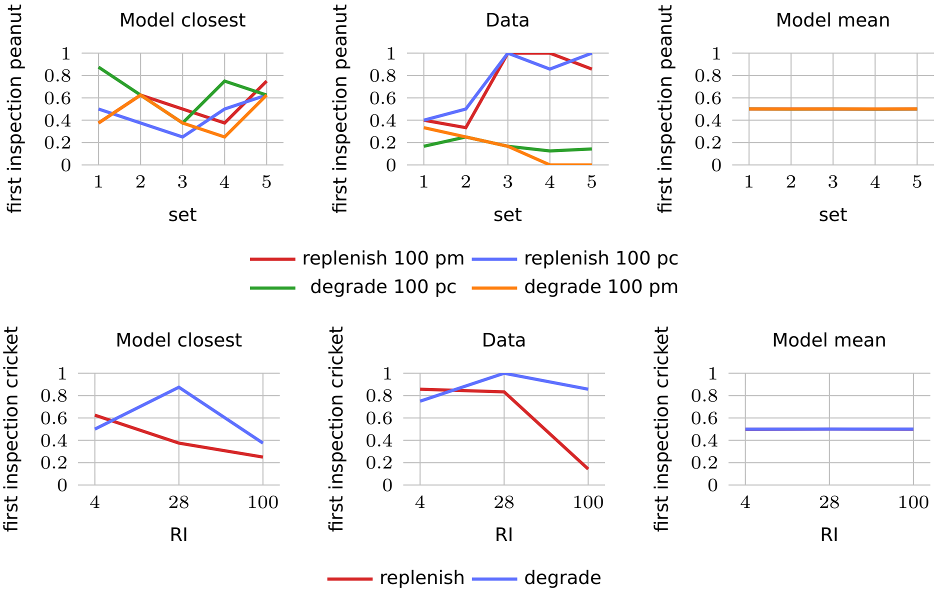

Figure S95: **Clayton01 exp1: No-Plasticity-No-Memory-No-Motivational-Control Model (part2)**

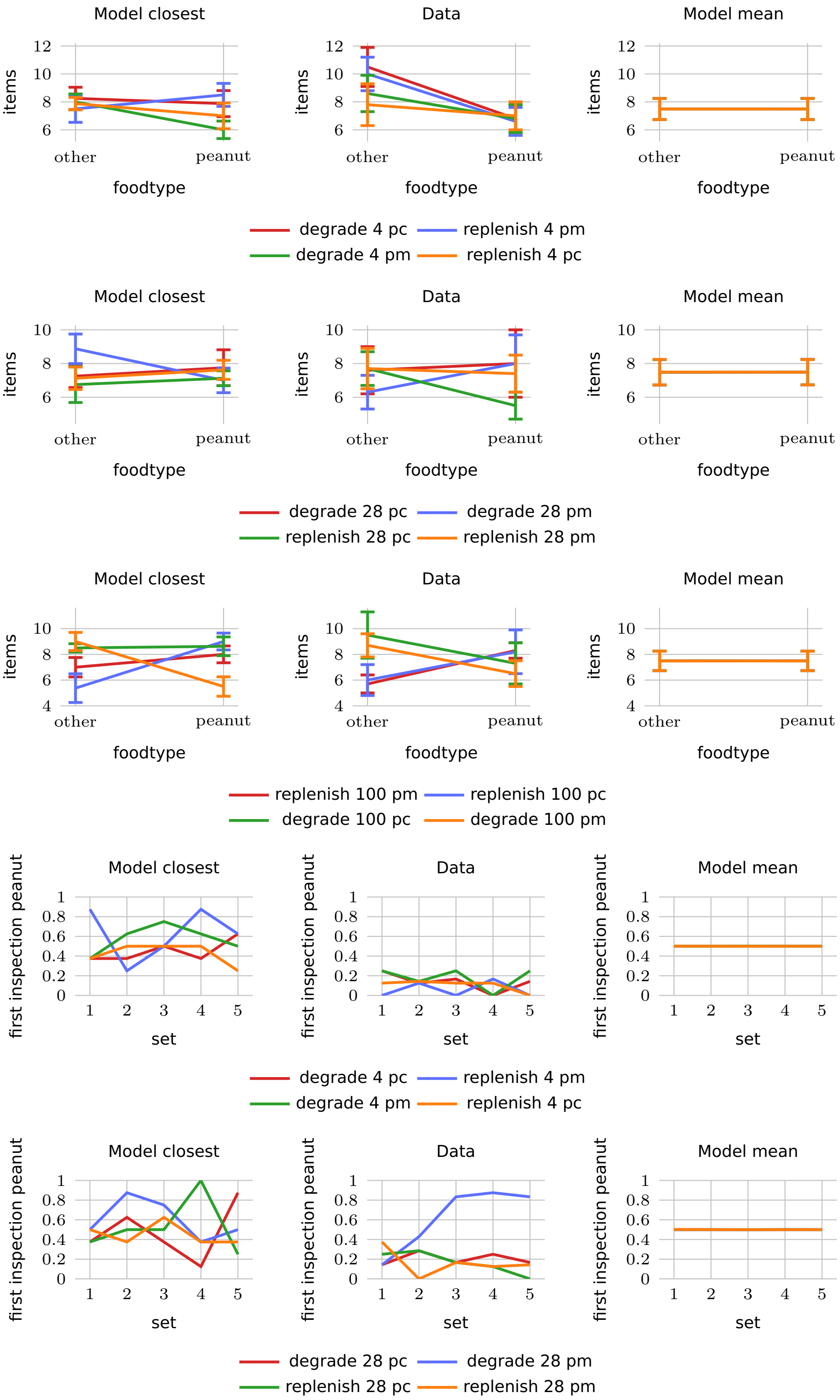

Figure S96: **Clayton01 exp1: No-Plasticity-No-Memory Model (part1)**

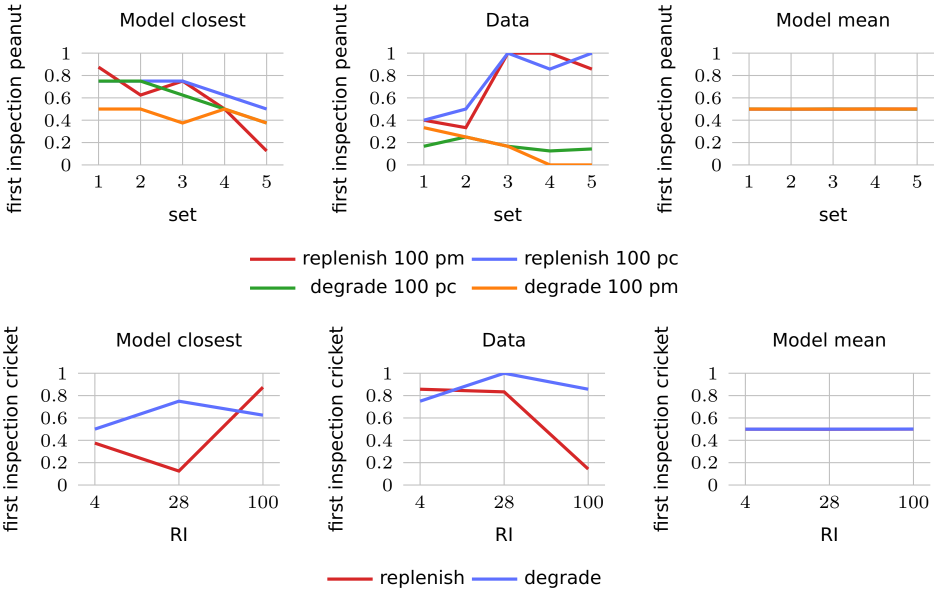

Figure S97: **Clayton01 exp1: No-Plasticity-No-Memory Model (part2)**

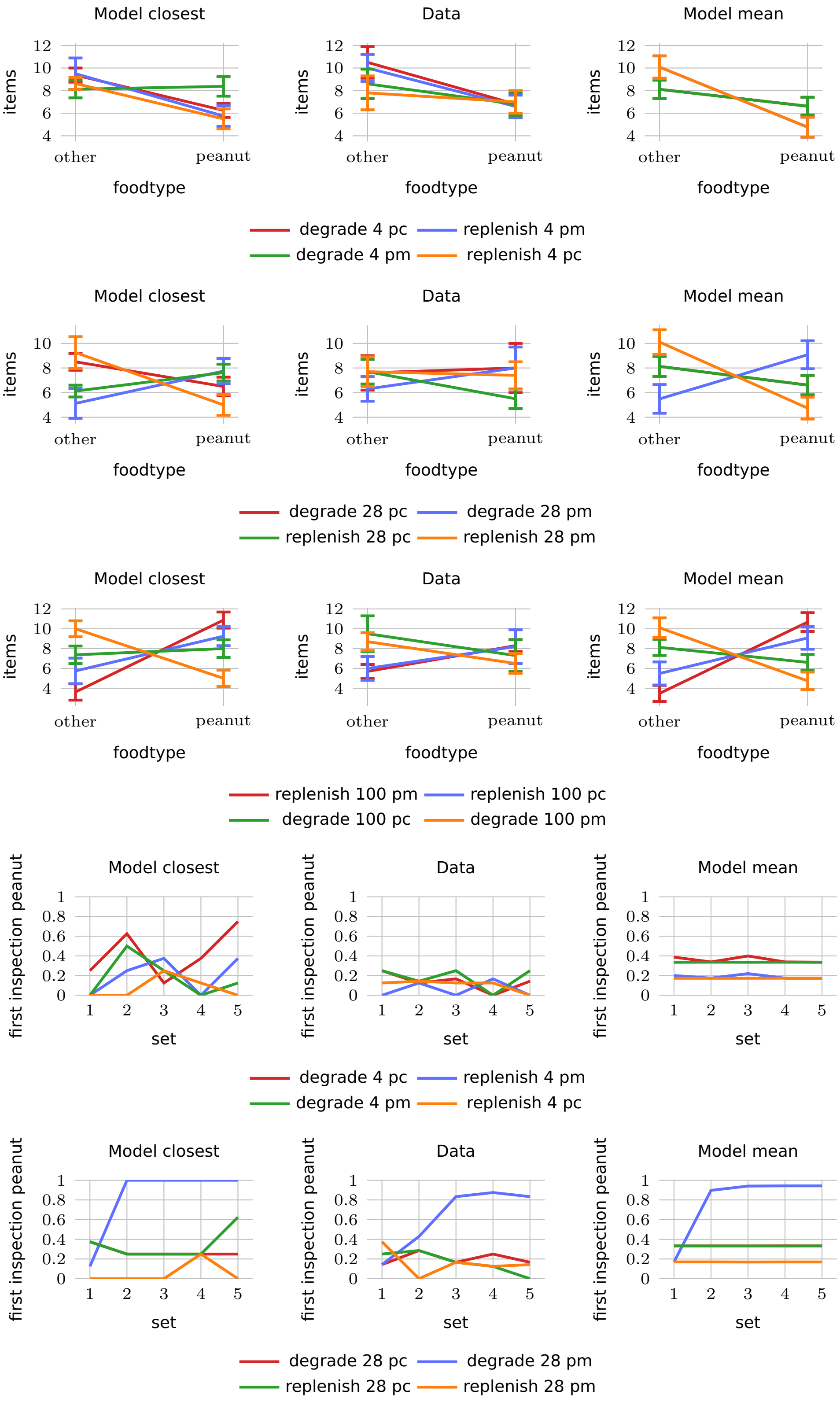

Figure S98: **Clayton01 exp1: No-Plasticity Model (part1)**

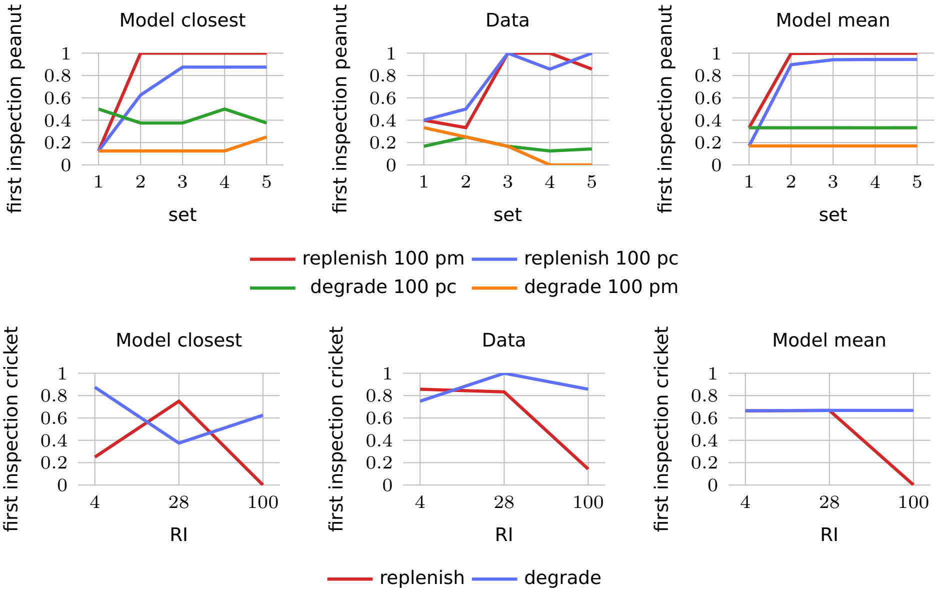

Figure S99: **Clayton01 exp1: No-Plasticity Model (part2)**

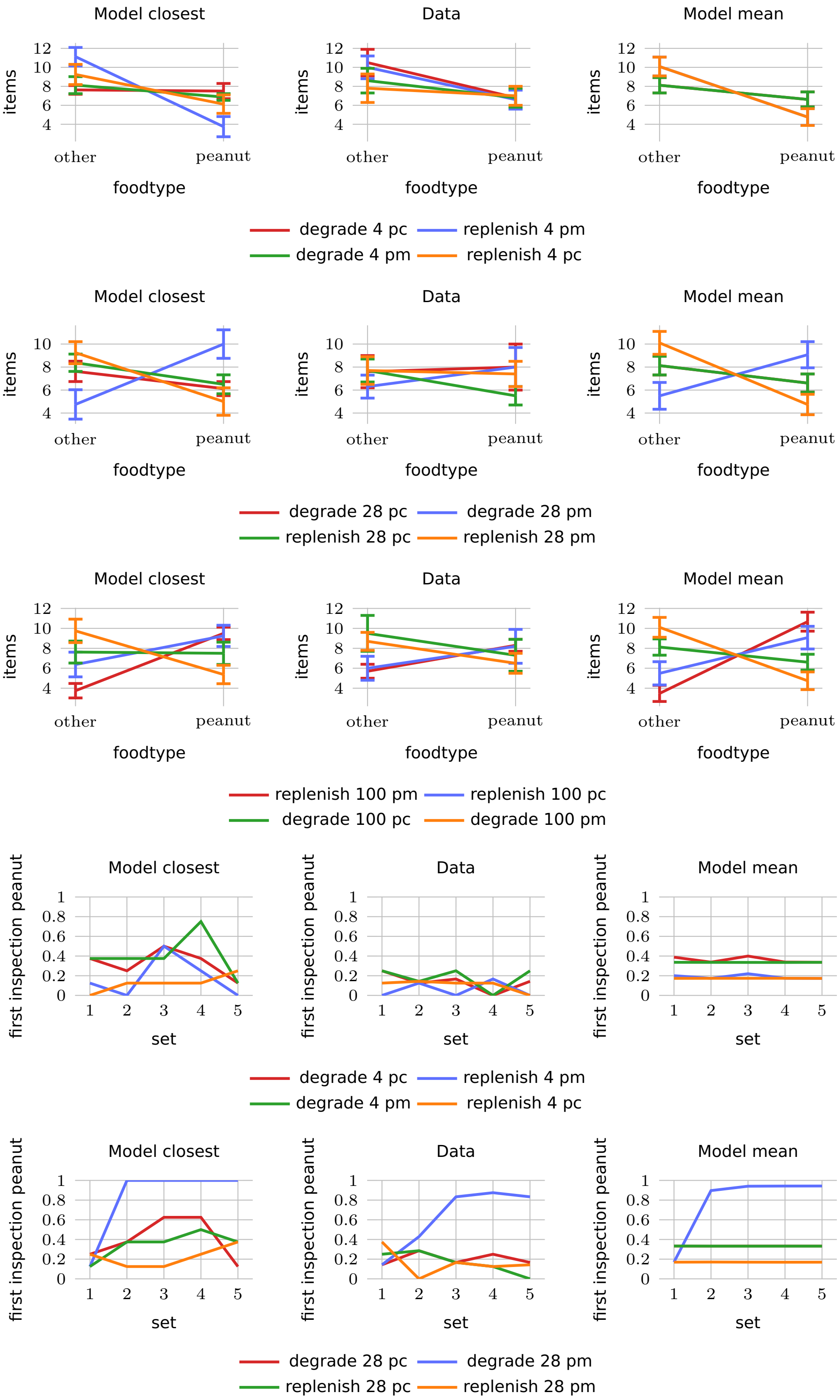

Figure S100: **Clayton01 exp1: Plastic Caching Model (part1)**

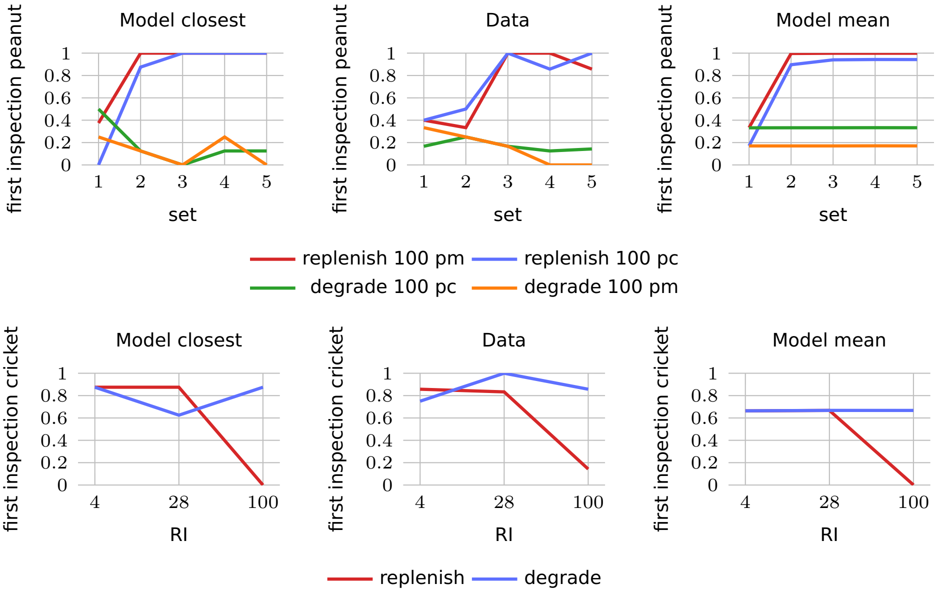

Figure S101: **Clayton01 exp1: Plastic Caching Model (part2)**

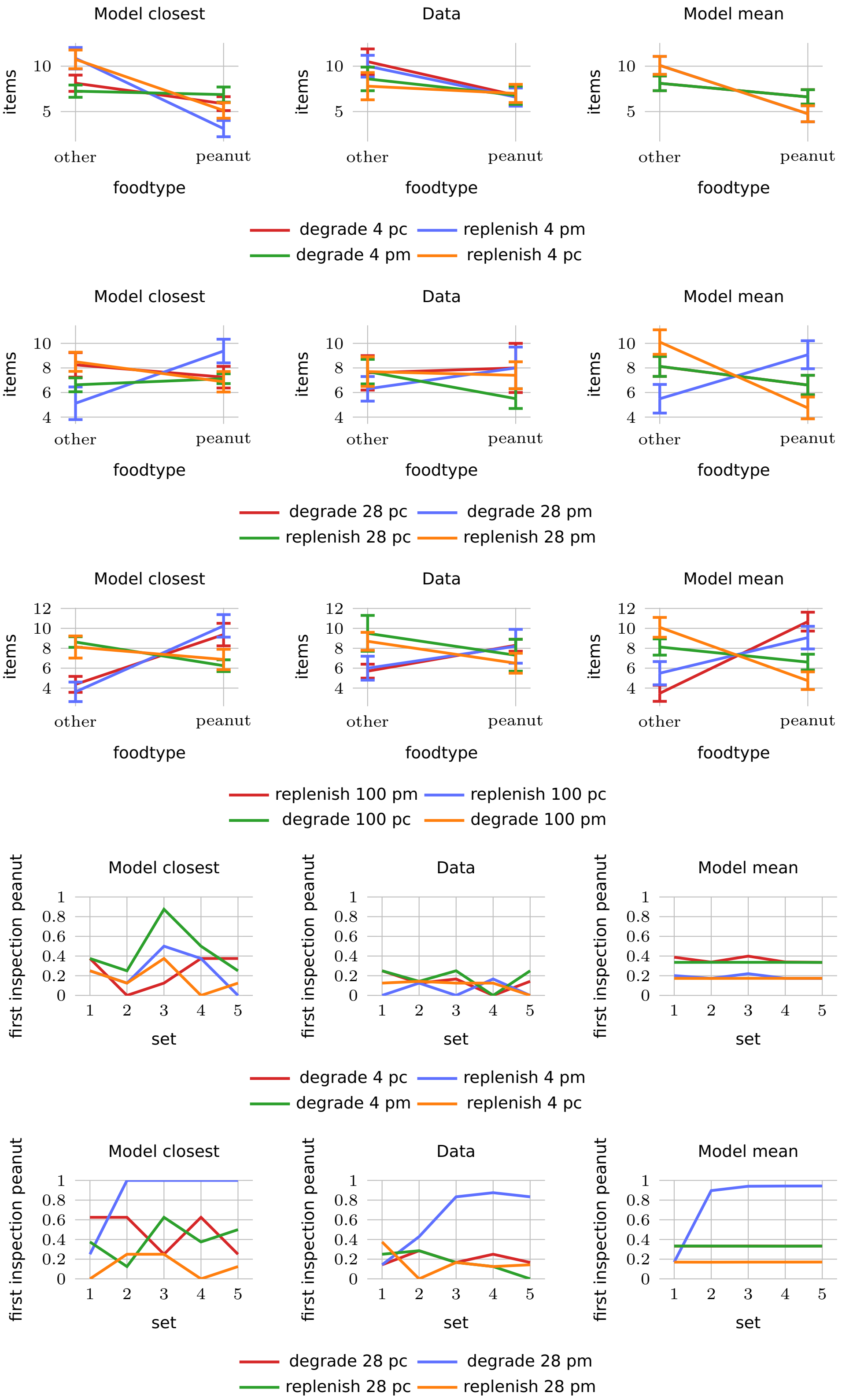

Figure S102: **Clayton01 exp1: Planning-By-Replay Model (part1)**

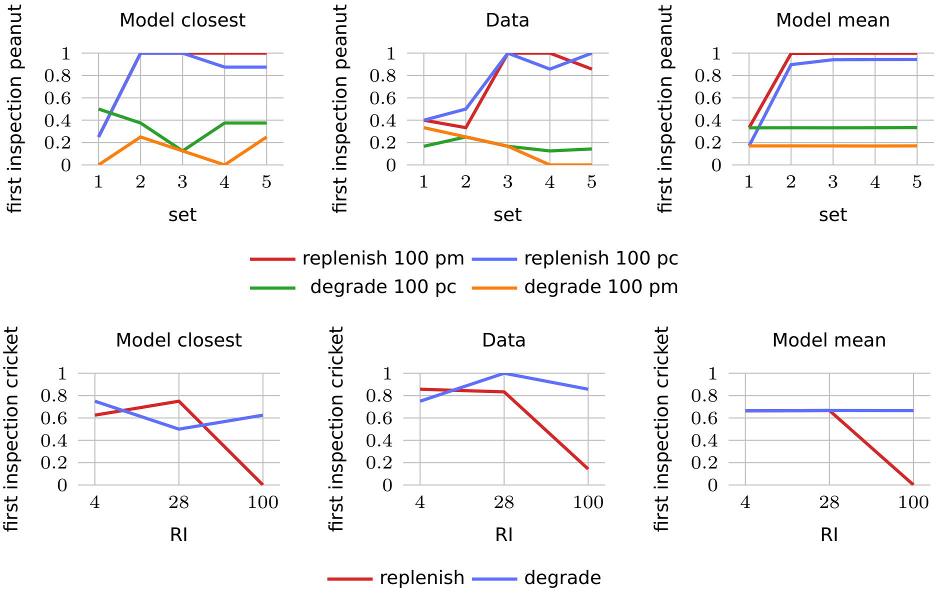

Figure S103: **Clayton01 exp1: Planning-By-Replay Model (part2)**

2.1.17 Clayton01 exp2

Published in [5].

Major Finding

Confirming experiment 1, birds search their preferred food after a short retention interval, where both food types are expected to be fresh, but they reverse this behaviour after a longer retention interval, where the preferred food is expected to be degraded.

Comments

The choice between mealworms (preferred) and crickets was investigated.

Most Important Tests

inspect test exp 2 RI 4 (foodtype): $F(1,14) = 30.72, p = 7.3 \cdot 10^{-5}$

inspect test exp 2 RI 28 (group & foodtype): $F(1,14) = 5.28, p = 0.038$

inspect test exp 2 (group & foodtype & RI): $F(1,14) = 12.53, p = 0.0033$

Total Number of Statistics

17 p-values, 28 means or sems.

Figures

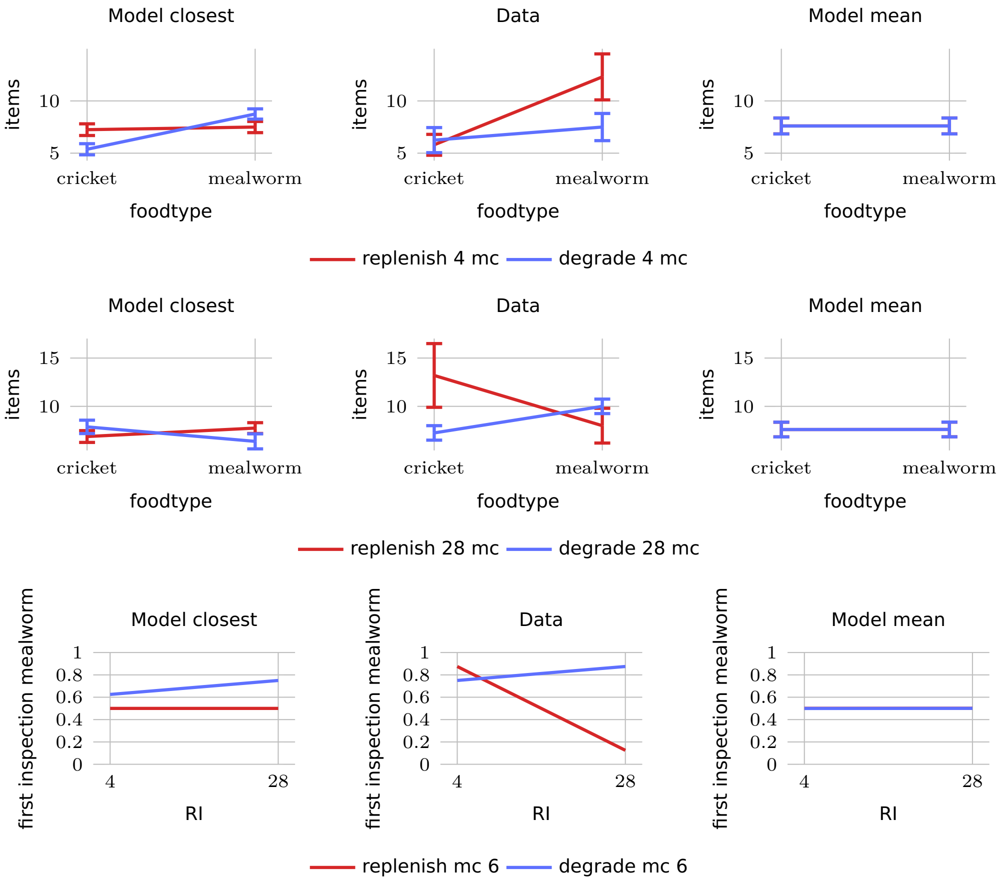

Figure S104: **Clayton01 exp2: No-Plasticity-No-Memory-No-Motivational-Control Model**

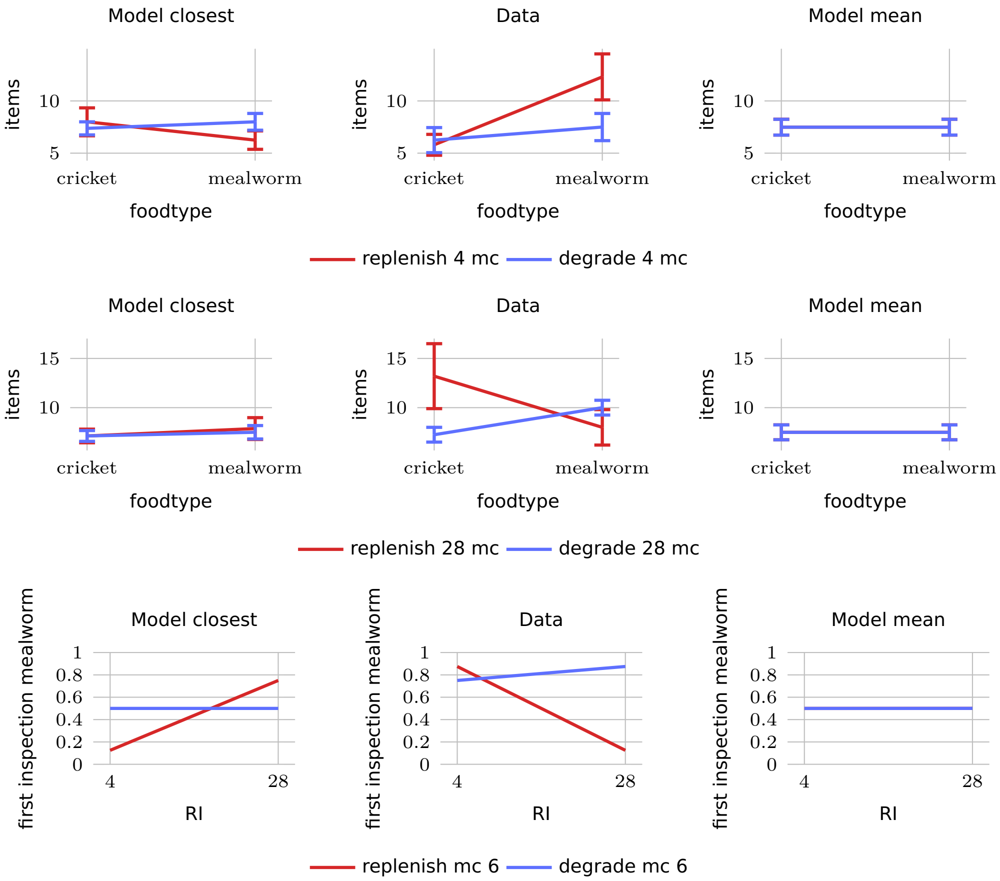

Figure S105: **Clayton01 exp2: No-Plasticity-No-Memory Model**

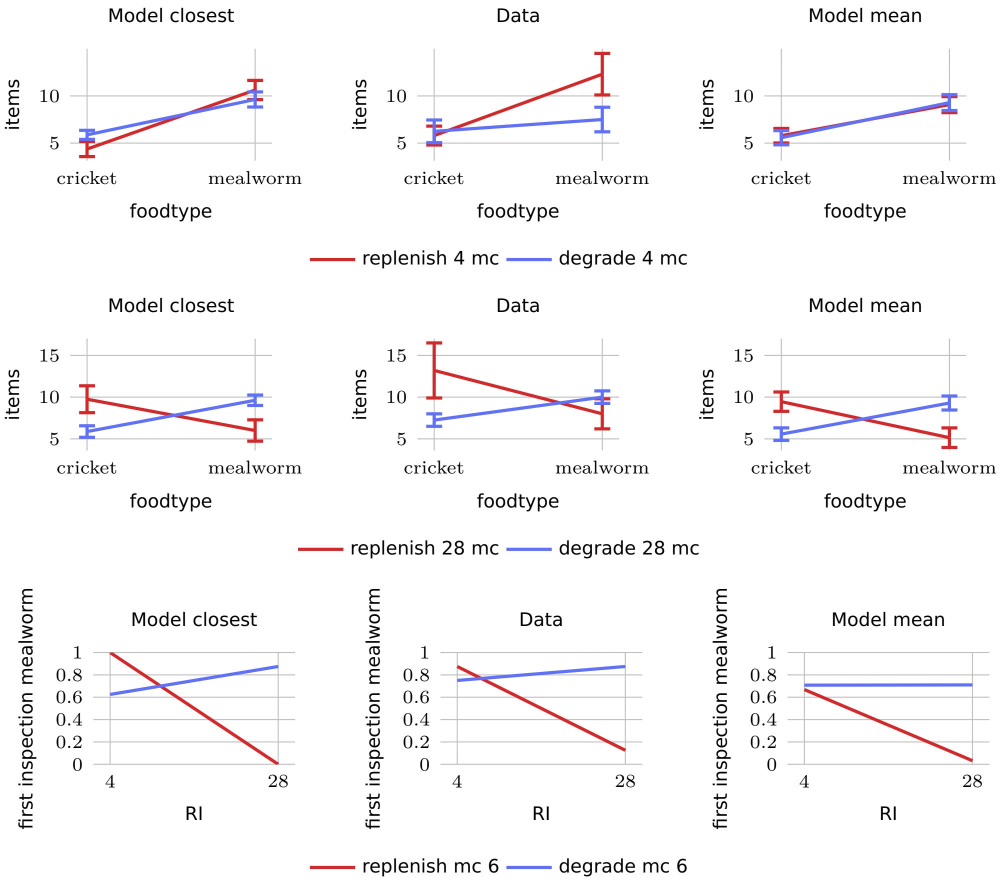

Figure S106: **Clayton01 exp2: No-Plasticity Model**

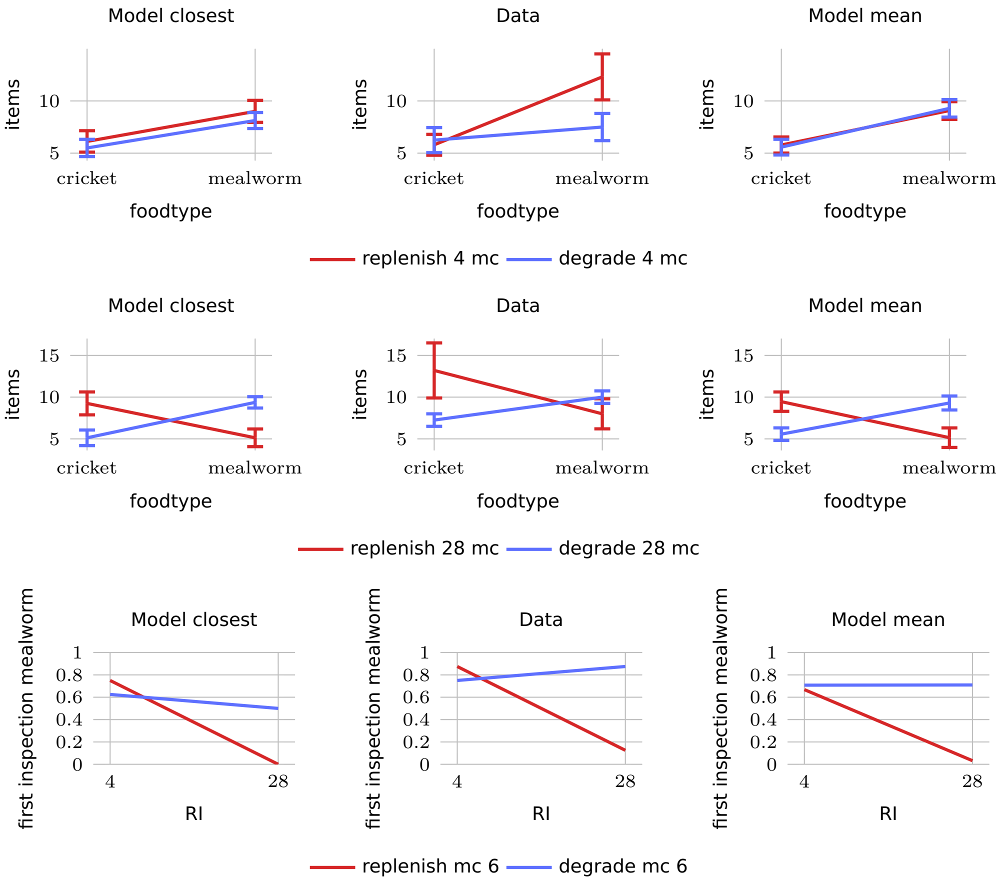

Figure S107: **Clayton01 exp2: Plastic Caching Model**

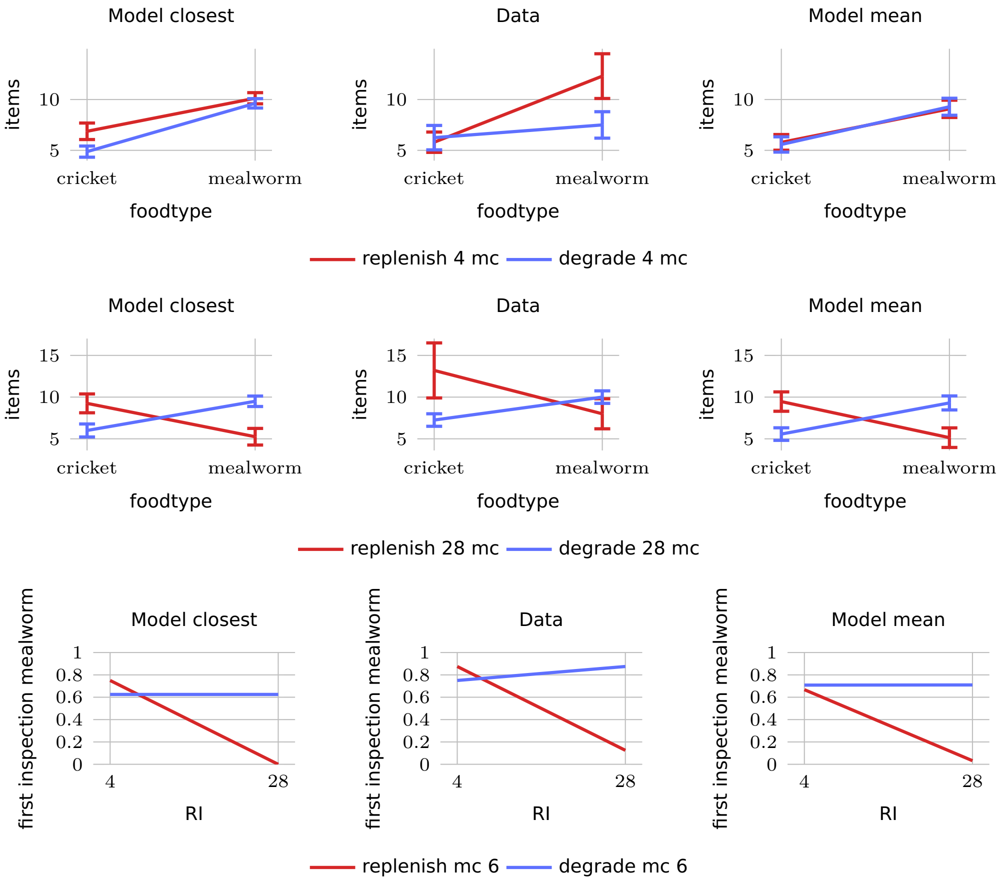

Figure S108: **Clayton01 exp2: Planning-By-Replay Model**

2.1.18 Clayton01 exp3

Published in [5].

Major Finding

The birds direct more searches to the unpreferred food after the long retention interval, even when the two types of food are cached in two sides of the same caching tray.

Most Important Tests

inspect cm (group & foodtype): $F(1,14) < 1, p = 0.34$

inspect mc (group & foodtype): $F(1,14) = 30.76, p = 7.2 \cdot 10^{-5}$

inspect degrade (foodtype & trial): $F(1,7) = 59.94, p = 0.00011$

Total Number of Statistics

16 p-values, 28 means or sems.

Figures

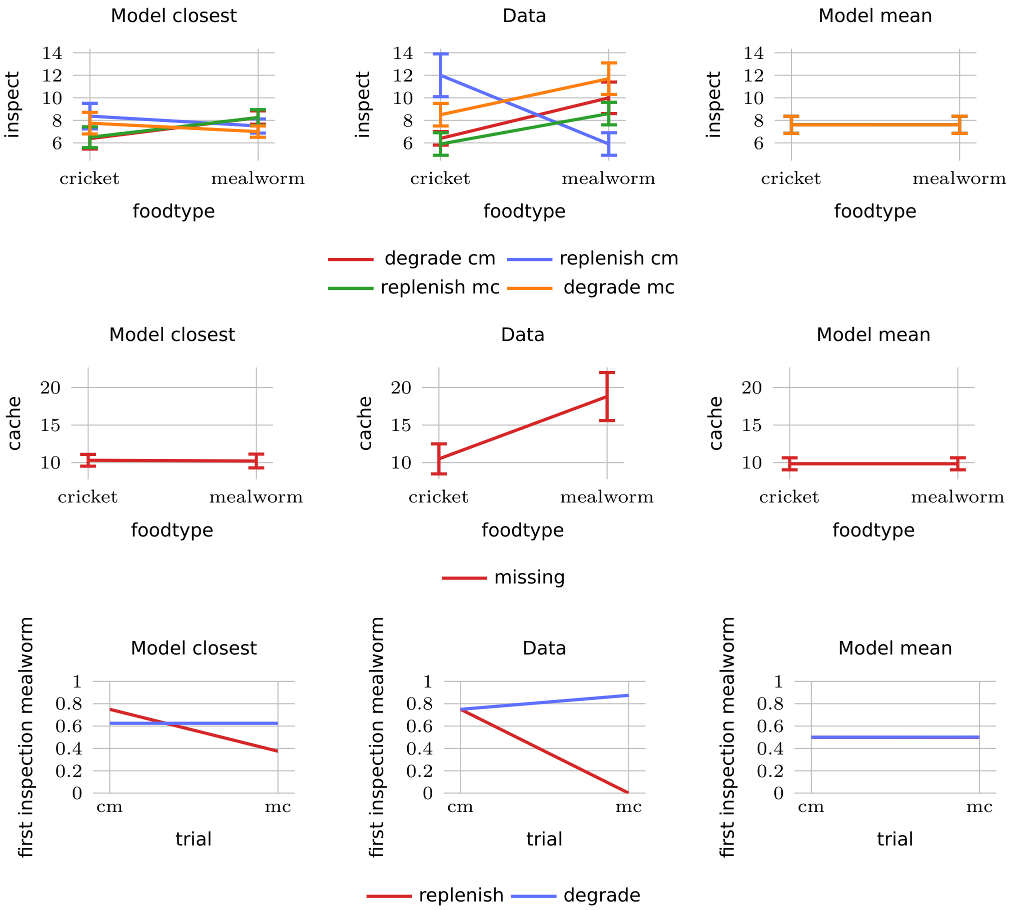

Figure S109: **Clayton01 exp3: No-Plasticity-No-Memory-No-Motivational-Control Model**

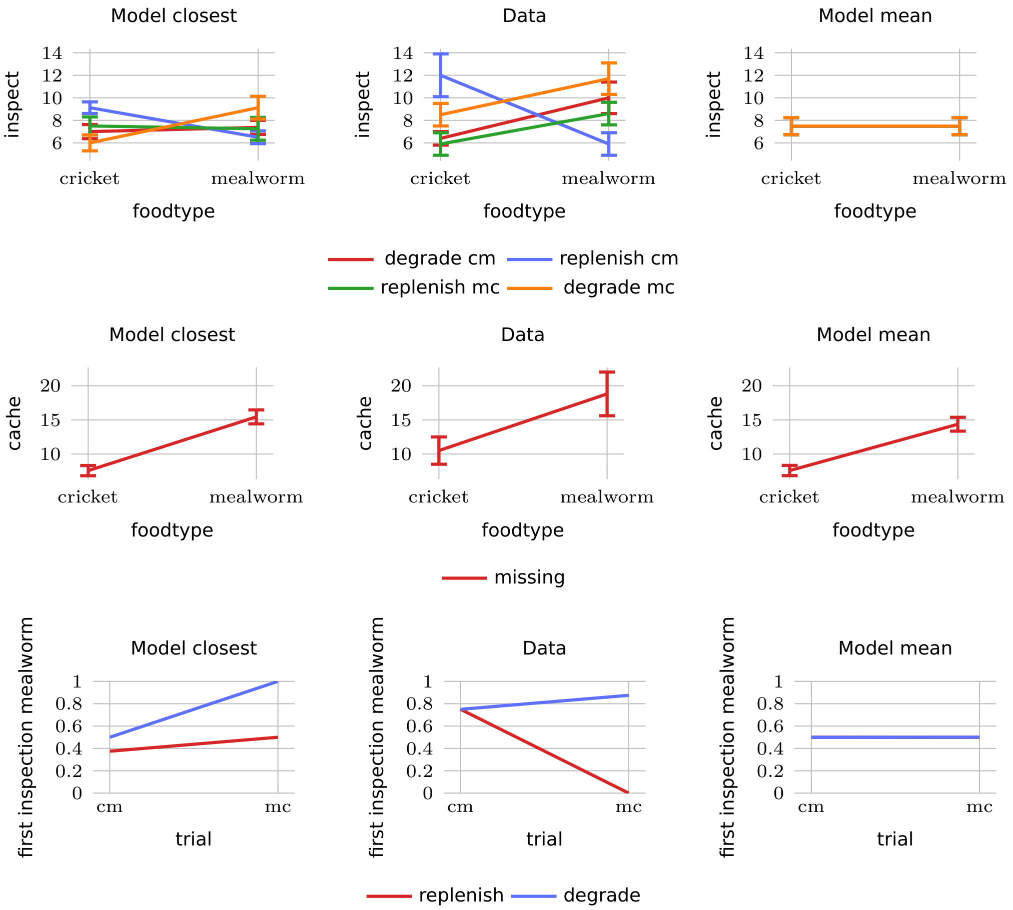

Figure S110: **Clayton01 exp3: No-Plasticity-No-Memory Model**

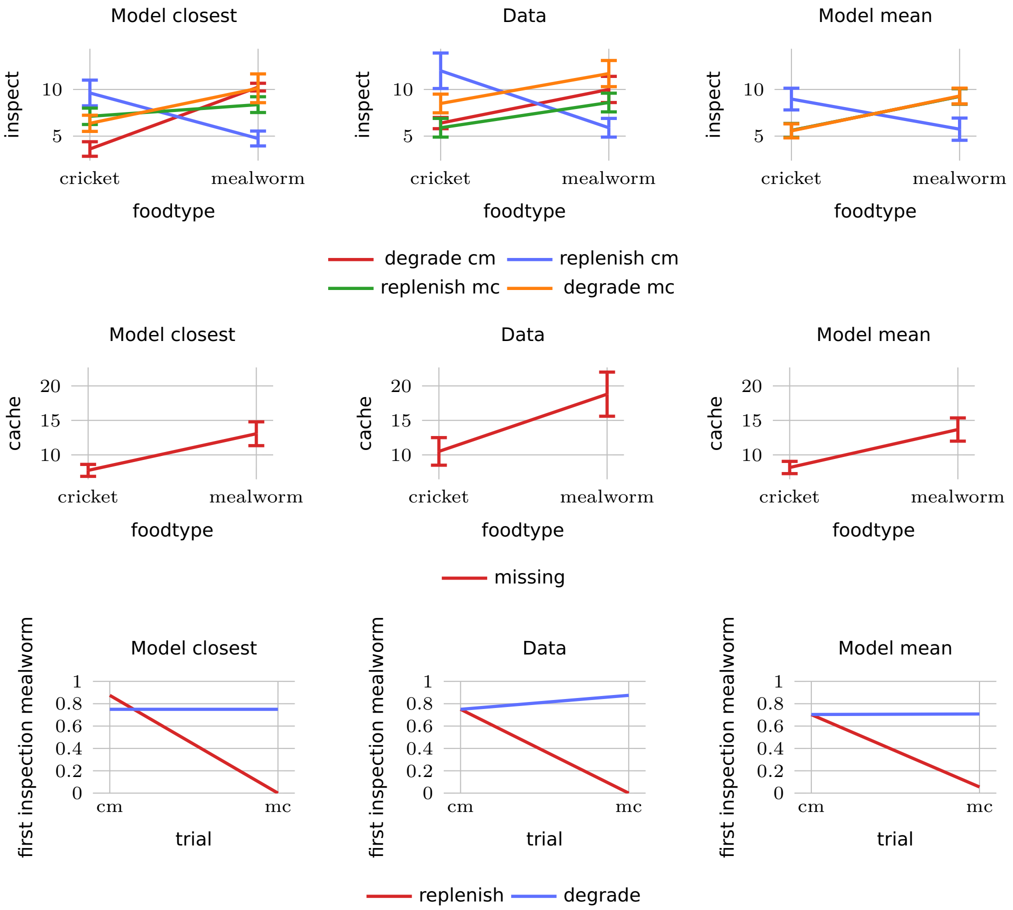

Figure S111: **Clayton01 exp3: No-Plasticity Model**

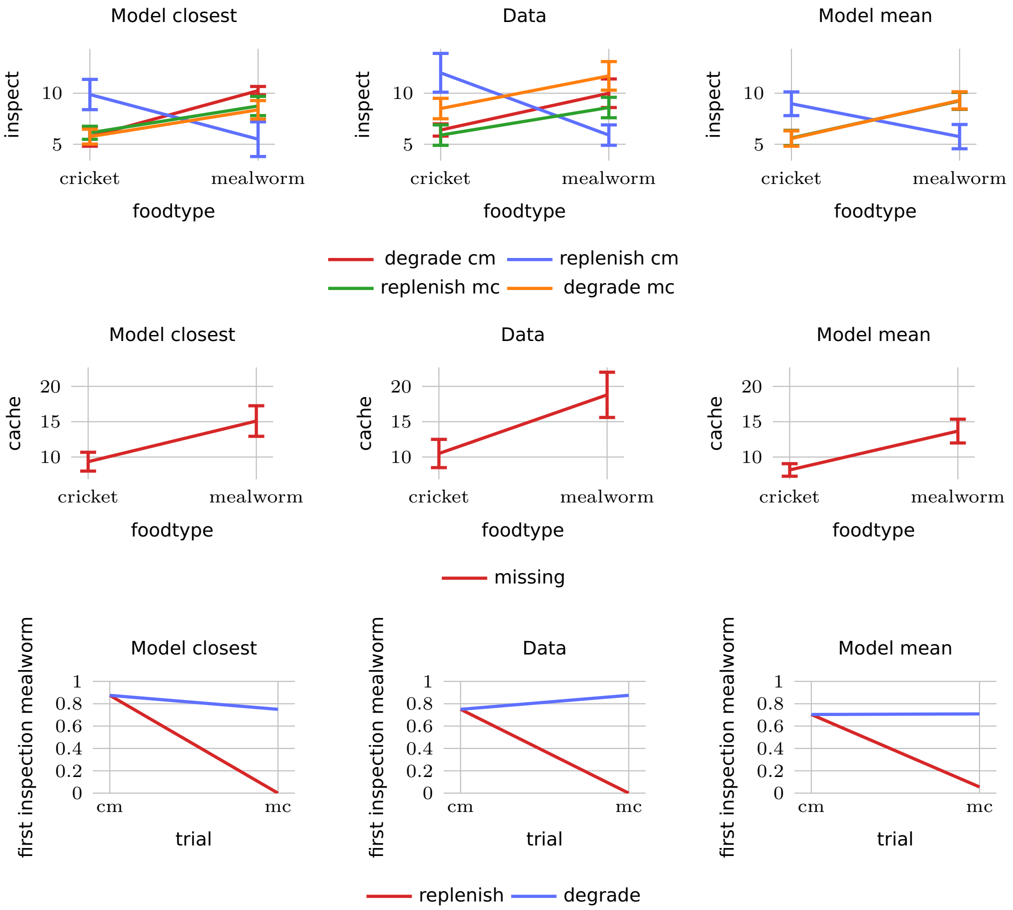

Figure S112: **Clayton01 exp3: Plastic Caching Model**

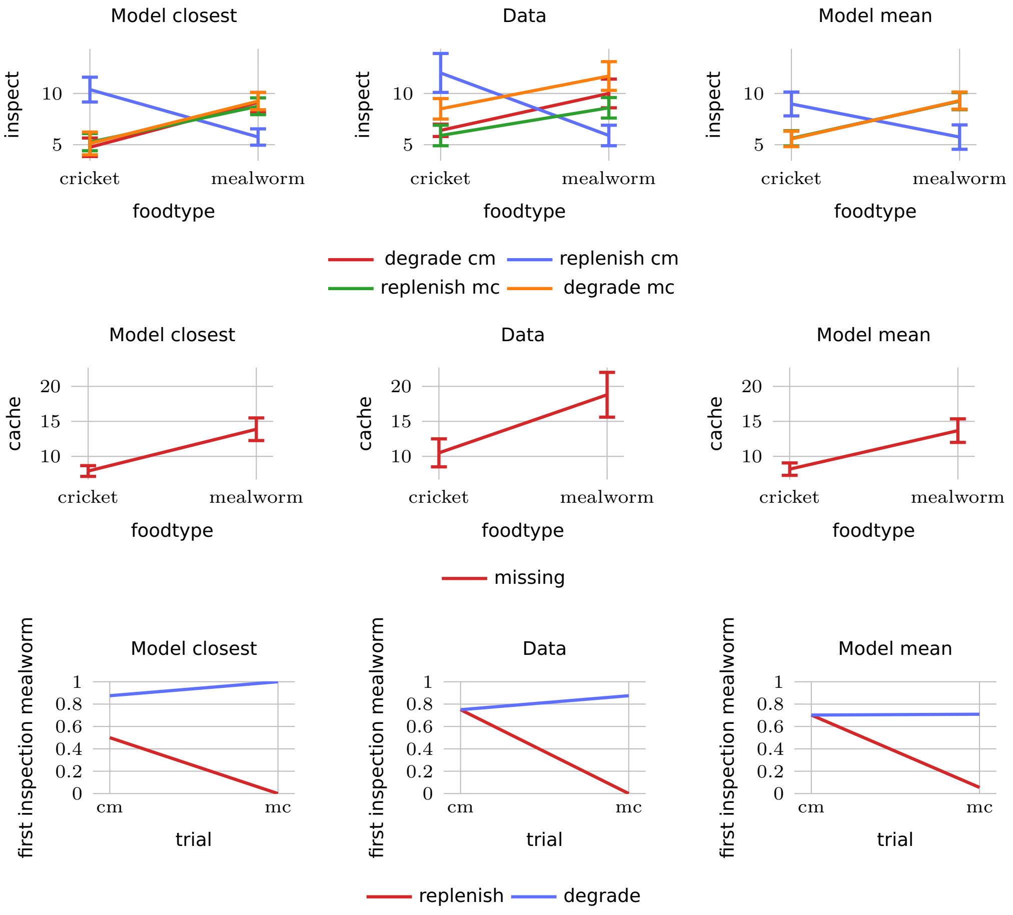

Figure S113: **Clayton01 exp3: Planning-By-Replay Model**

2.1.19 Clayton01 exp4

Published in [5].

Major Finding

The same search pattern as in experiments 1-3 is observed when the birds cache two types of food in the two sides of a caching tray at the same time: given the choice of a tray where the cached long ago they direct more searches to the unpreferred food and vice versa for short retention intervals.

Most Important Tests

inspect 4 (group & foodtype): $F(1, 14) = 3.51, p = 0.082$

inspect (group & foodtype & RI): $F(1, 14) = 9.53, p = 0.008$

inspect 28 (group & foodtype): $F(1, 14) = 10.45, p = 0.006$

Total Number of Statistics

14 p-values, 32 means or sems.

Figures

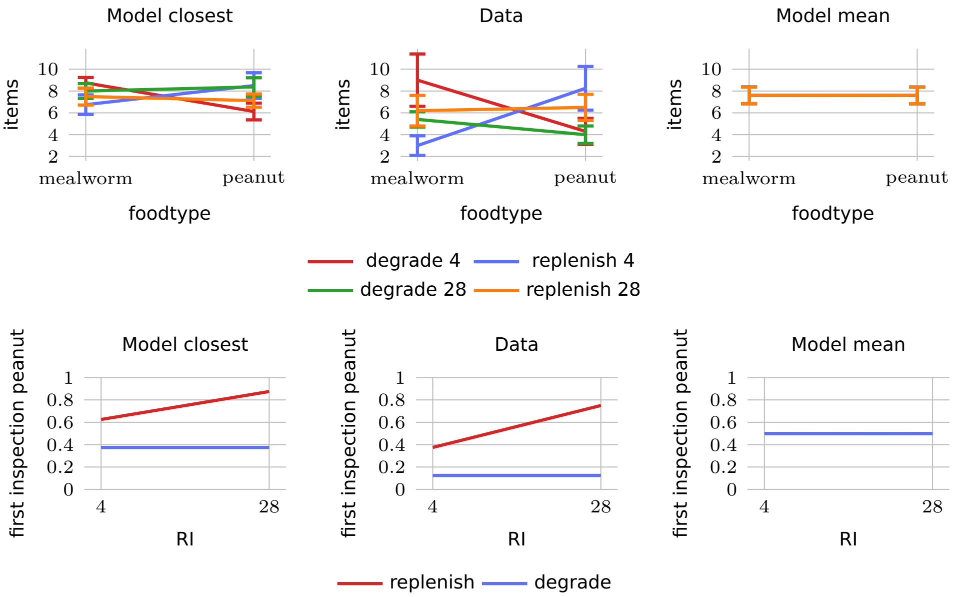

Figure S114: **Clayton01 exp4: No-Plasticity-No-Memory-No-Motivational-Control Model**

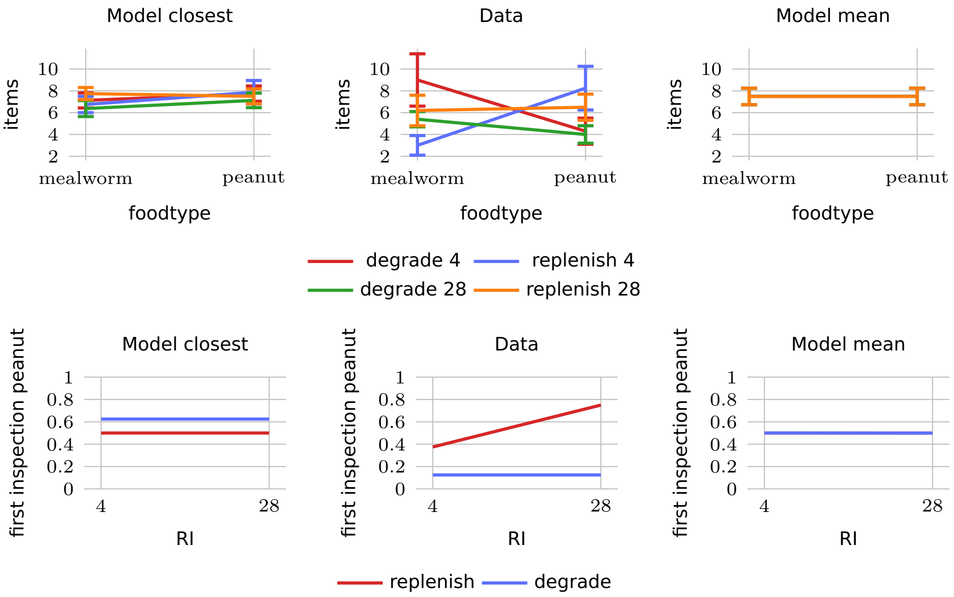

Figure S115: **Clayton01 exp4: No-Plasticity-No-Memory Model**

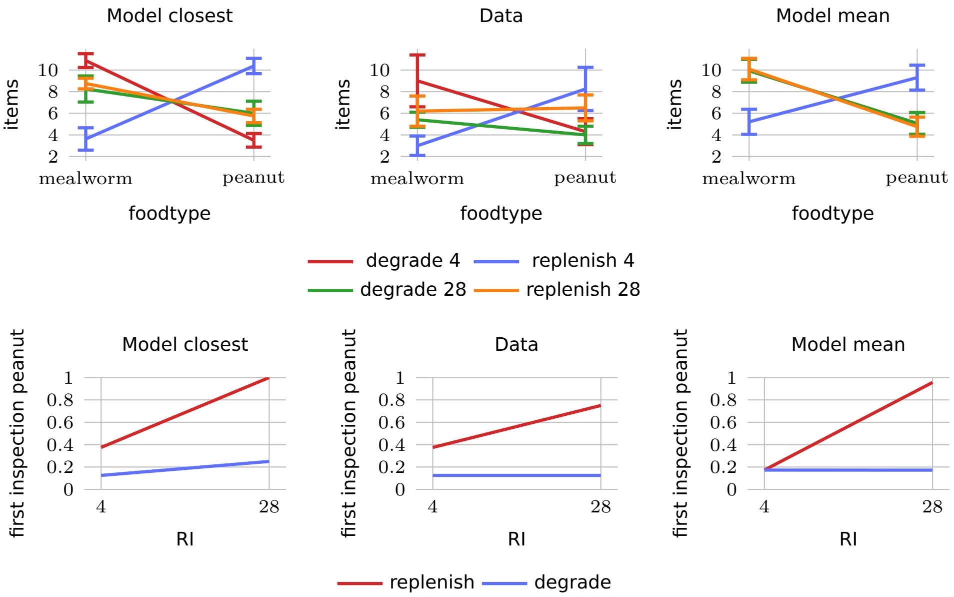

Figure S116: **Clayton01 exp4: No-Plasticity Model**

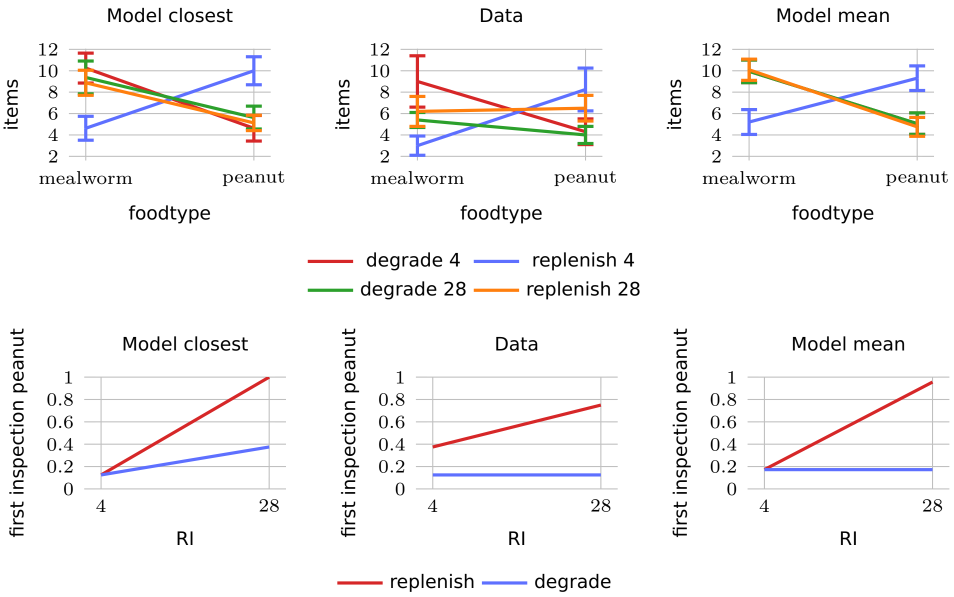

Figure S117: **Clayton01 exp4: Plastic Caching Model**

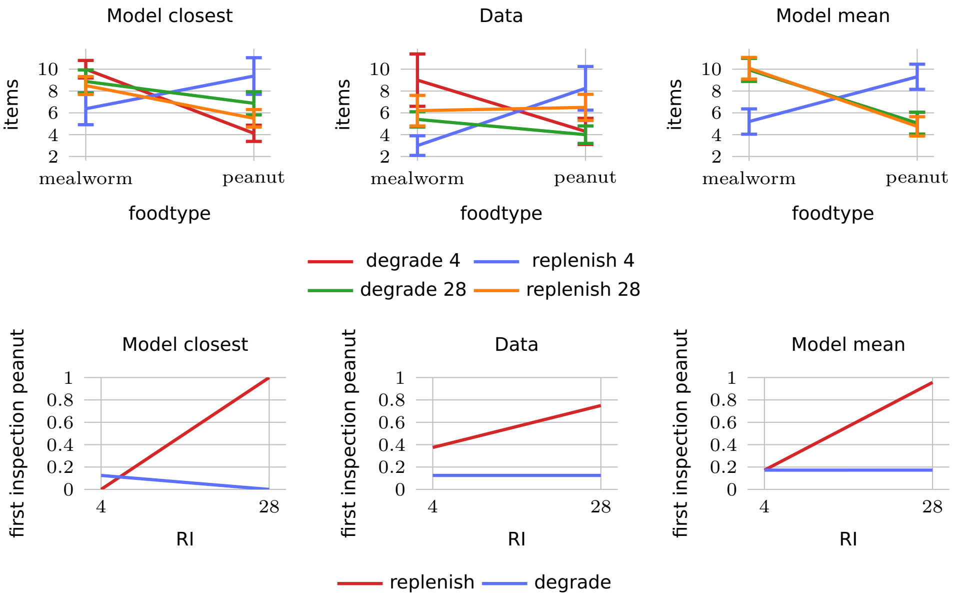

Figure S118: **Clayton01 exp4: Planning-By-Replay Model**

2.1.20 Clayton03 exp1

Published in [6].

Major Finding

Having experienced that crickets are fresh after 1 day but degraded after 4 days, the birds still search crickets after 2 or 3 days, but not after 5 days.

Most Important Tests

inspect RI 124 Clayton03 exp1 (group): $F(1, 11) > 7.01, p = 0.023$

inspect RI 76 Clayton03 exp1 (group): $F(1, 11) < 2.13, p = 0.17$

inspect RI 52 Clayton03 exp1 (group): $F(1, 11) < 2.13, p = 0.17$

Total Number of Statistics

16 p-values, 30 means or sems.

Figures

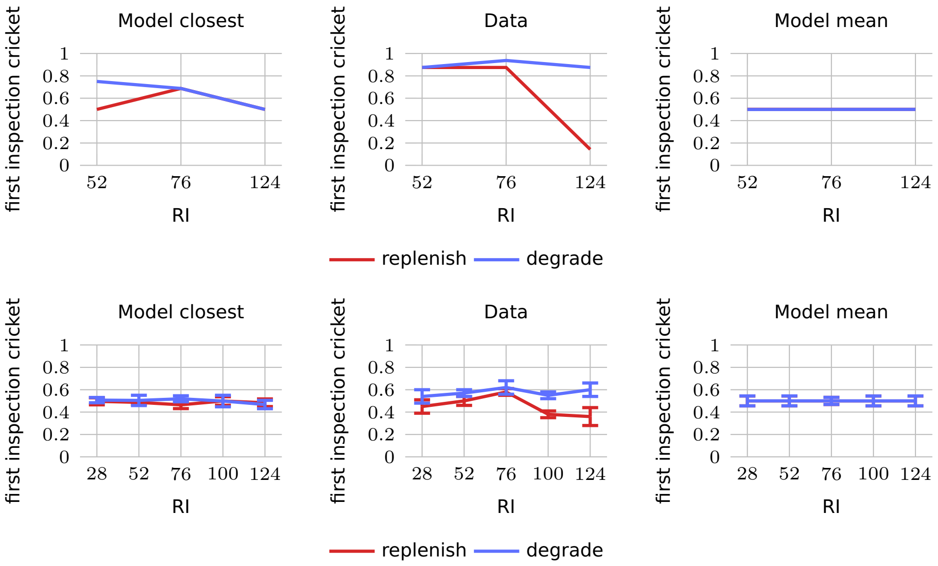

Figure S119: **Clayton03 exp1: No-Plasticity-No-Memory-No-Motivational-Control Model**

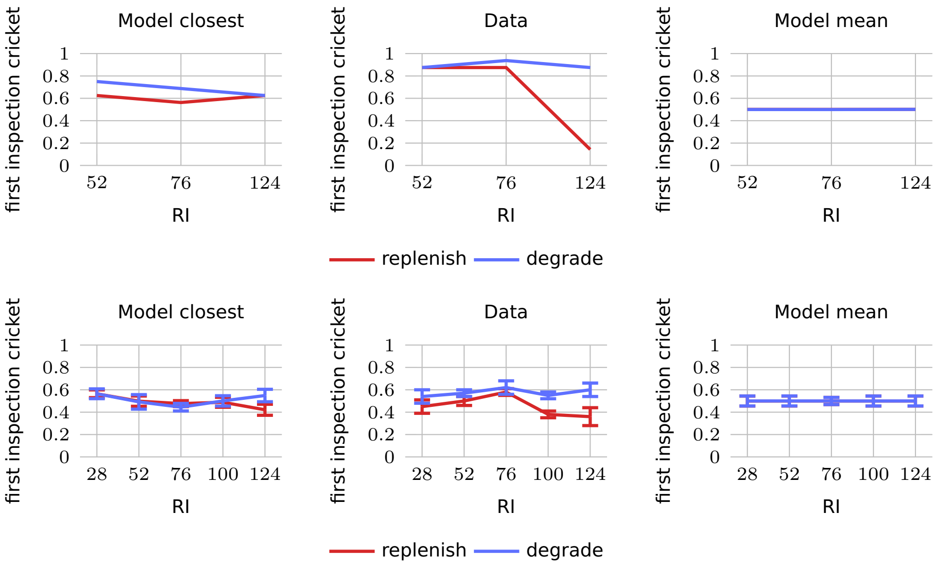

Figure S120: **Clayton03 exp1: No-Plasticity-No-Memory Model**

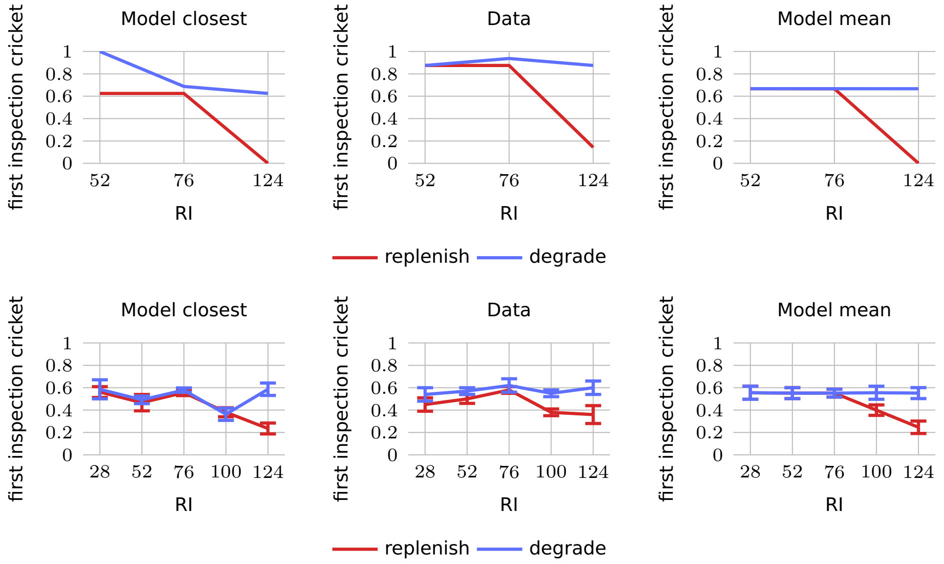

Figure S121: **Clayton03 exp1: No-Plasticity Model**

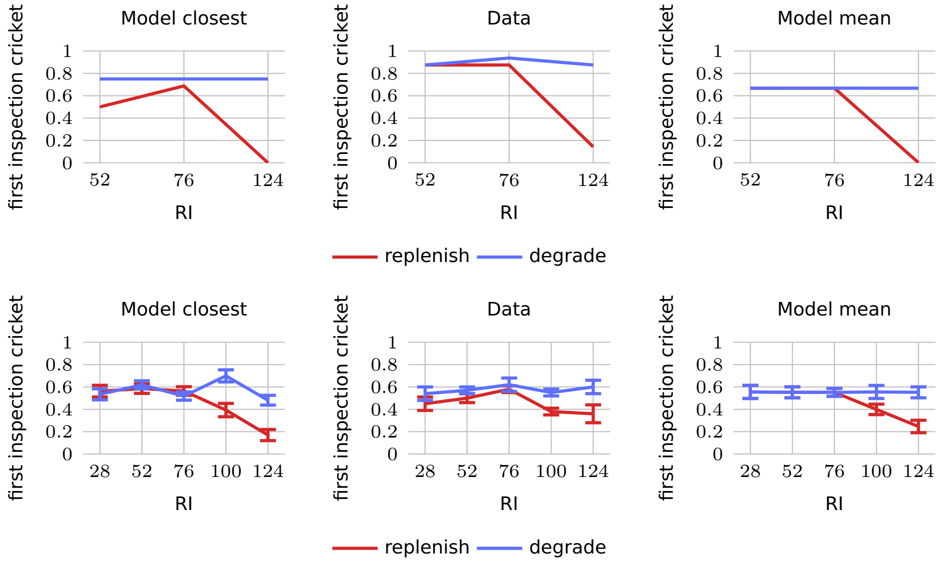

Figure S122: **Clayton03 exp1: Plastic Caching Model**

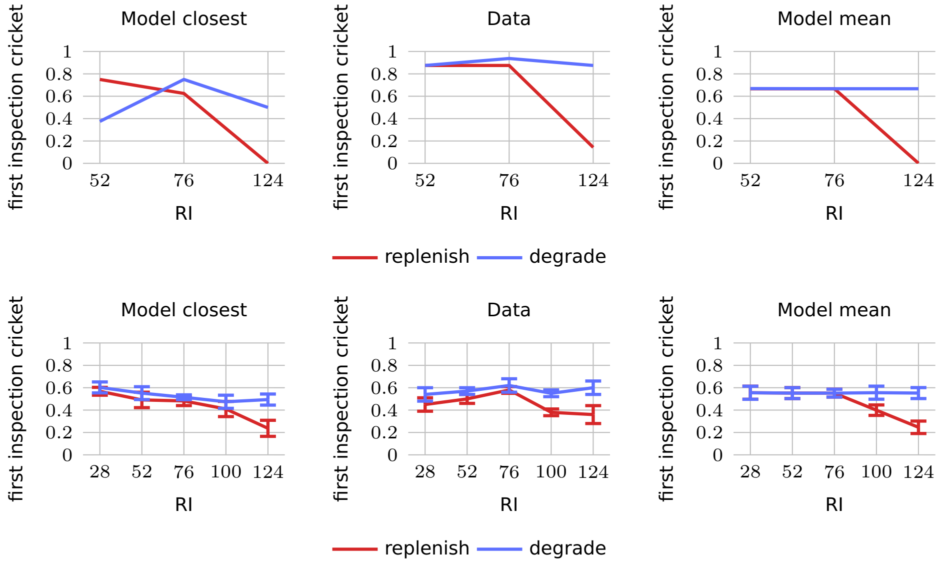

Figure S123: **Clayton03 exp1: Planning-By-Replay Model**

2.1.21 Clayton03 exp2

Published in [6].

Major Finding

Birds that experience degraded crickets after 3 days reduce searching for crickets after 3 day RIs even at sites where they cached prior to the unexpected experience of degraded crickets.

Comments

Some birds generalized to reduce searching for crickets after any retention interval (Replenish group, reversed, figure 4).

Most Important Tests

inspect degrade RI 76 (condition & foodtype): $F(1, 6) = 21.33, p = 0.0036$

inspect degrade (condition & foodtype & RI): $F(1, 6) = 7.44, p = 0.034$

inspect degrade RI 28 (condition & foodtype): $F(1, 6) < 1, p = 0.36$

Total Number of Statistics

25 p-values, 68 means or sems.

Figures

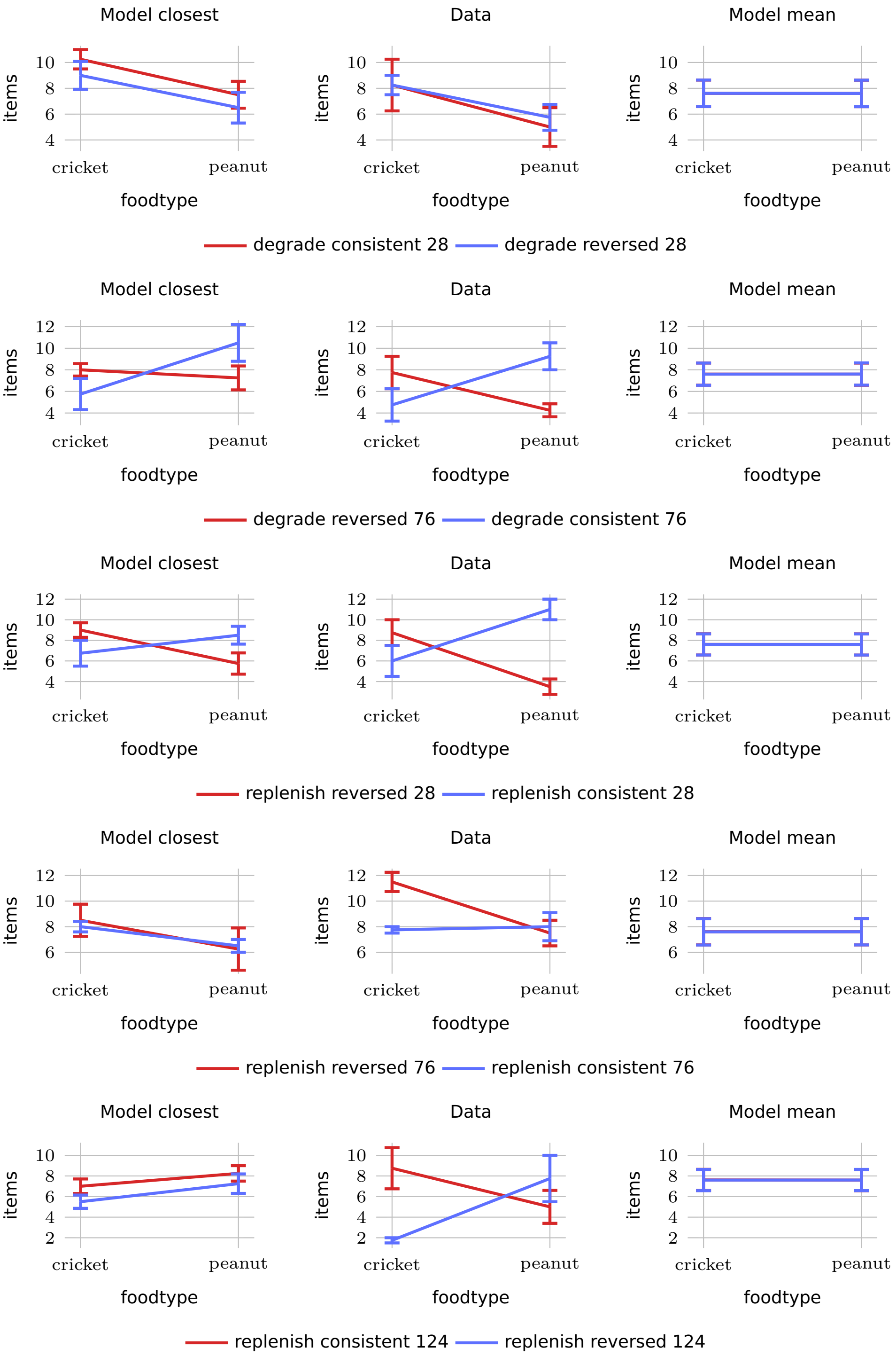

Figure S124: **Clayton03 exp2: No-Plasticity-No-Memory-No-Motivational-Control Model (part1)**

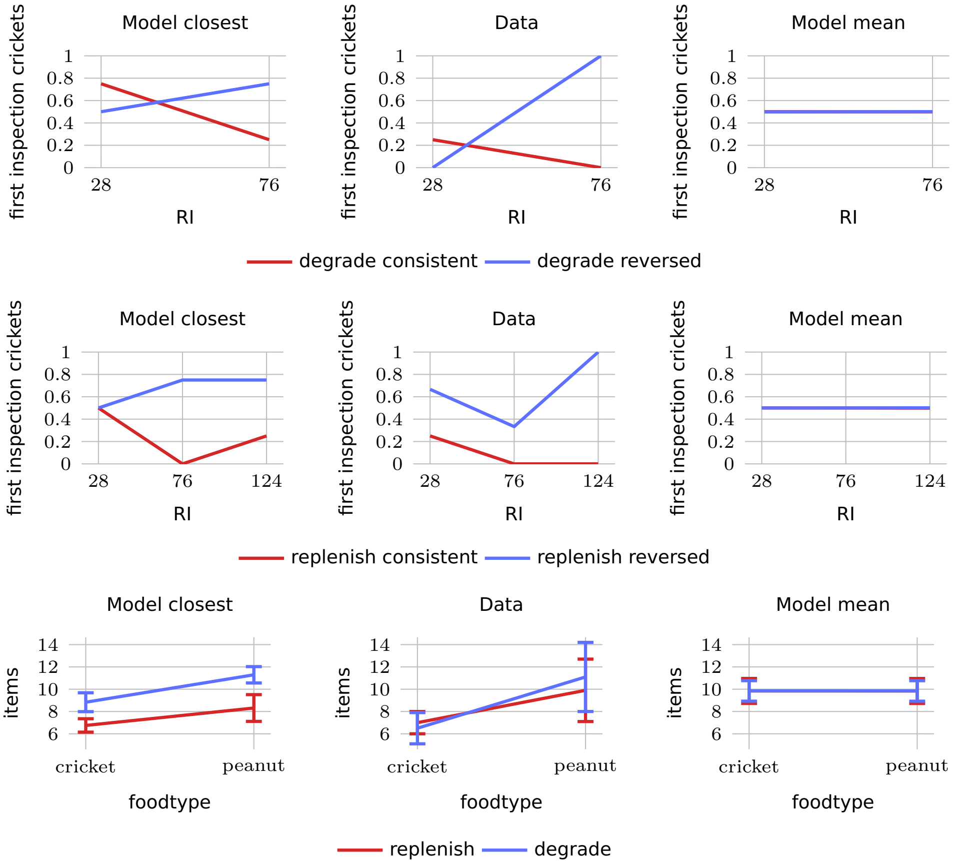

Figure S125: **Clayton03 exp2: No-Plasticity-No-Memory-No-Motivational-Control Model (part2)**

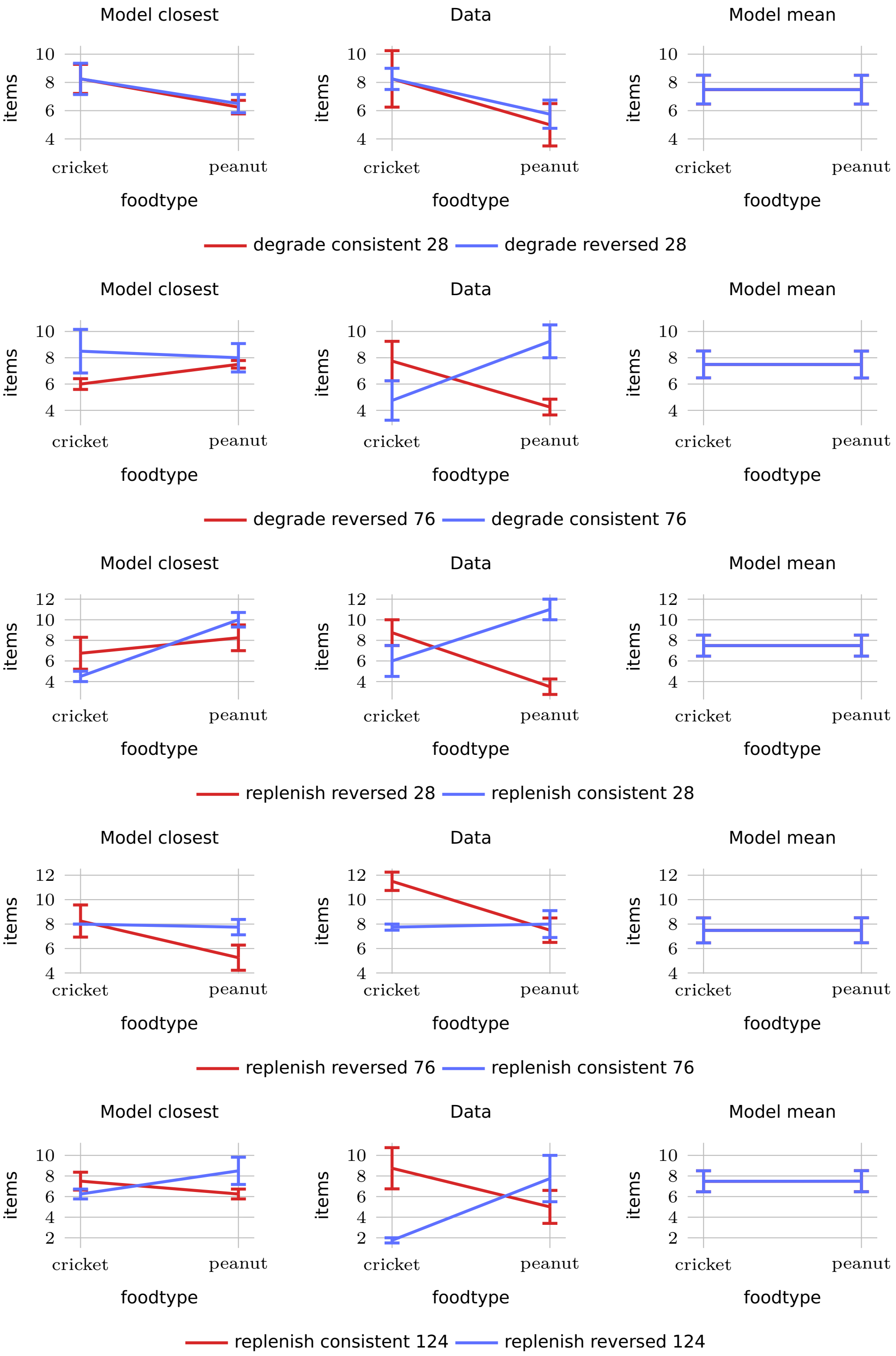

Figure S126: **Clayton03 exp2: No-Plasticity-No-Memory Model (part1)**

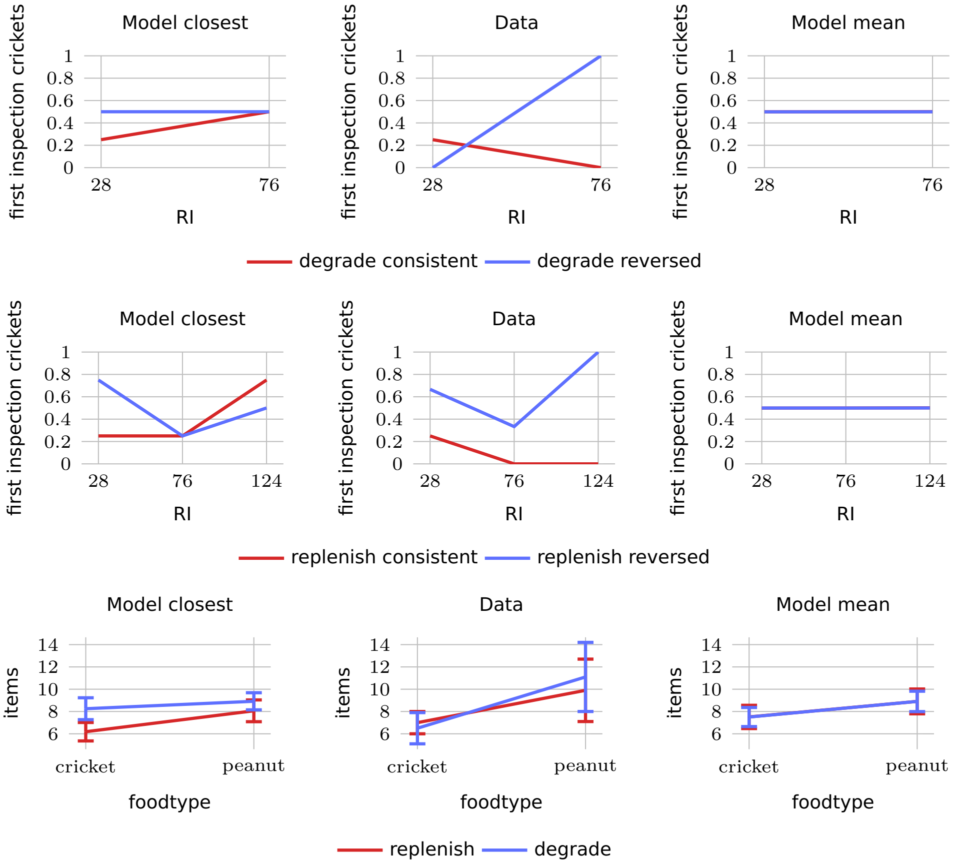

Figure S127: **Clayton03 exp2: No-Plasticity-No-Memory Model (part2)**

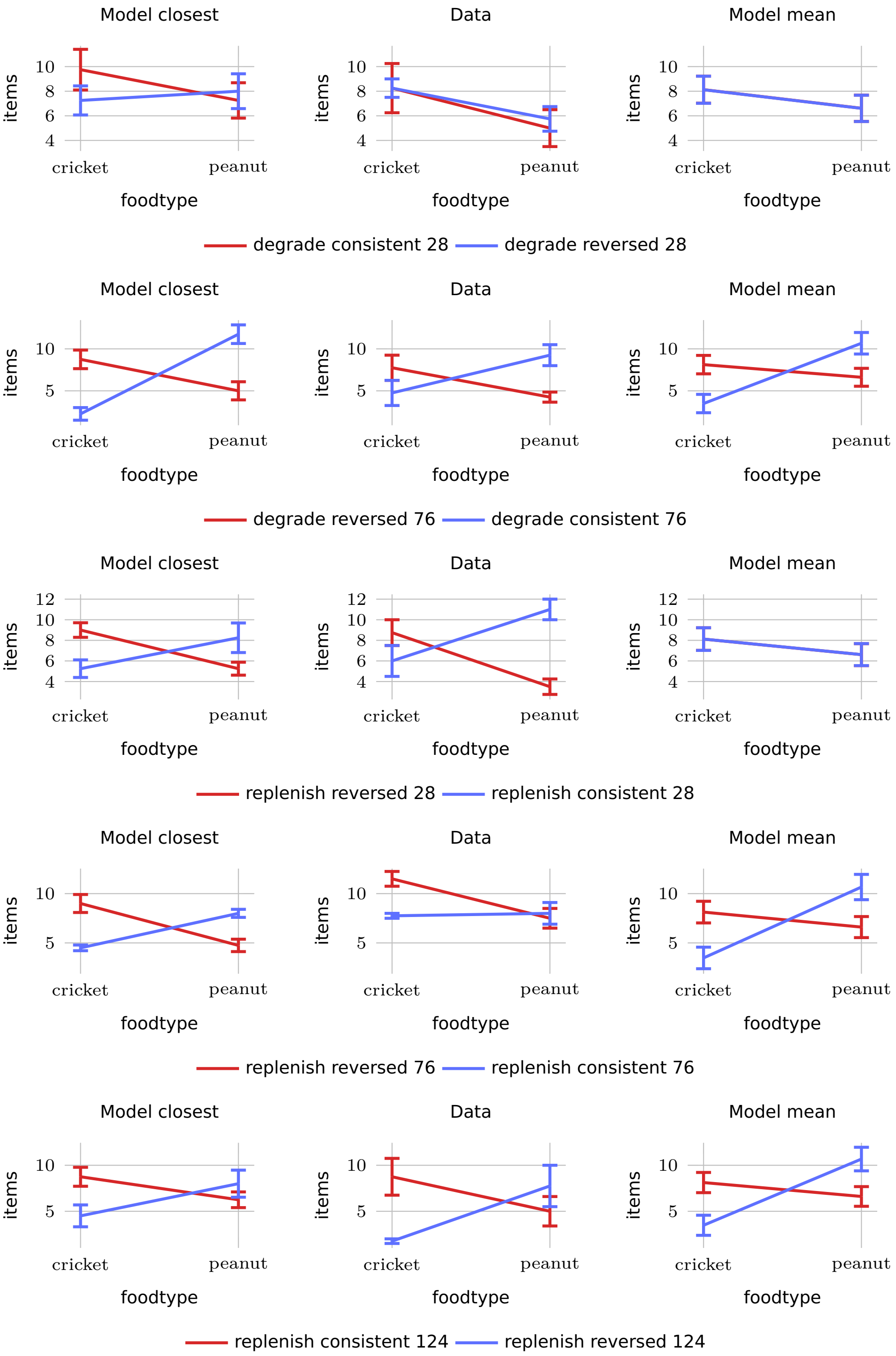

Figure S128: **Clayton03 exp2: No-Plasticity Model (part1)**

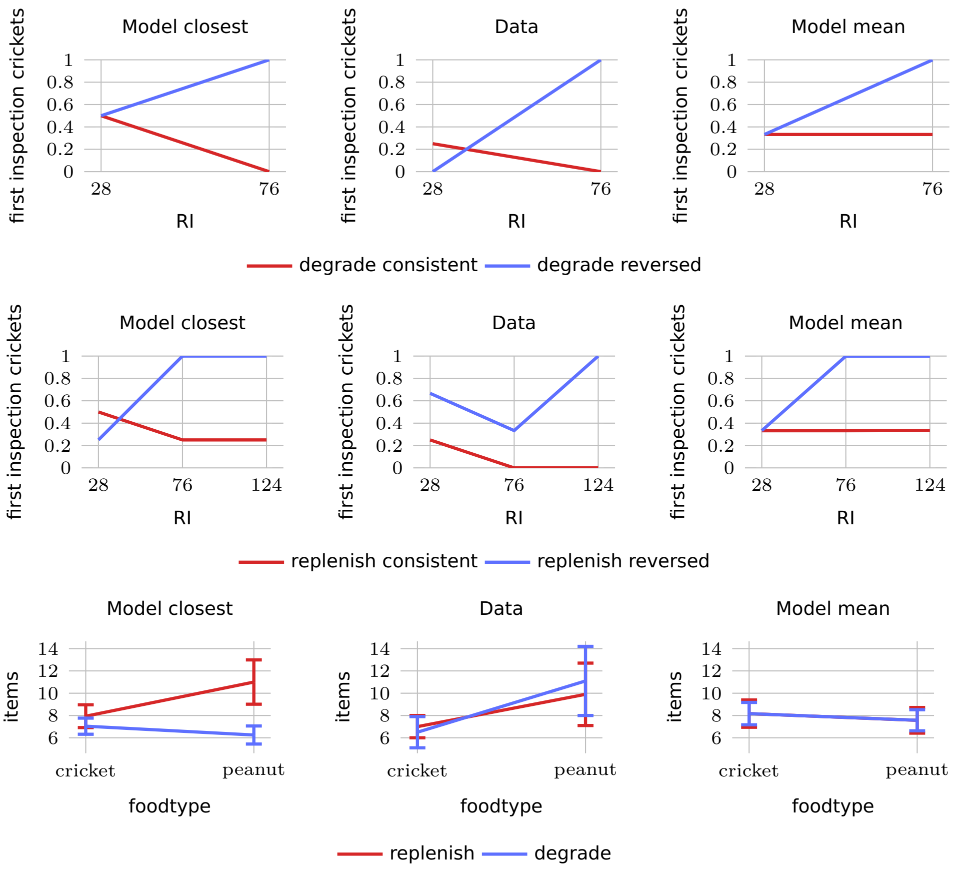

Figure S129: **Clayton03 exp2: No-Plasticity Model (part2)**

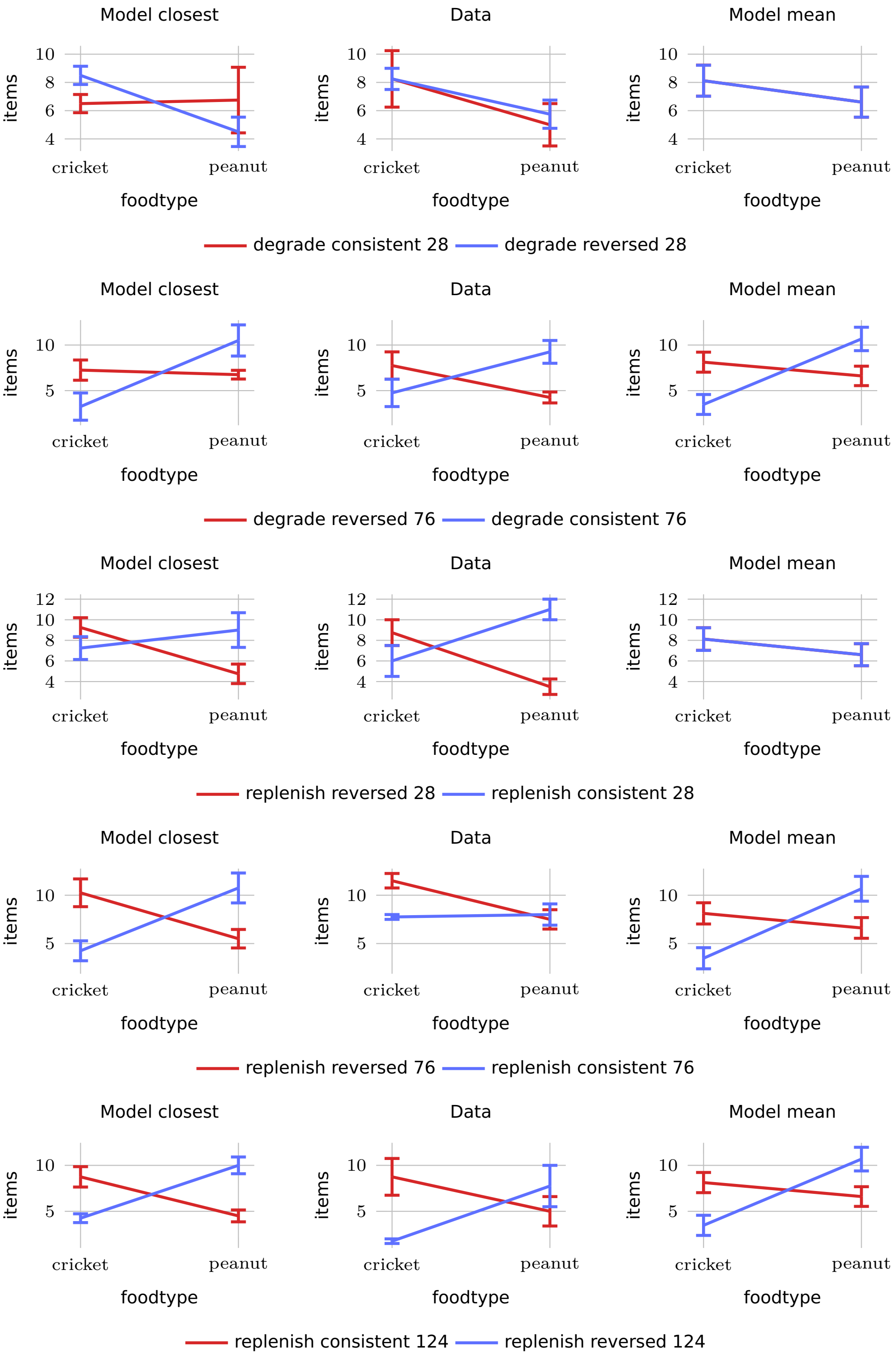

Figure S130: **Clayton03 exp2: Plastic Caching Model (part1)**

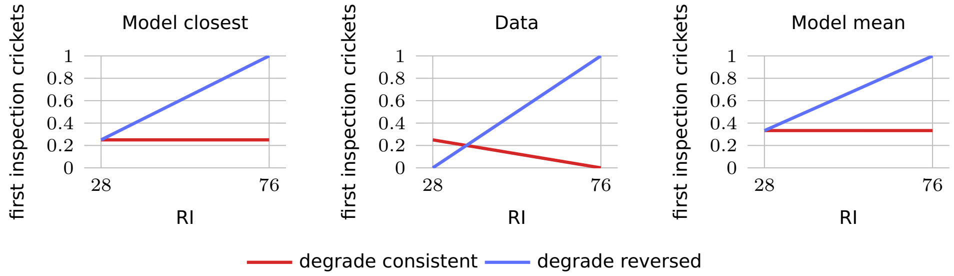

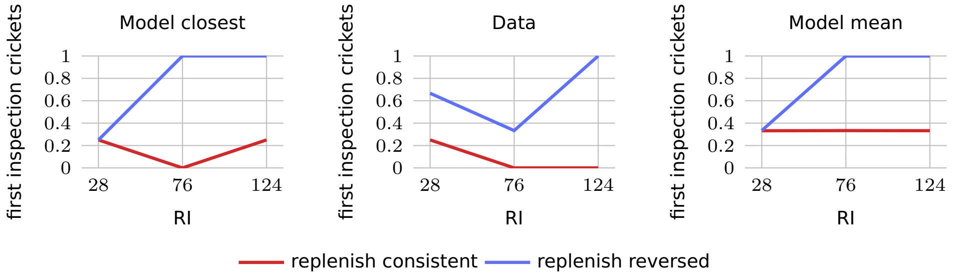

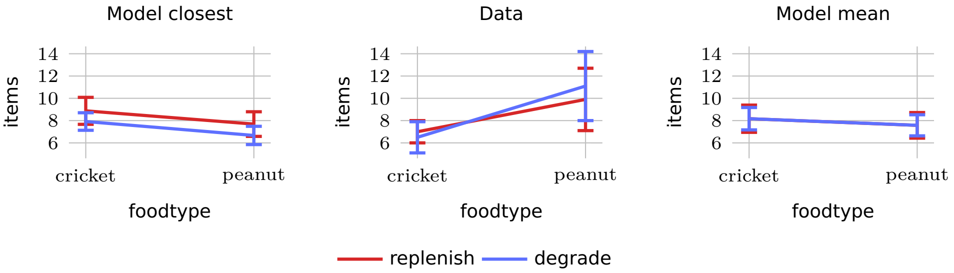

Figure S131: **Clayton03 exp2: Plastic Caching Model (part2)**

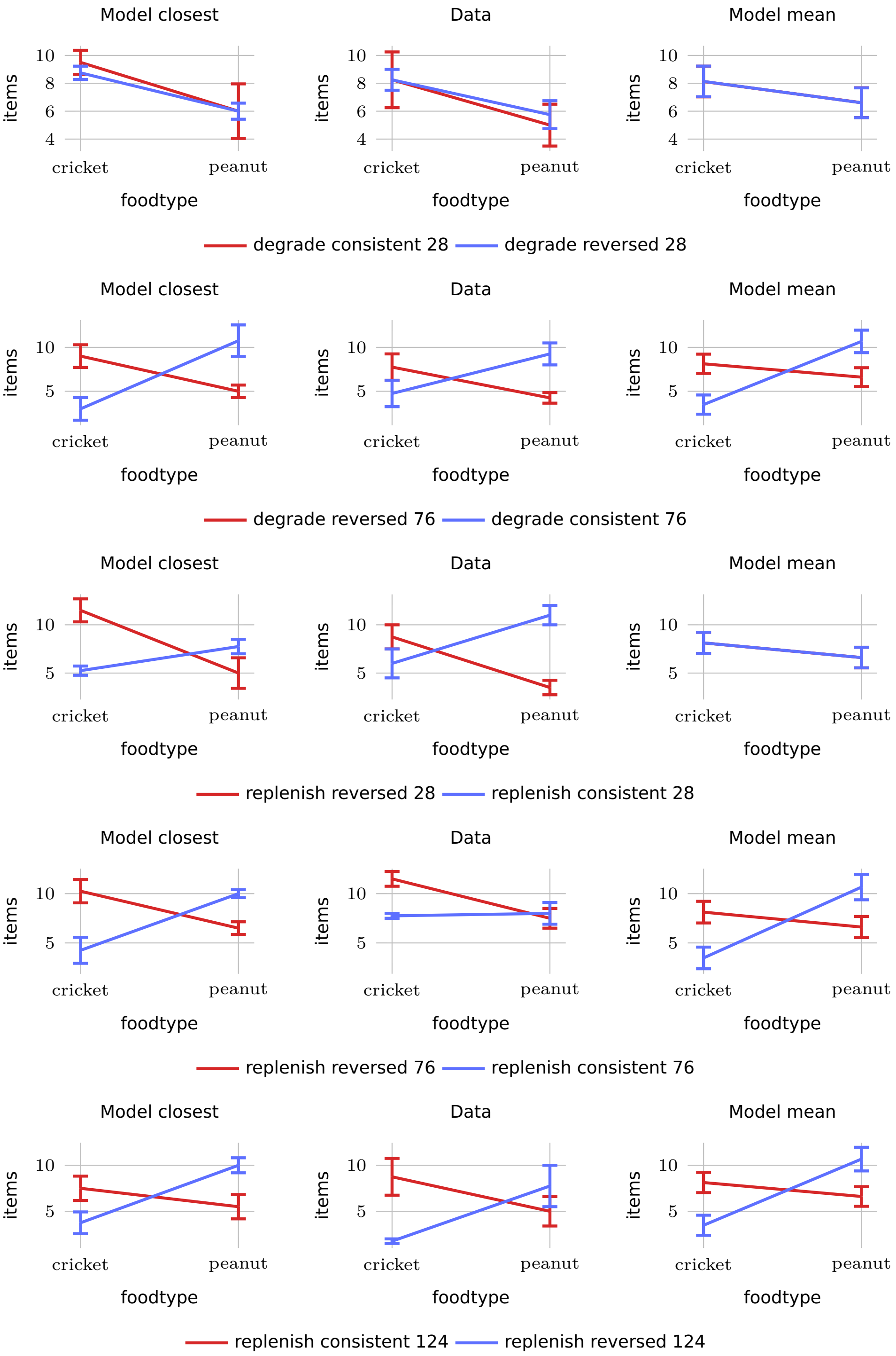

Figure S132: **Clayton03 exp2: Planning-By-Replay Model (part1)**

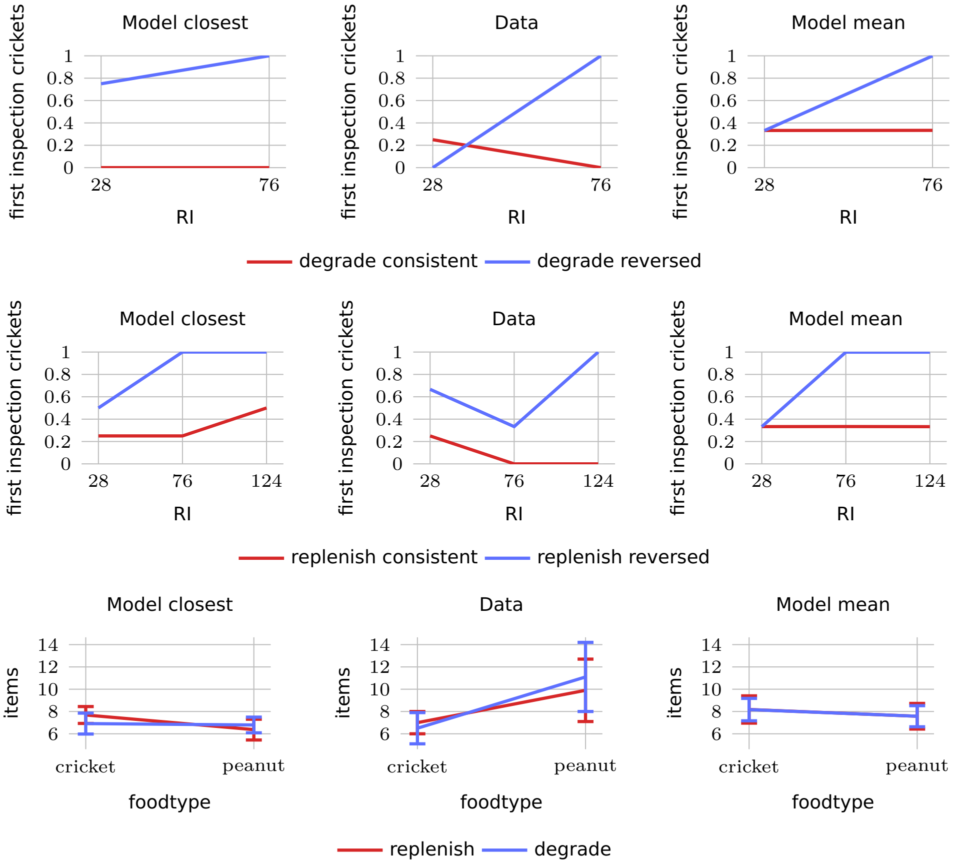

Figure S133: **Clayton03 exp2: Planning-By-Replay Model (part2)**

2.1.22 deKort05

Published in [7].

Major Finding

If palatability improves over time (ripening) the birds search more after the longer retention intervals than after the shorter retention interval and vice versa if the food items degrade over time.

Comments

For the Group x Retention Interval x Trail interaction we assume that the correct degree of freedoms would be 7,70.

Most Important Tests

first inspect trial 8 (group): $Chisq(3) = 9.08, p = 0.028$

inspect (group & RI): $F(1, 10) = 13.03, p = 0.0048$

inspect (group & RI & set): $F(1, 10) = 11.24, p = 0.0073$

Total Number of Statistics

3 p-values, 68 means or sems.

Figures

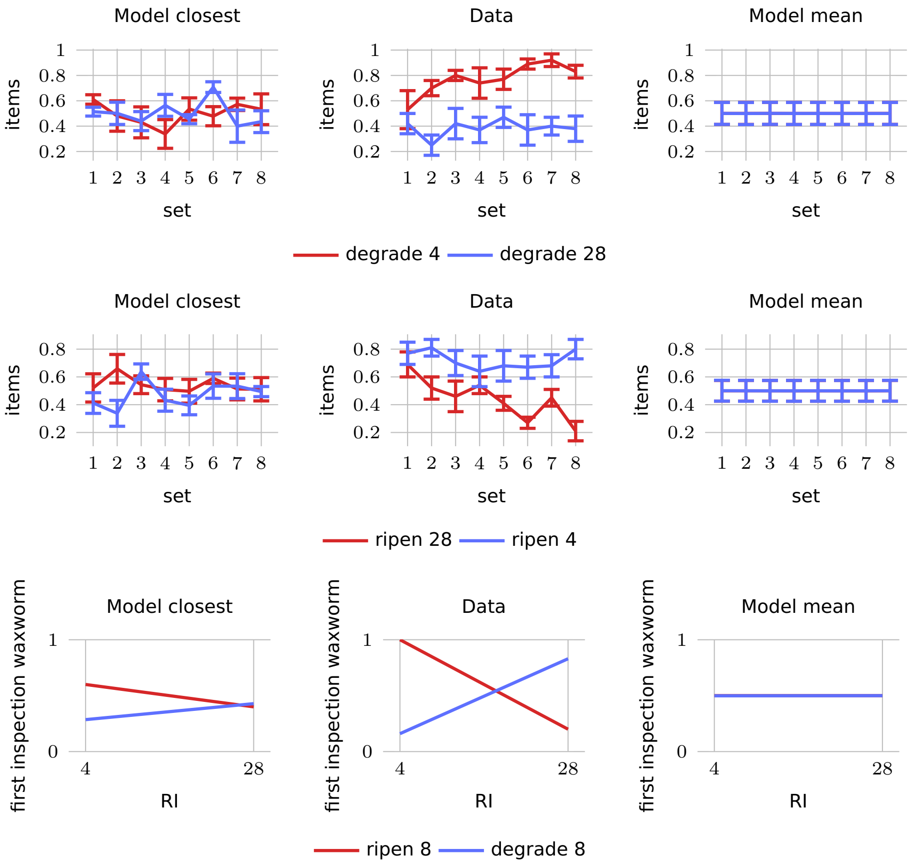

Figure S134: **deKort05: No-Plasticity-No-Memory-No-Motivational-Control Model**

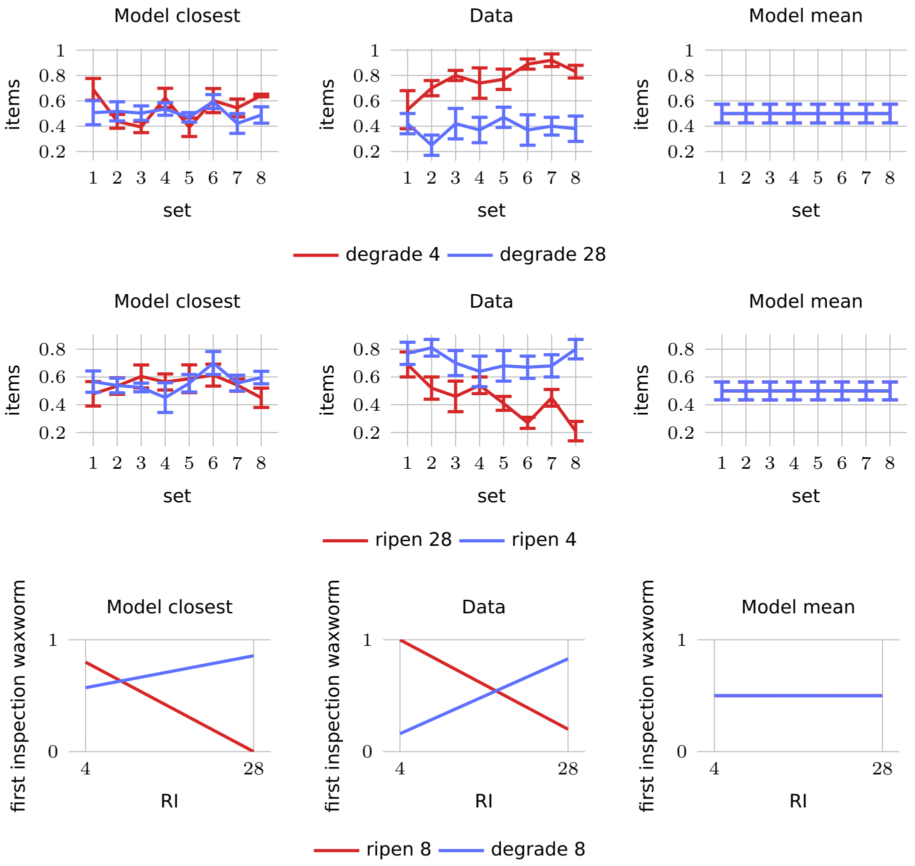

Figure S135: **deKort05: No-Plasticity-No-Memory Model**

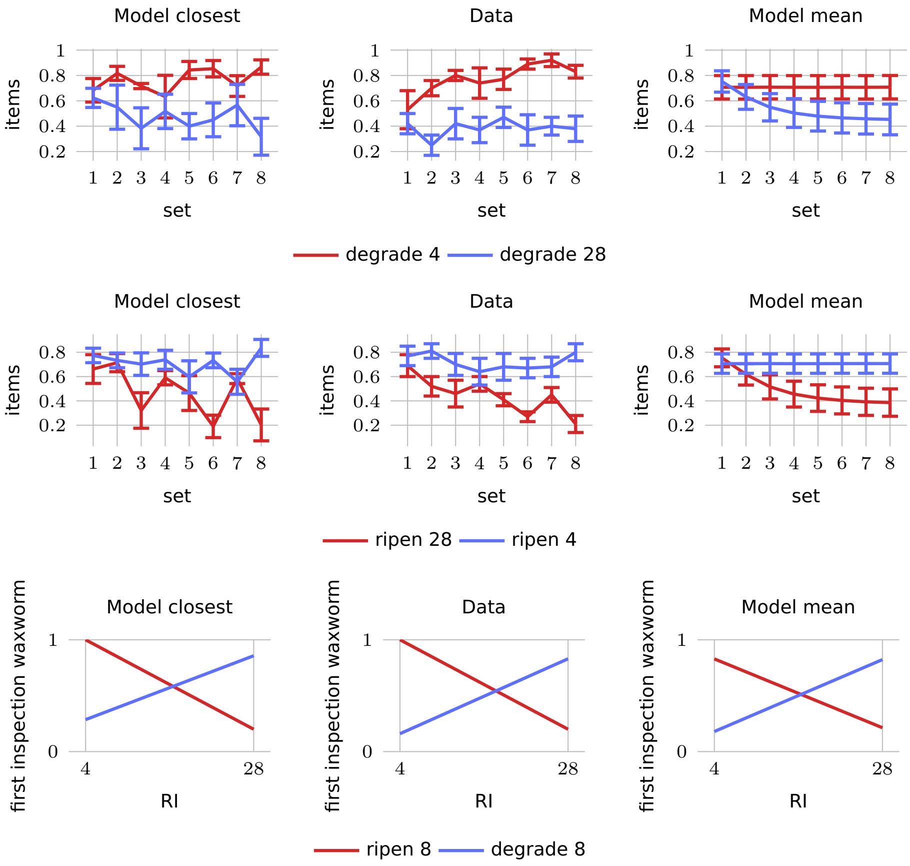

Figure S136: **deKort05: No-Plasticity Model**

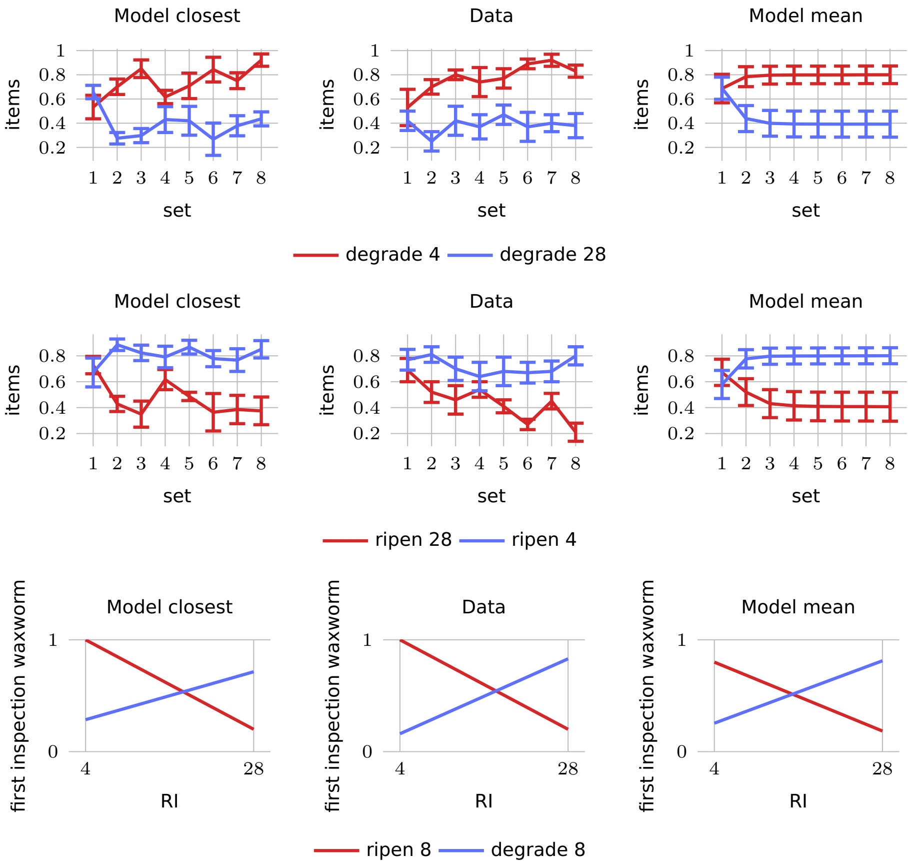

Figure S137: **deKort05: Plastic Caching Model**

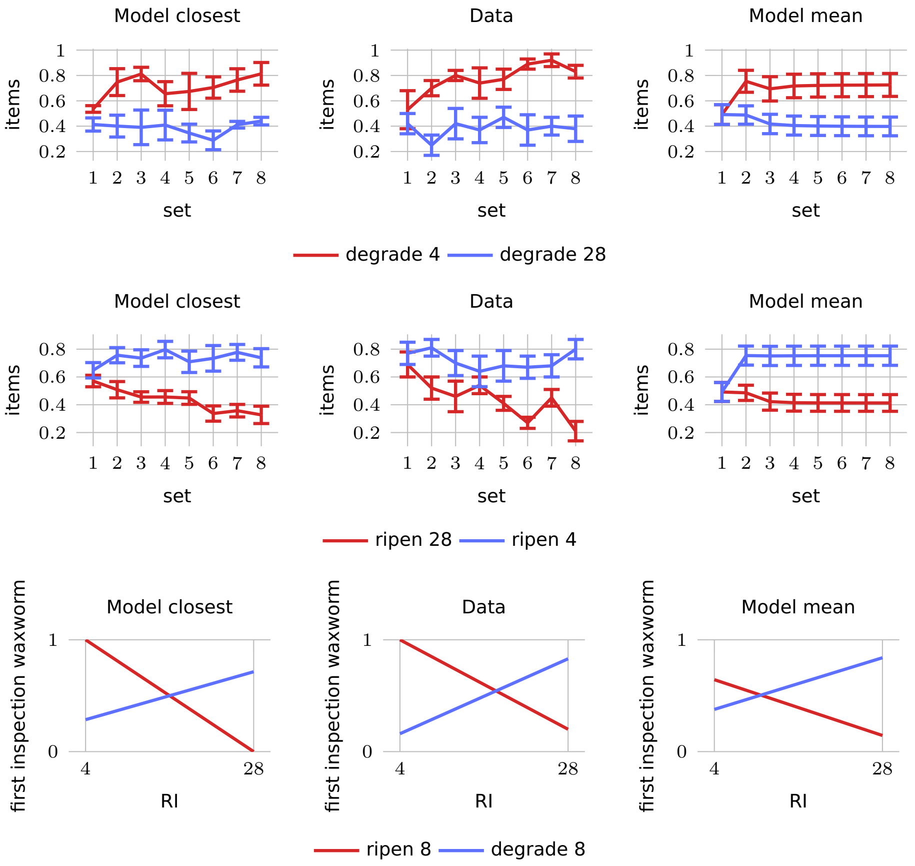

Figure S138: **deKort05: Planning-By-Replay Model**

2.1.23 Cheke11 specsat

Published in [12].

Major Finding

Birds showed specific satiety by eating and caching less of the pre-fed food than the non-prefed food.

Comments

This experiment is similar to Correia07 exp1 but with Eurasian jays instead of California scrub-jays.

Most Important Tests

cache (prefed & foodtype): $F(1, 3) = 10.45, p = 0.048$

overall (prefed & foodtype): $F(1, 3) = 12.4, p = 0.039$

eat (prefed & foodtype): $F(1, 3) = 9.8, p = 0.052$

Total Number of Statistics

3 p-values, 16 means or sems.

Figures

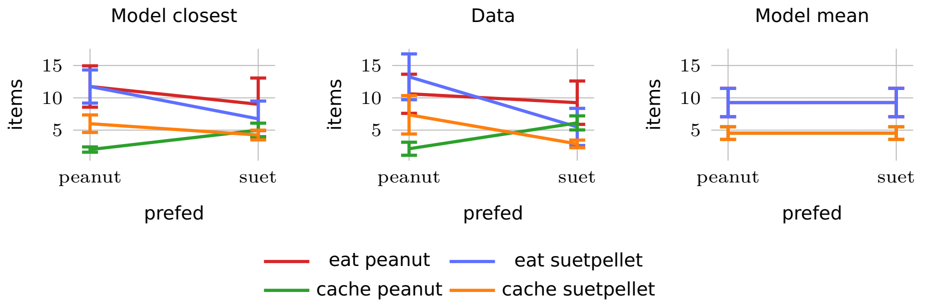

Figure S139: **Cheke11 specsat: No-Plasticity-No-Memory-No-Motivational-Control Model**

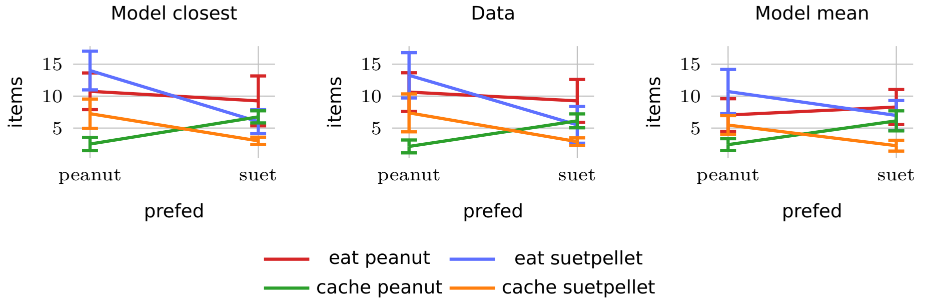

Figure S140: **Cheke11 specsat: No-Plasticity-No-Memory Model**

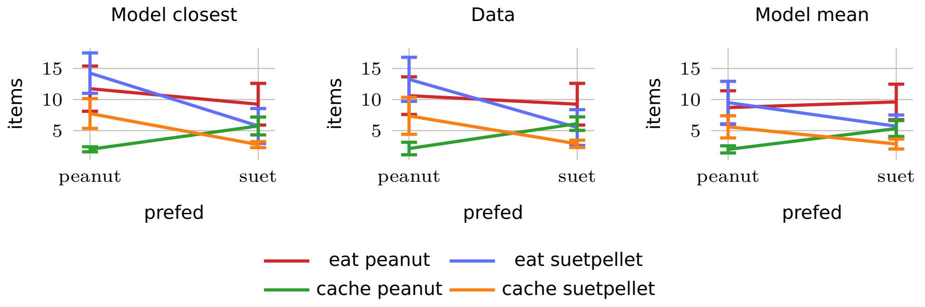

Figure S141: **Cheke11 specsat: No-Plasticity Model**

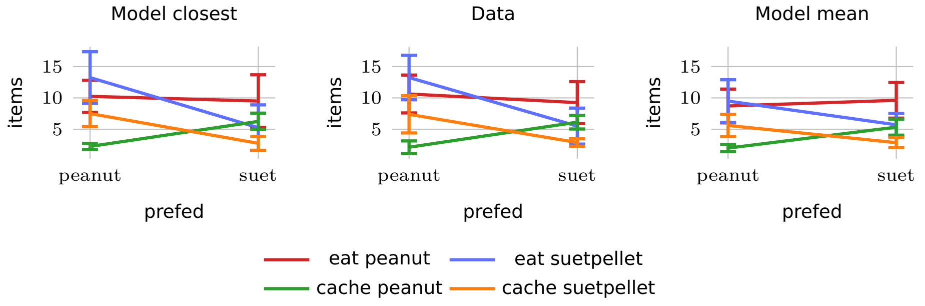

Figure S142: **Cheke11 specsat: Plastic Caching Model**

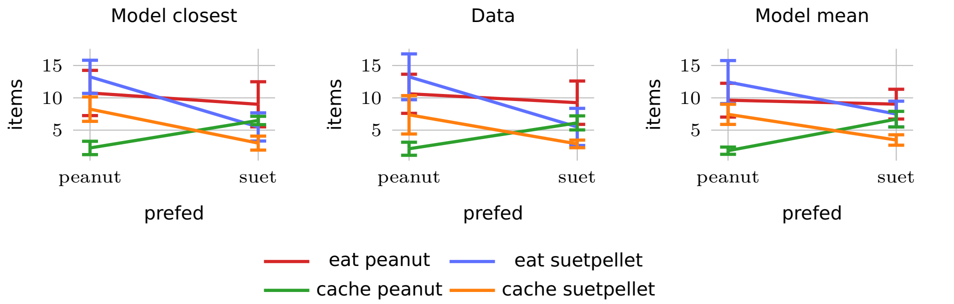

Figure S143: **Cheke11 specsat: Planning-By-Replay Model**

2.1.24 Correia07 exp1

Published in [11].

Major Finding

After eating to satiety a certain type of food, birds eat less of this type of food.

Most Important Tests

prefed pinenut (foodtype): $F(1, 10) = 6.96, p = 0.025$

prefed kibble (foodtype): $F(1, 10) = 5.12, p = 0.047$

interaction (prefed & foodtype): $F(3, 30) = 8.49, p = 0.00031$

Total Number of Statistics

5 p-values, 16 means or sems.

Figures

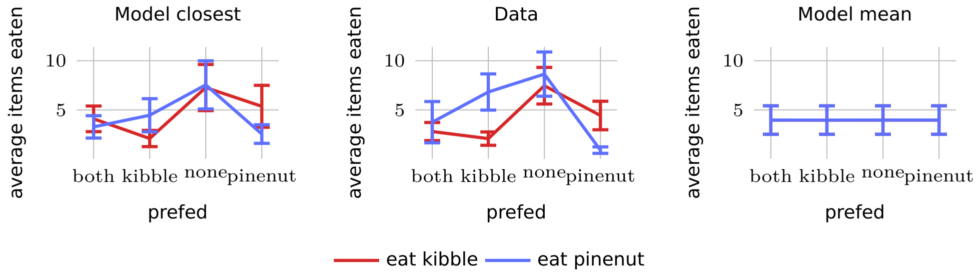

Figure S144: **Correia07 exp1: No-Plasticity-No-Memory-No-Motivational-Control Model**

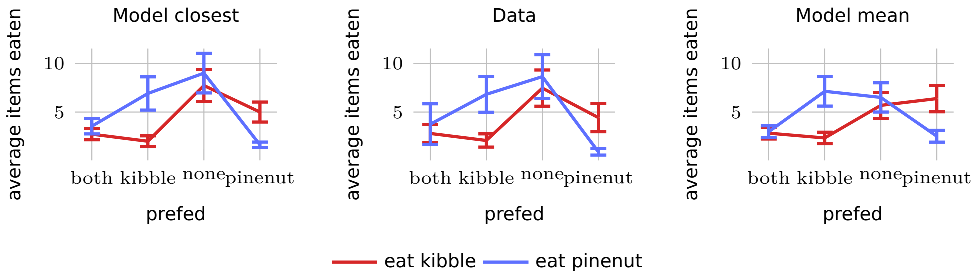

Figure S145: **Correia07 exp1: No-Plasticity-No-Memory Model**

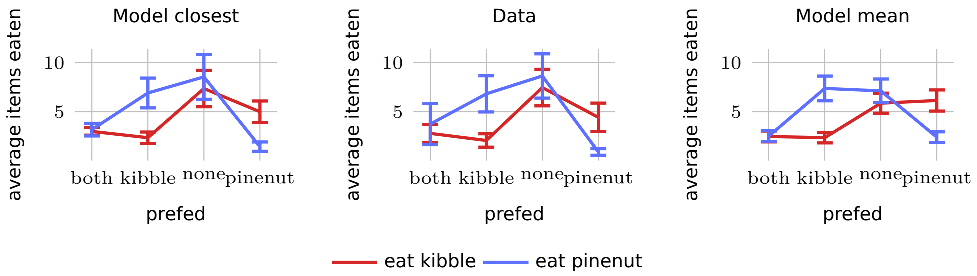

Figure S146: **Correia07 exp1: No-Plasticity Model**

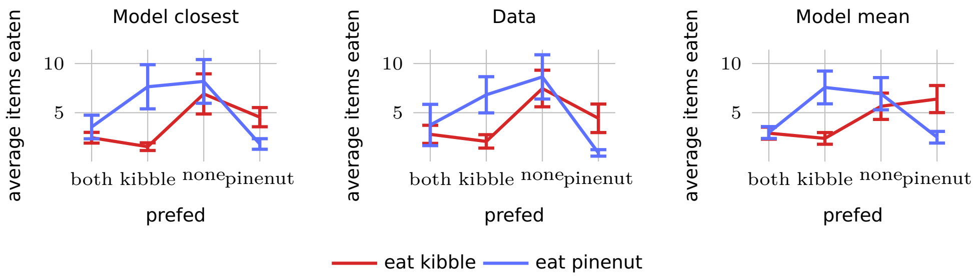

Figure S147: **Correia07 exp1: Plastic Caching Model**

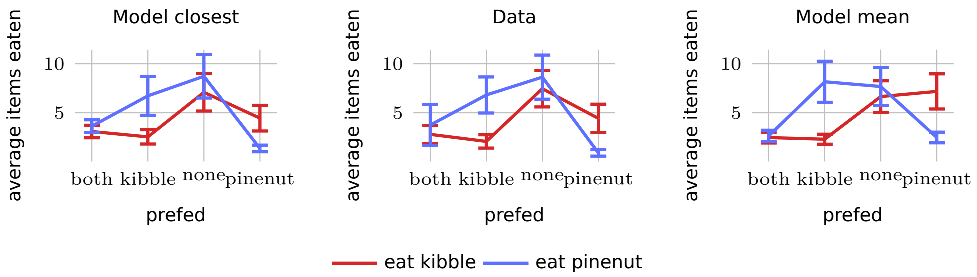

Figure S148: **Correia07 exp1: Planning-By-Replay Model**

2.1.25 Clayton99C exp1

Published in [24].

Major Finding

Neither prefeeding nor precaching alone had a major impact on the birds' subsequent propensity to cache peanuts or stones, but the combination of prefeeding and precaching in stage 1 significantly reduced the birds' propensity to cache in stage 2.

Most Important Tests

interactions (group): $F(3, 16) = 11.34, p = 0.00031$

interactions (foodtype): $F(1, 16) = 7.91, p = 0.013$

interactions (group & foodtype): $F(3, 16) = 12.64, p = 0.00017$

Total Number of Statistics

13 p-values, 30 means or sems.

Figures

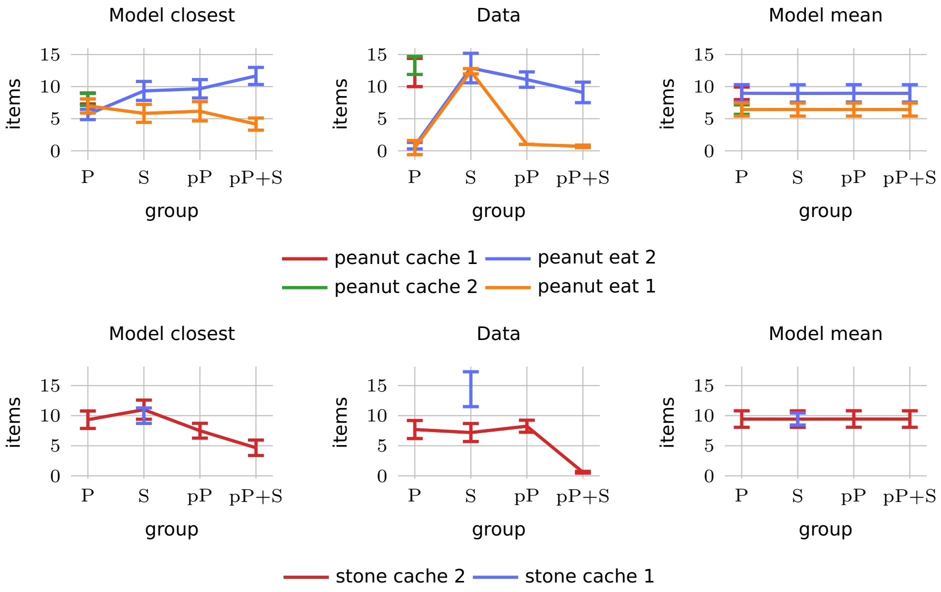

Figure S149: **Clayton99C exp1: No-Plasticity-No-Memory-No-Motivational-Control Model**

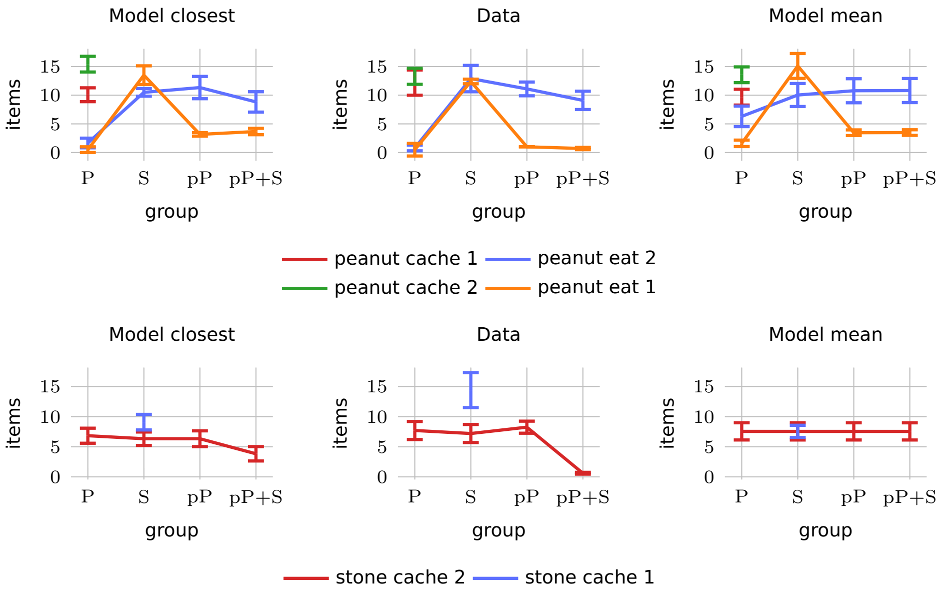

Figure S150: **Clayton99C exp1: No-Plasticity-No-Memory Model**

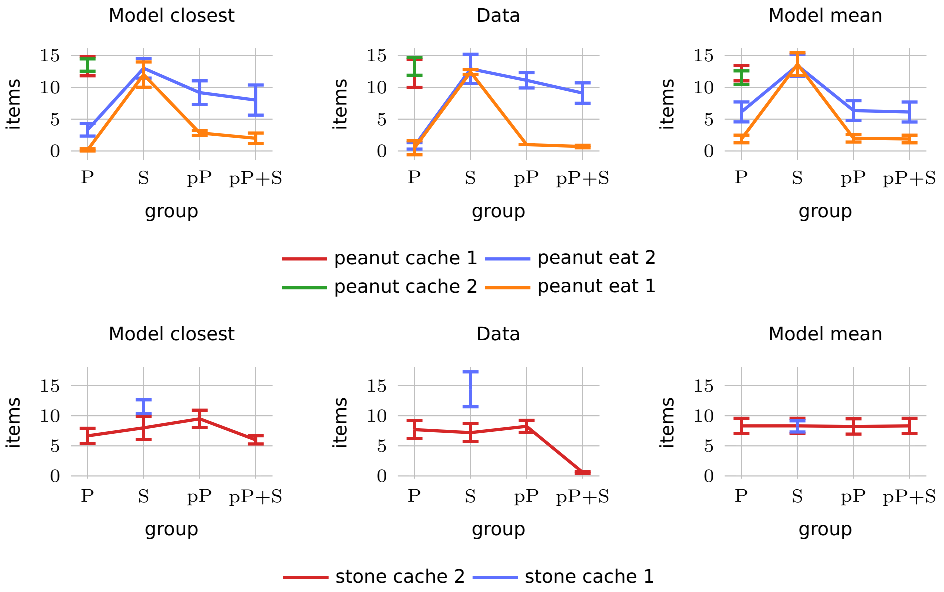

Figure S151: **Clayton99C exp1: No-Plasticity Model**

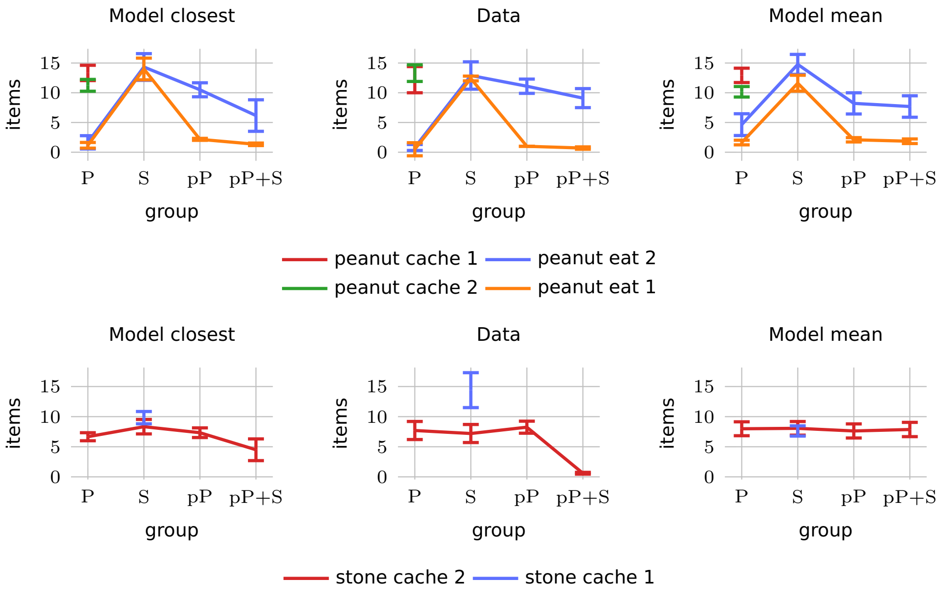

Figure S152: **Clayton99C exp1: Plastic Caching Model**

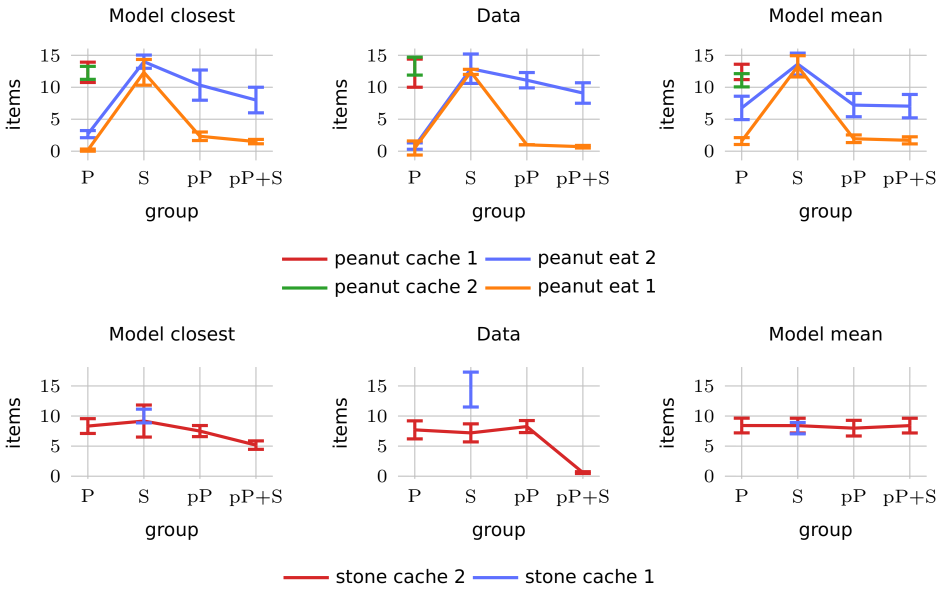

Figure S153: **Clayton99C exp1: Planning-By-Replay Model**

2.1.26 Clayton99C exp2

Published in [24].

Major Finding

Precaching and prefeeding produced a much greater reduction in subsequent caching and eating of the same food compared with a different food.

Most Important Tests

cache (stage): $F(1, 16) = 39.58, p = 1.1 \cdot 10^{-5}$

eat (stage): $F(1, 16) = 150.16, p = 1.5 \cdot 10^{-9}$

eat (group & stage): $F(1, 16) = 16.41, p = 0.00093$

Total Number of Statistics

38 p-values, 16 means or sems.

Figures

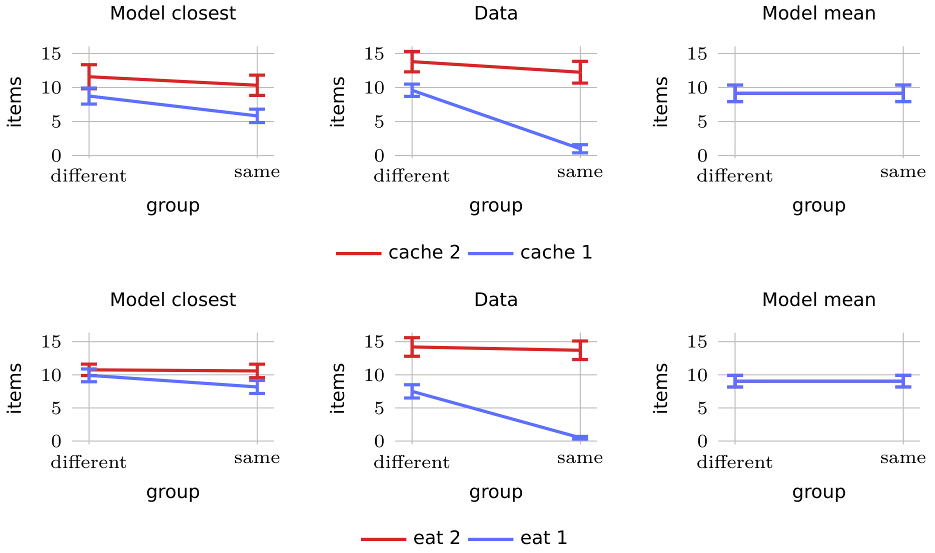

Figure S154: **Clayton99C exp2: No-Plasticity-No-Memory-No-Motivational-Control Model**

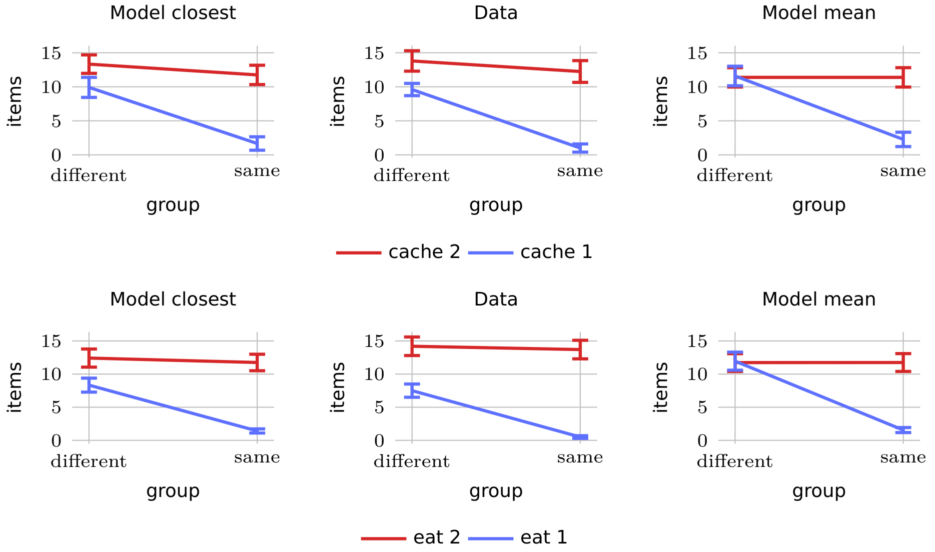

Figure S155: **Clayton99C exp2: No-Plasticity-No-Memory Model**

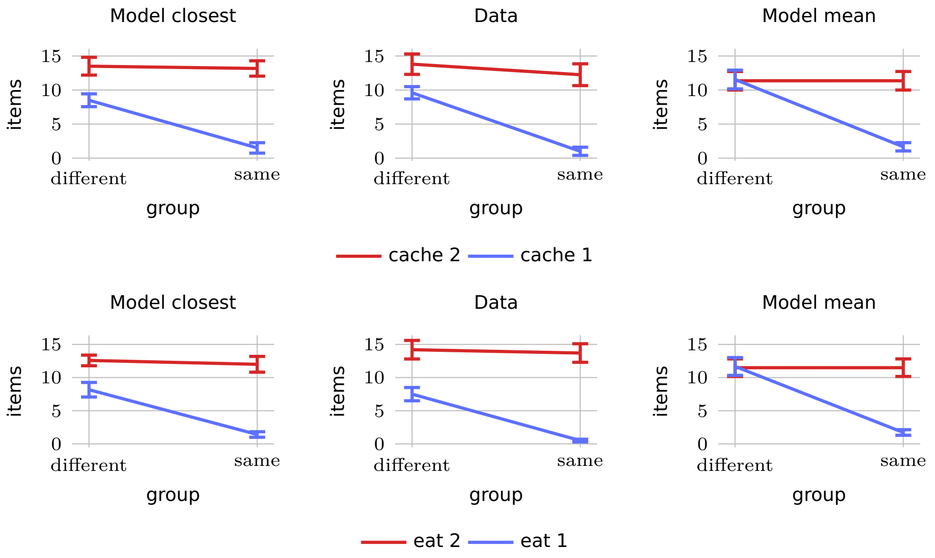

Figure S156: **Clayton99C exp2: No-Plasticity Model**

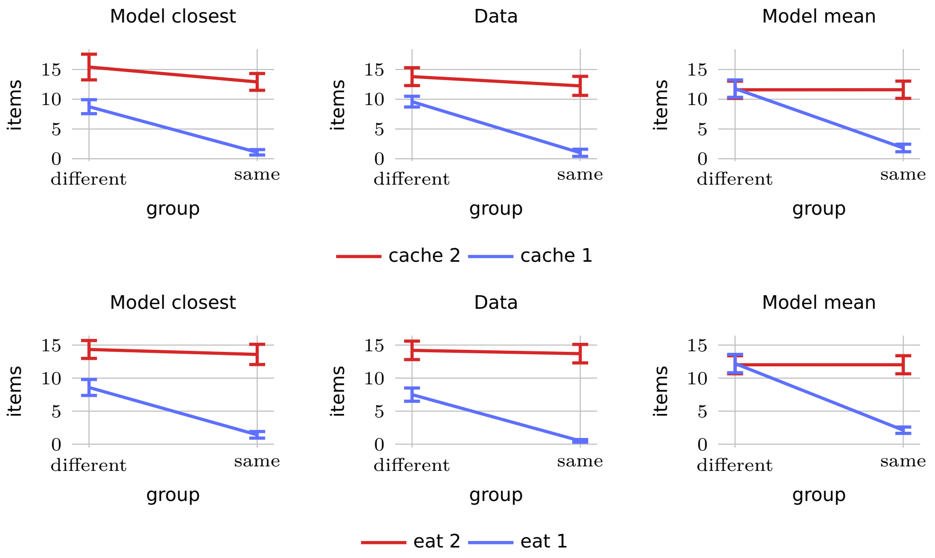

Figure S157: **Clayton99C exp2: Plastic Caching Model**

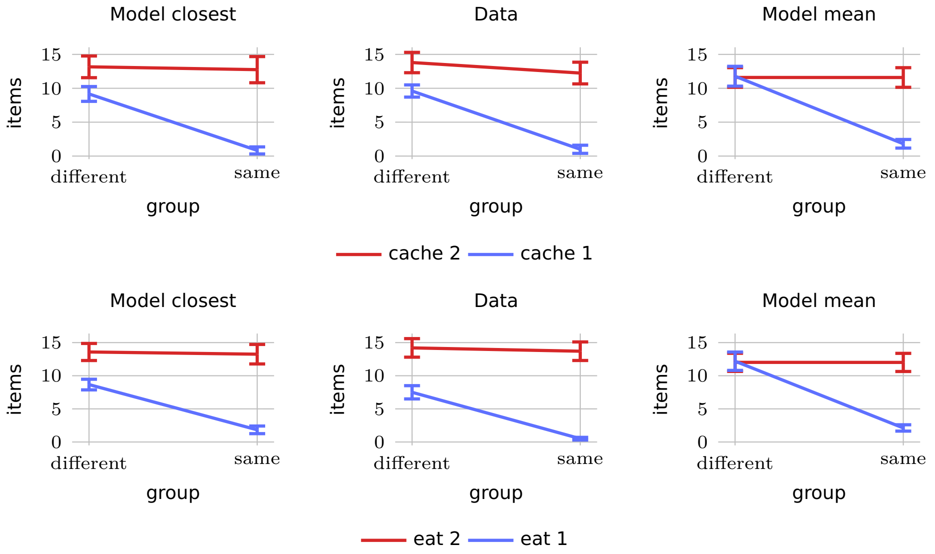

Figure S158: **Clayton99C exp2: Planning-By-Replay Model**

2.1.27 Clayton99C exp3

Published in [24].

Major Finding

Prefeeding of powdered, non-cacheable food leads to a strong reduction in eating of the same food, a slight reduction in eating different food and a small reduction in caching same food.

Comments

In constrast to experiment 1, pre-feeding powdered peanuts lead to a reduction in caching peanuts but not to a reduction in caching the other food.

Most Important Tests

cache (group): $F(2, 18) = 11.95, p = 0.0005$

within-subject prefeeding (group): $F(1, 22) = 2.33^2, p = 0.029$

eat (group): $F(2, 18) = 122., p = 3.4 \cdot 10^{-11}$

Total Number of Statistics

7 p-values, 16 means or sems.

Figures

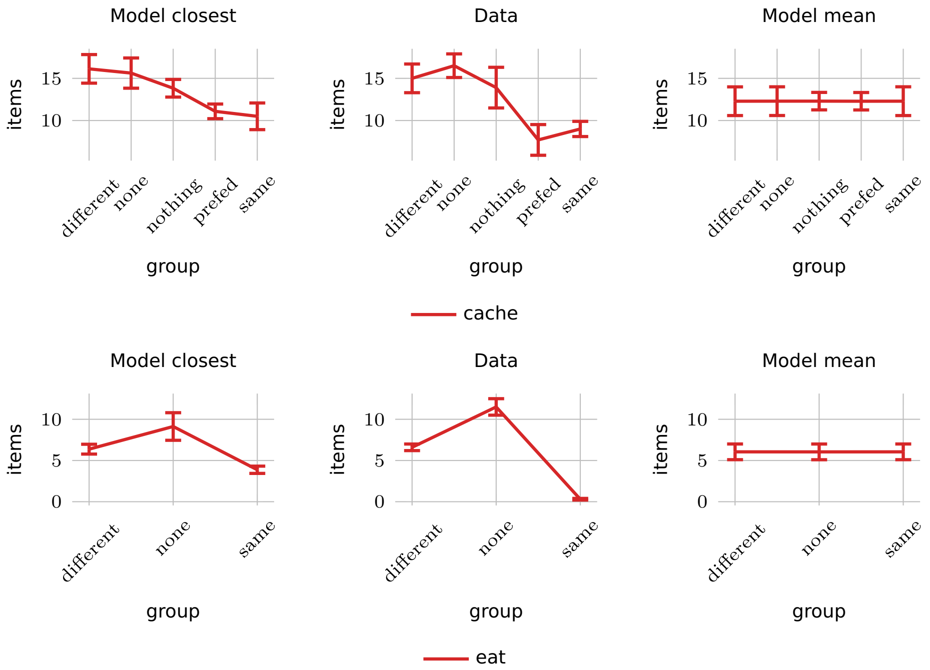

Figure S159: **Clayton99C exp3: No-Plasticity-No-Memory-No-Motivational-Control Model**

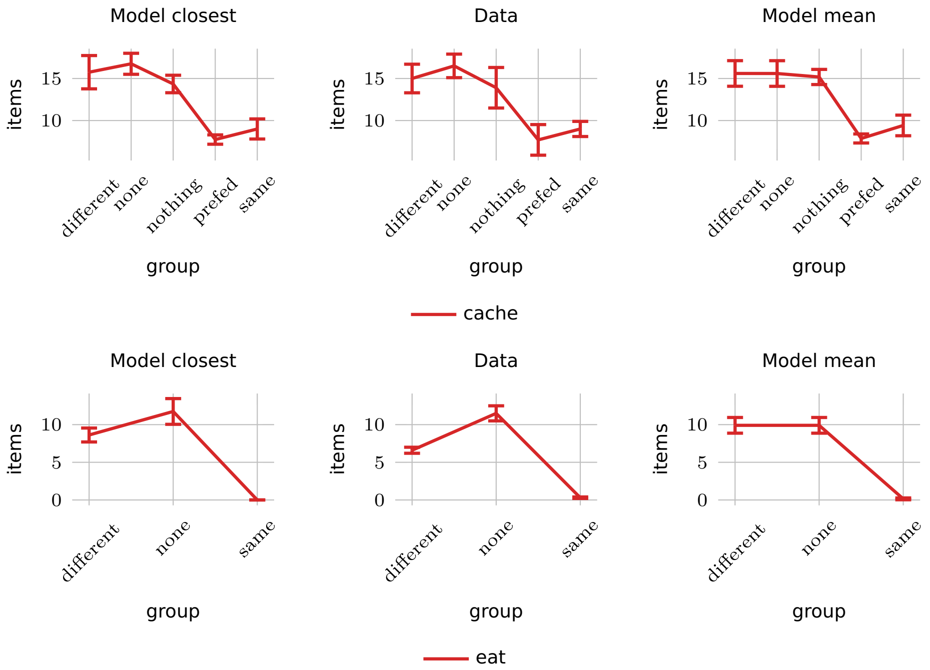

Figure S160: **Clayton99C exp3: No-Plasticity-No-Memory Model**

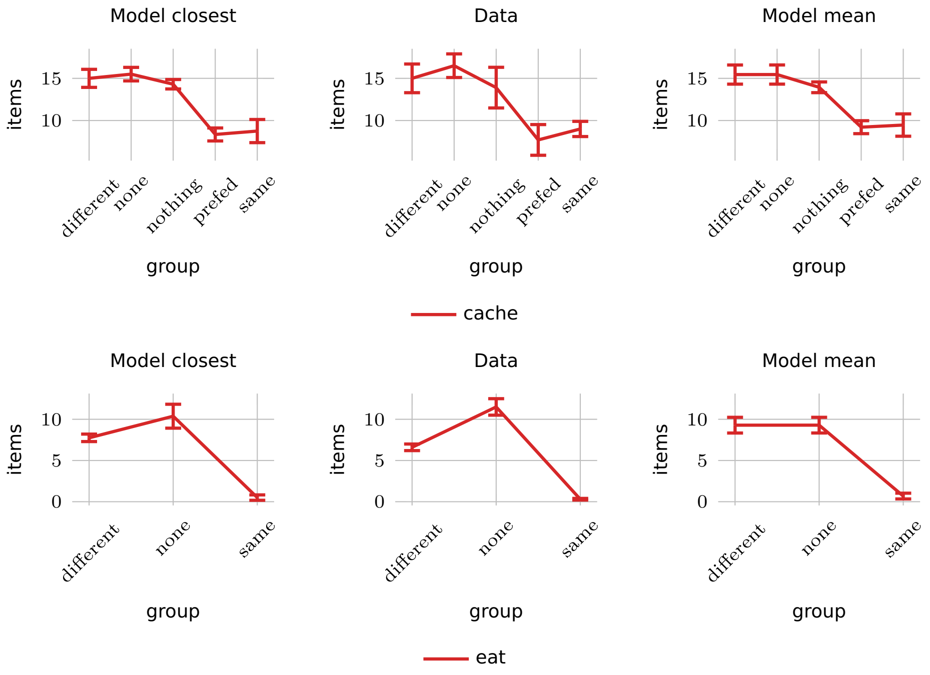

Figure S161: **Clayton99C exp3: No-Plasticity Model**

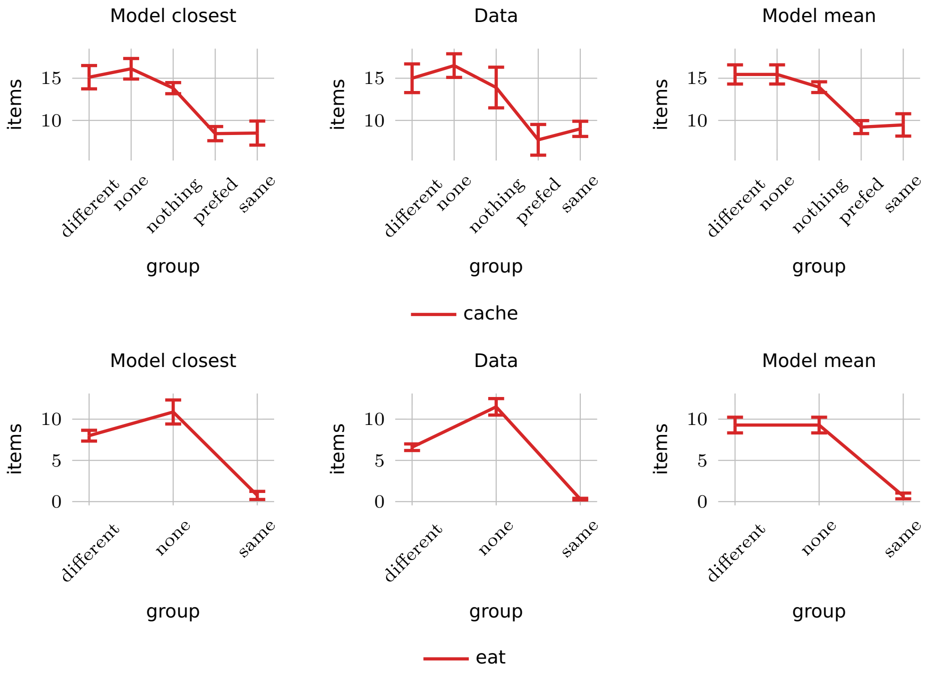

Figure S162: **Clayton99C exp3: Plastic Caching Model**

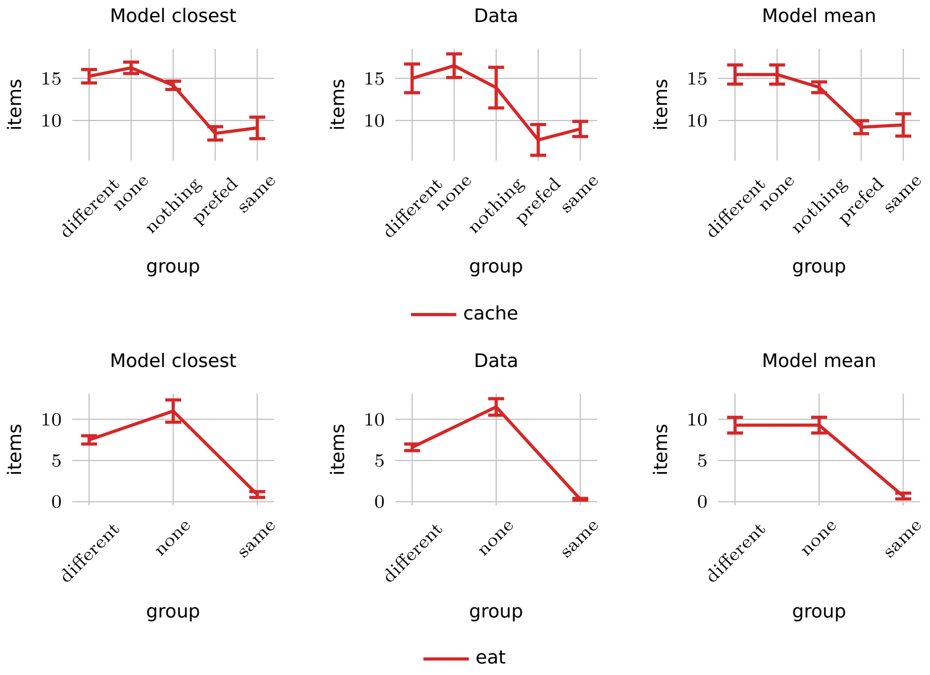

Figure S163: **Clayton99C exp3: Planning-By-Replay Model**

2.1.28 deKort07 exp1

Published in [9].

Major Finding

In the absense of alternative food items to cache, birds continue to cache mealworms, even if they are degraded or pilfered before recovery.

Comments

The caching rate during experiment 1b was much significantly lower than during experiment 1a.

Most Important Tests

Experiment b (group): $F(1, 14) < 1, p = 0.34$

Experiment a (group): $F(1, 14) = 1.26, p = 0.28$

Experiment a (trial): $F(5, 70) = 1.9, p = 0.11$

Total Number of Statistics

6 p-values, 48 means or sems.

Figures

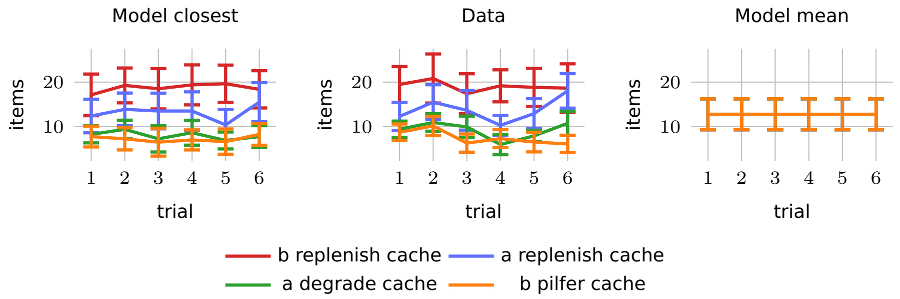

Figure S164: **deKort07 exp1: No-Plasticity-No-Memory-No-Motivational-Control Model**

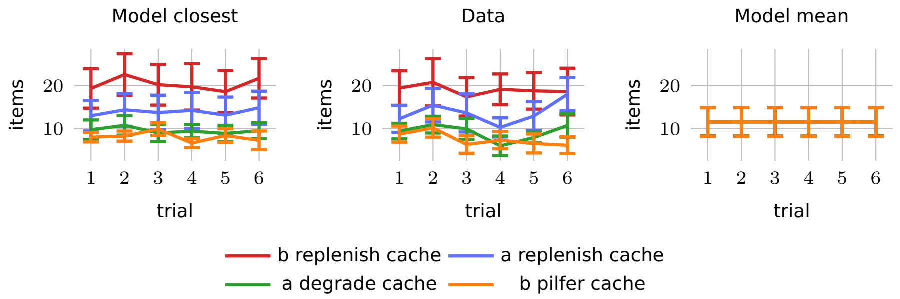

Figure S165: **deKort07 exp1: No-Plasticity-No-Memory Model**

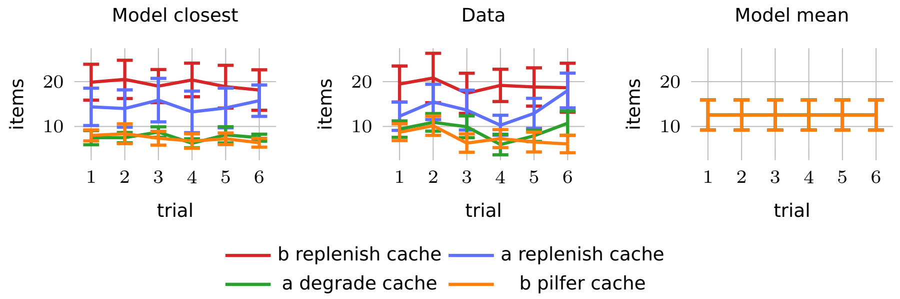

Figure S166: **deKort07 exp1: No-Plasticity Model**

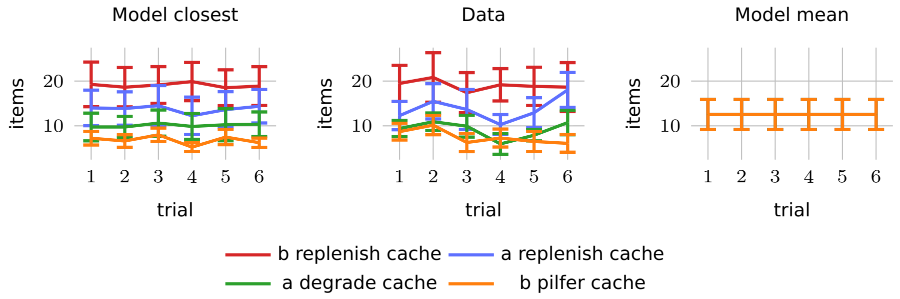

Figure S167: **deKort07 exp1: Plastic Caching Model**

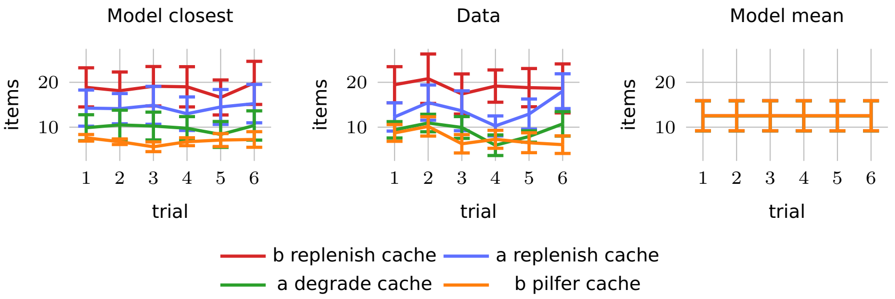

Figure S168: **deKort07 exp1: Planning-By-Replay Model**
